# Enhanced quantification of 64 pesticides in cannabis-derived oil by isotope-dilution LC-MRM-MS at regulatory decision thresholds

**DOI:** 10.1007/s00216-026-06661-5

**Published:** 2026-07-18

**Authors:** Raymond T. Suhandynata, Jiaxi Cai, Benjamin J. Spiegel, Thomas D. Marcotte, Igor Grant, Robert L. Fitzgerald, Jeremiah D. Momper

**Affiliations:** 1 Department of Pathology, University of California San Diego, San Diego, USA; 2 Center for Medicinal Cannabis Research Reference Laboratory, Skaggs School of Pharmacy and Pharmaceutical Sciences, University of California San Diego, San Diego, USA; 3 Shu Chien-Gene Lay Department of Bioengineering, Jacobs School of Engineering, University of California San Diego, San Diego, USA; 4 Center for Medicinal Cannabis Research, Department of Psychiatry, University of California San Diego, San Diego, USA

**Keywords:** Stable-isotope, Isotope-dilution mass spectrometry, Liquid chromatography-mass spectrometry, Cannabis, Pesticides

## Abstract

Cannabis legalization has created regulated markets requiring residual pesticide testing to protect public health. Regulatory programs establish action limits governing product release and enforcement, necessitating accurate quantification in analytically challenging matrices. Compliance is further complicated by the need to quantify dozens of compounds with widely varying physicochemical properties, often requiring multiple assays per product. Validated multiplex methods in representative matrices are therefore critical for defensible quantification at regulatory thresholds. We developed and validated a multiplex liquid chromatography-tandem mass spectrometry (LC-MS/MS) method for the simultaneous quantification of 64 pesticides in cannabis-derived hemp oil, employing external calibration with 58 stable isotope-labeled internal standards (SIL-IS) and 4 surrogate internal standards (IS) across 8.4 units of LogP. Validation followed Clinical & Laboratory Standards Institute (CLSI) guidelines (C62-A, EP05-A3, EP29-A) and Food and Drug Administration (FDA) guidance. All 64 pesticides demonstrated linear calibration (R^2^ ≥ 0.995), with accuracy of 80.0–120.0% (mean recovery = 101.9%) and precision of ≤ 15% coefficient of variation (%CV, mean %CV = 2.7%). Analytical measurement ranges extended to 20,000 parts per billion (ppb) for 62 components. Expanded measurement uncertainty ranged from ±2.3% to ±14.6% and remained ≤15% at 100 ppb for 57 of 64 pesticides. Systematic benchmarking of surrogate IS suitability across all analyte-IS pairings showed that performance correlated with chromatographic and physicochemical similarity, enabling identification of optimal surrogates and analytes requiring matched SIL-IS. This study illustrates an isotope-dilution approach for multiplexed pesticide quantification with structured uncertainty characterization to support accurate and defensible regulatory determinations at regulatory thresholds for cannabis.

**Graphical abstract:**

**Figure:**
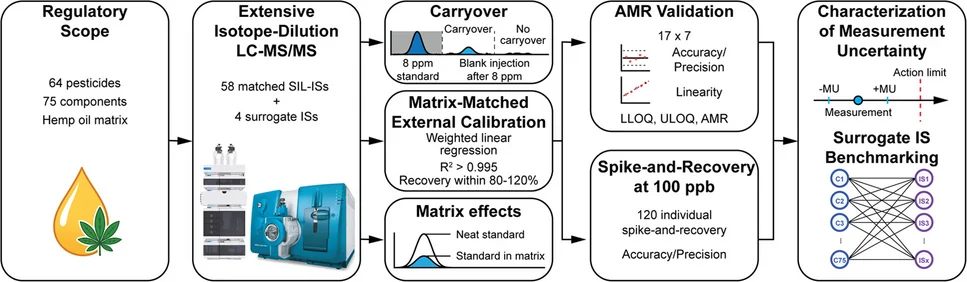

**Supplementary Information:**

The online version contains supplementary material available at https://doi.org/10.1007/s00216-026-06661-5.

## Introduction

Cannabis products are high-value agricultural commodities in multi-billion-dollar markets [1]. Most regulated cannabis markets require licensed laboratories to measure residual pesticides in legally distributed cannabis and cannabis-derived products in the interest of protecting public health and safety [2, 3]. In California (CA), the Department of Cannabis Control (DCC) mandates residual pesticide testing prior to commercial distribution and requires licensed laboratories to quantify pesticide concentrations relative to established regulatory limits [4]. Similar regulatory frameworks exist across multiple US states and internationally, including Canada’s federally regulated cannabis program and European Union pesticide residue regulations, although pesticide panels, product classifications, action limits (ALs), and reporting requirements vary by jurisdiction [4–8]. Non-compliance may result in significant economic losses for growers, manufacturers, and licensed distributors due to enforcement actions such as product destruction and recall [9]. Because compliance determinations depend on quantitative analytical measurements relative to established ALs, the reliability and interpretability of laboratory results carry substantial economic and legal implications, and play a central role in determining whether products may enter or remain in the legal marketplace [10]. Rigorous characterization of analytical performance and measurement uncertainty is therefore critical for evaluating the reliability of results at or near ALs, enabling regulatory decision-makers to proceed with compliance determinations confidently and to take enforcement action when warranted [3, 11, 12].

Cannabis-derived products encompass highly heterogeneous matrices, including dried plant material, oils, concentrates, distillates, and infused products, each with distinct physicochemical properties [12]. Oil-based matrices (e.g., cannabis oil or hemp oil) contain high concentrations of lipids, cannabinoids, terpenes, and other co-extracted compounds that contribute to matrix-dependent variability affecting quantitative measurement [13–16]. Although recent advances in analytical approaches have enabled improved multiplex detection of pesticides across cannabis-derived matrices using liquid chromatography-tandem mass spectrometry (LC-MS/MS) and gas chromatography-tandem mass spectrometry (GC-MS/MS) [17–25], significant analyte-specific matrix effects persist, adversely impacting quantitative accuracy and precision [26]. These challenges are amplified in non-polar oil and concentrate matrices, where analytes spanning wide ranges of polarity and physicochemical behavior may co-elute or exhibit differential ion suppression or enhancement, leading to errors when quantifying targeted analytes [18]. While currently published methods enable multiplex detection of pesticides at concentrations relevant to regulatory ALs, reports describing comprehensive development, validation, and characterization of method-specific performance metrics, including accuracy, precision, linearity, matrix effects, and analytical measurement uncertainty, remain limited [17]. Consequently, there is a need for a structured framework to guide the development of analytically robust methods.

Regulatory and accreditation frameworks such as ISO/IEC 17025 establish essential quality system requirements governing laboratory competence, method validation, and ongoing performance monitoring [27]. Complementary validation guidelines, including European Commission SANTE pesticide method validation criteria [28], AOAC International performance standards [29], and FDA method validation guidance [30], define expectations for analytical performance characteristics such as accuracy, precision, and sensitivity. Collectively, these frameworks establish essential quality infrastructure and minimum performance expectations necessary to support regulatory testing. However, detailed and standardized evaluation protocols, including experimentally defined replicate designs, statistical modeling approaches, and clearly articulated acceptance criteria for bias, precision, and measurement uncertainty, are not uniformly prescribed across these frameworks. As a result, laboratories may apply variable validation strategies and statistical methodologies, potentially leading to inconsistencies in the depth and rigor of analytical performance characterization.

In contrast, validation frameworks developed by the Clinical and Laboratory Standards Institute (CLSI) provide structured statistical methodologies and well-defined evaluation protocols for assessing analytical precision, bias, and measurement uncertainty [31–35]. These guidelines specify experimental designs, replicate requirements, and statistical approaches that promote consistency and transparency in method validation. While originally developed for clinical laboratory medicine, the structured and quantitative nature of CLSI validation principles offers value beyond their intended field and may serve as a useful framework for enhancing metrological rigor across interdisciplinary regulatory testing environments. Applications of similarly rigorous statistical validation approaches provide an opportunity to improve analytical transparency, strengthen inter-laboratory comparability, and increase confidence in regulatory compliance determinations.

LC-MS/MS using triple quadrupole multiple reaction monitoring has emerged as the dominant analytical platform for multi-residue pesticide testing due to its analytical sensitivity, selectivity, and multiplexing capability [17, 18]. However, in complex matrices such as cannabis oils and concentrates, co-extracted lipids, cannabinoids, terpenes, and other constituents may alter analyte recovery, chromatographic behavior, and ionization efficiency, leading to matrix effects that reduce the accuracy and precision of results [17, 18, 26, 36]. For example, co-eluting matrix components may suppress or enhance analyte ionization in the source, resulting in systematic underestimation or overestimation of pesticide concentrations if these effects are not adequately corrected. Therefore, existing methods commonly employ internal standards (ISs), compounds added at a known concentration to each sample prior to analysis, to compensate for variability introduced during sample preparation, analyte extraction, chromatographic separation, and matrix effects [37]. Quantification is performed by comparing the signal of each target analyte to that of its corresponding IS, typically using analyte-to-IS response ratios to construct external calibration curves and determine the concentration of an analyte in an unknown sample [37, 38]. Stable isotope-labeled internal standards (SIL-IS) enable isotope dilution mass spectrometry (IDMS), which is considered the most robust quantitative approach in mass spectrometry [38]. SIL-ISs are structurally identical to their analytes but differ in mass, allowing co-elution while remaining distinguishable by mass spectrometry. By closely matching analyte behavior, they correct for variability in extraction, matrix effects, and instrument response [38–41]. As a result, isotope dilution provides superior quantitative accuracy and precision and is widely used in highly regulated analytical domains, including clinical laboratory medicine, pharmaceutical bioanalysis, forensic testing, and environmental testing. However, isotope-labeled analogs are not commercially available for all analytes, and chemically similar but not identical compounds are commonly employed in their absence as surrogate ISs. Unfortunately, the quantitative performance of surrogate ISs for most regulated pesticides in complex cannabis matrices remains incompletely characterized, particularly within high-density multiplex assays in non-polar matrices such as cannabis/hemp oil.

An additional challenge in cannabis residual pesticide testing is the absence of higher-order reference measurement procedures and matrix-matched certified reference materials (CRMs) for most analytes. In metrological frameworks defined by the International Vocabulary of Metrology, measurement traceability is established through calibration hierarchies linking routine methods to reference measurement procedures and certified reference materials [42]. Example matrix-matched CRMs have been harmonized in foodstuff matrices [43], and CRMs for toxic elements, including arsenic, cadmium, and lead, have been characterized in a cannabis leaf matrix [44]. However, such CRMs are unavailable for pesticide residues in cannabis matrices, and quantitative measurements are therefore method-defined. Under these circumstances, structured characterization of analytical measurement uncertainty becomes essential for establishing the reliability and interpretability of reported results. Statistical frameworks developed by CLSI and Eurachem provide formal methodologies for uncertainty estimation by integrating contributions from analytical imprecision and systematic bias [31, 45]. These approaches provide a transparent and statistically grounded framework for characterizing analytical performance and supporting defensible interpretation of results near regulatory decision thresholds.

Although prior studies have demonstrated the feasibility of multi-residue pesticide analysis in cannabis flower and cannabis-derived oils, important analytical questions remain unresolved in highly multiplexed assays in these complex matrices [24, 26]. Prior methods have generally focused on demonstrating that acceptable validation performance for a large majority of pesticides can be achieved using matrix-matched calibration with limited use of SIL-ISs. However, the extent to which SIL-ISs can improve the accuracy and precision of measurements has not been fully explored. Furthermore, meeting the demands of robust accuracy, precision, and analytical measurement uncertainty at regulatory decision thresholds has proven difficult in practice [24].

This study addresses these gaps in a high-lipid hemp oil matrix by developing and validating a multiplex isotope-dilution LC-MS/MS method for the quantitative analysis of 64 regulated pesticides. By integrating isotope dilution, systematic surrogate IS benchmarking, and structured characterization of analytical measurement uncertainty in accordance with CLSI and FDA guidelines [30–35], we establish a comprehensive analytical framework for multi-residue pesticide quantification in cannabis and cannabis-derived matrices, supporting more defensible compliance determinations in regulated markets. Notably, robust characterization and validation of this method enabled systematic benchmarking of surrogate IS against SIL-IS, which are widely regarded as the most robust internal standard reference for absolute quantification in complex matrices. This analysis further demonstrates that SIL-ISs are essential for robust multi-residue pesticide quantification in complex lipophilic matrices such as hemp oil. Meaningful surrogate IS benchmarking requires precise quantitative measurements, broad isotope-dilution coverage, and adequate chromatographic separation to isolate analyte-specific matrix effects; aspects which have not been comprehensively addressed in prior multi-residue pesticide studies for hemp oil.

## Methods

### Reference materials and hemp oil matrix

Calibrators and spike-and-recovery material were prepared in extracted hemp oil from independent lots procured from AccuStandard (Supplementary Fig. 1). 1.2 g of hemp oil (Whole Foods, Cat. #9765820102) was extracted with 6 mL of acetonitrile (ACN), which is used as a 20% matrix stock. A working extracted matrix is generated by diluting 20% matrix stock with methanol (MeOH) and 18 MΩ·cm water (H_2_O) to a final composition of 75:12.5:12.5 (H_2_O:ACN:MeOH, v/v/v), corresponding to a 40-fold dilution of the original matrix (2.5% matrix). Stable isotope-labeled (SIL) internal standards (IS) and surrogate IS were obtained from various vendors (Supplementary Fig. 1) and prepared in 2.5% matrix.

### Preparation of calibrators and internal standards

Working stock solutions for standard materials were prepared by combining three pesticide standard mixes (AccuStandard: Cat. #CP-CA-01, CP-CA-03, and CP-CA-04; Supplementary Fig. 1) and diluting the combined mixture to 100 µg/mL (per pesticide) in ACN. The primary IS stock solution was prepared by combining individual IS solutions in ACN. IS stock concentrations are shown in Supplementary Table 2.

The initial calibrator working stocks were prepared by spiking target analytes into the extracted matrix. 20 µL and 15 µL of calibrator stock were added to calibrators 18 and 17, respectively. All 18 calibrators initially contained 500 µL of extracted matrix solution, except calibrator 18 (980 µL) and calibrator 17 (985 µL). Calibrators were prepared by performing twofold serial dilutions on the standard working stock in 2.5% matrix. Two independent serial dilution sets were prepared, and each set was serially diluted by transferring 500 µL of the preceding vial to the next vial in sequence (18 → 16 → 14; 17 → 15 → 13).

A working IS solution (Supplementary Table 2) was freshly prepared for each dilution set by combining 120 µL of primary IS stock with 67.5 µL MeOH, 187.5 µL 20% matrix stock, and 1125 µL H_2_O. Calibrators were isotope-diluted using 50 µL of this working IS solution, followed by the addition of 450 µL of 2.5% matrix to complete the preparation. Calibrator concentrations in-vial and in-sample for each component can be found in Supplementary Table 3 and Supplementary Table 4, respectively.

### Liquid chromatography multiple reaction mass spectrometry method

The liquid chromatography method used two solvents: Mobile Phase A (5 mM ammonium formate (NH_4_HCOO)/0.1% formic acid/H_2_O) and Mobile Phase B (5 mM NH_4_HCOO/0.1% formic acid/MeOH). Chromatography was obtained using an Agilent 1290 Infinity II UHPLC system with a Waters CORTECS C18 Column (90 Å, 1.6 µm, 2.1 mm X 150 mm; Cat# 186007096) with a Waters CORTECS C18 VanGuard Pre-column (90 Å, 1.6 µm, 2.1 mm X 5 mm; Cat#186007123) maintained at 45 °C by the column heater. The flow rate was 0.35 mL/min across the entire 40.01-min chromatographic gradient (Supplementary Table 5). The injection volume for all analyses was 10 μl with the exception of the analyses for obtaining extended analytical measurement ranges (AMRs). Multiple reaction monitoring (MRM) was performed on a SCIEX QTRAP 6500^+^ triple quadrupole mass spectrometer operating in positive electrospray ionization mode (ESI). Source parameters were as follows: Spray Voltage: 5.5 kV, Source Temperature: 325 °C, Ion Source Gas 1: 65 psi, Ion Source Gas 2: 65 psi, Curtain Gas: 35 psi, CAD Gas: 10 (arbitrary unit). MRM parameters are listed in Supplementary Table 6.

### External calibration

Linear regression of calibration curves was performed using ordinary least squares regression of analyte-to-IS peak area ratios. Acceptance criteria required coefficients of determination (R^2^) ≥ 0.995 and accuracy within ± 20%. Most calibration curves employed 1/x weighting; Pyridaben was unweighted, and the origin was included for the linear regressions of Spinetoram J, Spinetoram L, and Spinosyn A. Selections of weighting factors and the inclusion or exclusion of the origin were performed on an analyte-specific basis using best-fit regression model evaluation to minimize systematic bias and optimize calibration accuracy across the AMR.

### Characterization and establishment of the AMR: 17 × 7 spike-and-recovery

Accuracy, precision, and analytical measurement uncertainty (MU) were evaluated in accordance with CLSI guidelines (C62-A and EP29-A) [31, 33] across the AMR using a 17 × 7 spike-and-recovery scheme. 7 independent spikes were prepared at 17 concentration levels from a pesticide working stock (10 µg/mL) in ACN:MeOH (50:50, v/v) and spiked into 100 mg of hemp oil (n = 119). Actual concentrations (in-vial and in-sample) for each spike level can be found in Supplementary Table 7 and Supplementary Table 8. Hemp oil samples (100 mg) were extracted using 0.5 mL of ACN and diluted 40-fold to a final solvent composition of 75:12.5:12.5 (H_2_O:ACN:MeOH, v/v/v), followed by LC-MS/MS analysis. Spike-and-recovery experiments were performed across a concentration range of 0.244–4000 parts per billion (ppb) in-sample to establish the primary AMR. An additional spike at up to 20,000 ppb in-sample (500 ng/mL in-vial), five-fold above the highest calibrator, was performed to evaluate extension of the AMR by reducing the autosampler injection volume from 10 µL to 1 µL. Acceptance criteria throughout the primary and extended AMR were defined as recovery of 80–120% and precision (%CV) ≤ 15% at each concentration level. For each component, the lower bound of its primary AMR was assigned as the lower limit of quantification (LLOQ), and the upper bound as the upper limit of quantification (ULOQ). Additionally, extended ULOQs were assigned using the upper bounds of the extended AMRs. The acceptance criteria for LLOQs and ULOQs include having a recovery of 80–120% and %CV ≤ 15%. In addition, the LLOQ was required to meet a minimum signal-to-noise ratio of 10:1. Due to degradation observed for Captan in the spike-and-recovery standard material, value assignment for Captan was performed using freshly prepared standards from powder.

### Matrix spike and recovery at 100 ppb in hemp oil matrix

Accuracy, precision, and MU at the lowest regulatory AL described in the CA DCC Text of Regulations for both inhalable and non-inhalable cannabis products were evaluated by performing 120 independent spike-and-recoveries at 100 ppb in hemp oil [4]. Matrix spikes were prepared by spiking in 20 µL of a 0.5 ng/mL pesticide working stock solution prepared in ACN:MeOH (50:50, v/v) to 100 mg of hemp oil. Spikes were analyzed across three independent analytical batches (40 spikes per batch) to assess accuracy, precision, and MU. Sample extraction and analysis were performed as described above.

### Assessment of carryover

Carryover was evaluated by injecting a standard (H1 and H2) at 200 ng/mL in-vial (8 ppm in-sample) and the lowest calibrators (L1, L2, L3, and L4) within each component’s primary AMR in the following sequence: L1, L2, H1, H2, L3, L4. By comparing the mean quantification of the lowest calibrators before (L1 and L2) and after (L3) injecting a 200 ng/mL in-vial (8 ppm in-sample) standard (H1 and H2), the percent carryover relative to each compound’s LLOQ can be calculated as follows (Eq. 1):

1 $$\begin{array}{c}\% Carryover=\frac{L3-\frac{L1+L2}{2}}{\left[LLOQ\right]} \times 100\end{array}$$

where L1, L2, and L3 are calculated concentrations of the corresponding injection and [LLOQ] is the nominal concentration of the LLOQ for each component. L4 was included in the injection sequence to monitor potential persistent carryover across multiple injections qualitatively and was not used in the calculation. Acceptance criteria for carryover at the LLOQ was ± 20%, in accordance with CLSI guidelines [33].

### Assessment of matrix effects

Matrix effects (ME) were evaluated by preparing pesticide standards at 12.5, 25, 50, and 100 ng/mL in both post-extracted matrix (PEM) and solvent-only no-matrix (SNM) conditions. Samples were injected in the sequence SNM1, PEM1, PEM2, SNM2. The injection sequence was designed with neat standards bracketing matrix spikes to reveal systematic errors such as run effects, analyte instability, and baseline drifts. ME was expressed as % recovery of the analyte from PEM and calculated using Eq. 2:

2 $$\begin{array}{c}\%Matrix\;Effect=\frac{(PEM1+PEM2)/2}{(SNM1+SNM2)/2}\times100\end{array}$$

The PEM and SNM terms are integrated peak areas for each analyte’s quantifier ion. Analytes with ME between 80 and 120% were considered to exhibit minimal ME. For analytes demonstrating instability in solvent or extracted matrix (Acequinocyl, Bifenazate, Captan, and Phosmet, Supplementary Fig. 2), ME was only evaluated at 50 ng/mL in-vial.

### Calculation of analytical measurement uncertainty

All MUs reported are limited to the analytical components. Pre-analytical MU and MU in the standard materials were not considered. MU was evaluated through a top-down approach as specified by CLSI guidelines (EP29-A) [31]. Two components were considered in calculating the combined standard MU. The first component (MU_precision_, Eq. 3) is defined as the total estimated imprecision (%CV) of the measurements, and the second component (MU_estimated mean_, Eq. 4) comprises the standard uncertainty in the estimation of the mean. Since the observed biases in individual measurements were small by the nature of our exclusion criteria (within 20% of the assigned nominal value), the reported values and calculated means were not corrected for biases, and the above-stated two-component MU is sufficient, especially after expansion with a coverage factor. Therefore, the expanded combined standard MU (MU_combined_) is eventually calculated using Eqs. 5 and 6 with a coverage factor k = 2.

3 $$\begin{array}{c}{MU}_{precision}=\%CV\end{array}$$

4 $$\begin{array}{c}{MU}_{estimated\;mean}\left(n\right)=\frac{\%CV}{\sqrt n}\end{array}$$

5 $$\begin{array}{c}{MU}_{combined}=\sqrt{{MU}_{precision}^2+{MU}_{estimated\;mean}^2}\end{array}$$

6 $$\begin{array}{c}{Expanded\;MU}_{combined}=k\times{MU}_{combined},k=2\end{array}$$

MUs were calculated for both the 17 × 7 spike-and-recovery across the AMR and the 120 spike-and-recovery at 100 ppb. For the 17 × 7 dataset, all Cals and QCs were made on the same day (within the same batch); since the injection sequence ran continuously for 5 days to complete the analysis, the reported MUs are consistent with an estimate of inter-day MUs across the AMR. The 120 spike-and-recovery dataset was collected across 3 batches of 40 spike-and-recovery prepared on different days, which is consistent with an estimate of inter-batch MUs.

### Benchmarking of surrogate internal standards

Benchmarking of surrogate ISs was conducted by substituting the SIL-IS/surrogate specified in Supplementary Table 9 for each component with all other ISs then iteratively fitting 1/x weighted linear regression curves for all analyte-IS pairs. Surrogate ISs were benchmarked against the SIL-IS for the corresponding analyte, if available. For analytes without an available SIL-IS, the nominal assigned value was used to calculate recovery. For each analyte component and candidate IS, signal normalization was performed by computing the analyte-to-IS peak area ratio (*R*) (Eq. 7):

7 $$\begin{array}{c}R= \frac{{AUC}_{analyte}}{{AUC}_{IS}}\end{array}$$

All calibration curves were fit using linear regression with a 1/x weighting scheme. Each analyte-IS calibration curve was required to meet the acceptance criteria of R^2^ ≥ 0.99, a minimum of 6 calibrator levels, and a maximum absolute percent bias of 20% from the nominal value across all calibrators (i.e., % recovery between 80 and 120%). Curves failing any of these criteria were excluded from downstream evaluation.

Iterative curve fitting was performed in 2 stages using a Python script with the following algorithm:

In Stage 1, a contiguous search strategy was used to choose calibrator levels for each analyte by fitting integrated peak area vs nominal calibrator concentration without IS normalization. The data are sorted by concentration, and the algorithm evaluates every continuous concentration window (from largest to smallest), scoring each candidate by threshold criteria (max bias and R^2^) and then by quality (more points, wider range, lower bias, better fit), so the selected set is a contiguous set of calibrators.

In Stage 2, the script fits IS-normalized curves for each analyte-IS pair using only the Stage-1-selected concentrations but applies a greedy refinement: it starts from the available selected points, repeatedly removes the point with the worst bias, and refits until the criteria are met or the minimum point count is reached.

Evaluation of each surrogate calibration curve was performed by applying the curve to both the 17 × 7 and 120 AL spike datasets and computing the bias of the surrogate quantification relative to the SIL-IS quantification or the nominal assigned value. Bias calculations were restricted to concentrations within the established primary AMRs shown in Supplementary Table 10. For each analyte-IS pair, the maximum absolute % bias across all retained samples (4 batches in total) was used as the worst-case performance metric to evaluate surrogate IS performance with maximum stringency (Supplementary Table 11).

### Estimation of analytical measurement uncertainty as a function of the number of replicate measurements

To simulate the number of replicate measurements required for a stable estimate of recovery, we evaluated the convergence behavior of sampled means using the 120 independent spike-recovery measurements at the lowest CA AL. For each analyte, the arithmetic mean of all 120 measurements was treated as the reference value (the “true” mean). Finite-sample behavior was assessed by random permutation of the 120 measurements (*R* = 300 permutations) using Monte Carlo simulation, and sampled means were calculated for replicate counts n=1-120. At each n, the empirical distribution of the sampled mean across the 300 permutations was summarized by its 95% confidence interval (CI), and MU was expressed as the percent half-width of that CI (Eq. 8):

8 $$\begin{array}{c}{MU}_{empirical}\left(n\right)=\frac{{\overline x}_{97.5th\;percentile}\left(n\right)-{\overline x}_{2.5th\;percentile}\left(n\right)}{2\times{\overline x}_{120}}\times100\end{array}$$

The calculated MU_empirical_(n) simulates the variability in the reported mean when only n measurements were performed.

In parallel, a precision-only uncertainty model was constructed using the experimentally determined CV for each analyte. Assuming independent measurements and negligible bias contribution to the uncertainty of the mean, the predicted MU was modeled as follows (Eq. 9):

9 $$\begin{array}{c}M{U}_{model}\left(n\right)=k\times \frac{CV}{\sqrt{n}} \end{array}$$

This model reflects an expected $1/\sqrt{n}$ scaling of uncertainty in the mean of repeated measurements.

Assuming normal distribution of errors, a coverage factor, k, of 2 does not correspond exactly with the 95% confidence interval. The exact coverage factor is 1.96. Nevertheless, CLSI and international standards, such as the Guide to the Expression of Uncertainty in Measurement (GUM) published by the Joint Committee for Guides in Metrology (JCGM) [31, 42], round up to 2 for practical applications, and the calculations here were made in accordance with this convention.

A comparison of the variation in empirical sampling and the modeled measurement uncertainty was performed by computing the coefficient of determination as follows (Eq. 10):

10 $$\begin{array}{c}{R}^{2}=1-\frac{{\sum}_{n=1}^{120}{\left({MU}_{empirical}\left(n\right)- {MU}_{model}\left(n\right)\right)}^{2}}{{\sum}_{n=1}^{120}{\left({MU}_{empirical}\left(n\right)-\overline{{MU }_{empirical}}\right)}^{2}} \end{array}$$

This analysis quantifies how closely the empirical sampling follows the theoretical $1/\sqrt{n}$ precision decay, assuming a normal error distribution. As %CV approaches the intrinsic MU, the R^2^ value approaches 1. In other words, an R^2^ value close to 1 indicates that the number of measurements made (n = 120) was sufficient for determining the intrinsic MU.

## Results

### Development of a liquid chromatography method to separate 75 pesticide components

A multi-residue LC-MS/MS pesticide panel was evaluated for 64 of the 66 pesticides described in the CA DCC Text of Regulations (Supplementary Table 12). Chlordane and pentachloronitrobenzene are highly nonpolar and were excluded from the analysis due to their poor ionization efficiency under electrospray ionization conditions; these compounds have been measured by other studies using GC-MS/MS or LC-Atmospheric Pressure Chemical Ionization (APCI)-MS/MS in other cannabis and food product matrices [46–48]. Of the 64 pesticides, 52 are single-compound pesticides, 8 exist as mixtures of stereoisomers, and the remaining 4 exist as mixtures of structurally related compounds. For pesticides comprised of isomers that were fully chromatographically resolved (e.g., (E)- and (Z)-Permethrin, (E)- and (Z)-Dimethomorph, and (E)- and (Z)-Mevinphos), individual isomers were evaluated and quantified separately and monitored as a total of 6 components. For pesticides with isomers that were not fully resolved (Cypermethrin, Cyfluthrin, Prallethrin, Propiconazole, and Spiroxamine), the chromatographic areas of the overlapping isomeric peaks were integrated as a single component. Pesticides containing chemically similar compounds (Pyrethrins: 6 components; Spinetoram: 2 components; Spinosad: 2 components; Abamectin: 2 components) were evaluated at the component level based on their chromatographic behavior. The certified reference materials for pesticides comprising multiple components were supplied as mixtures of the components at assigned ratios. Specifically, Permethrin was supplied as a 55:45 E/Z isomer mixture, Dimethomorph as a 43:57 E/Z isomer mixture, Mevinphos as a 89:11 E/Z isomer mixture, Pyrethrins as a 56.1:27.8:4.0:2.7:5.7:3.8 (Pyrethrin I/Pyrethrin II/Jasmolin I/Jasmolin II/Cinerin I/Cinerin II) compound mixture, Spinetoram as a 71.6:28.4 J/L compound mixture, Spinosad as a 81.2:16.9 A/D compound mixture, and Abamectin as an 80.7:19.3 B1a/B1b compound mixture. Therefore, 75 individual components representing 64 pesticides were monitored, and, where appropriate, component-level responses were aggregated to support reporting of total pesticide concentrations in accordance with the CA DCC Text of Regulations.

**Fig. 1:**
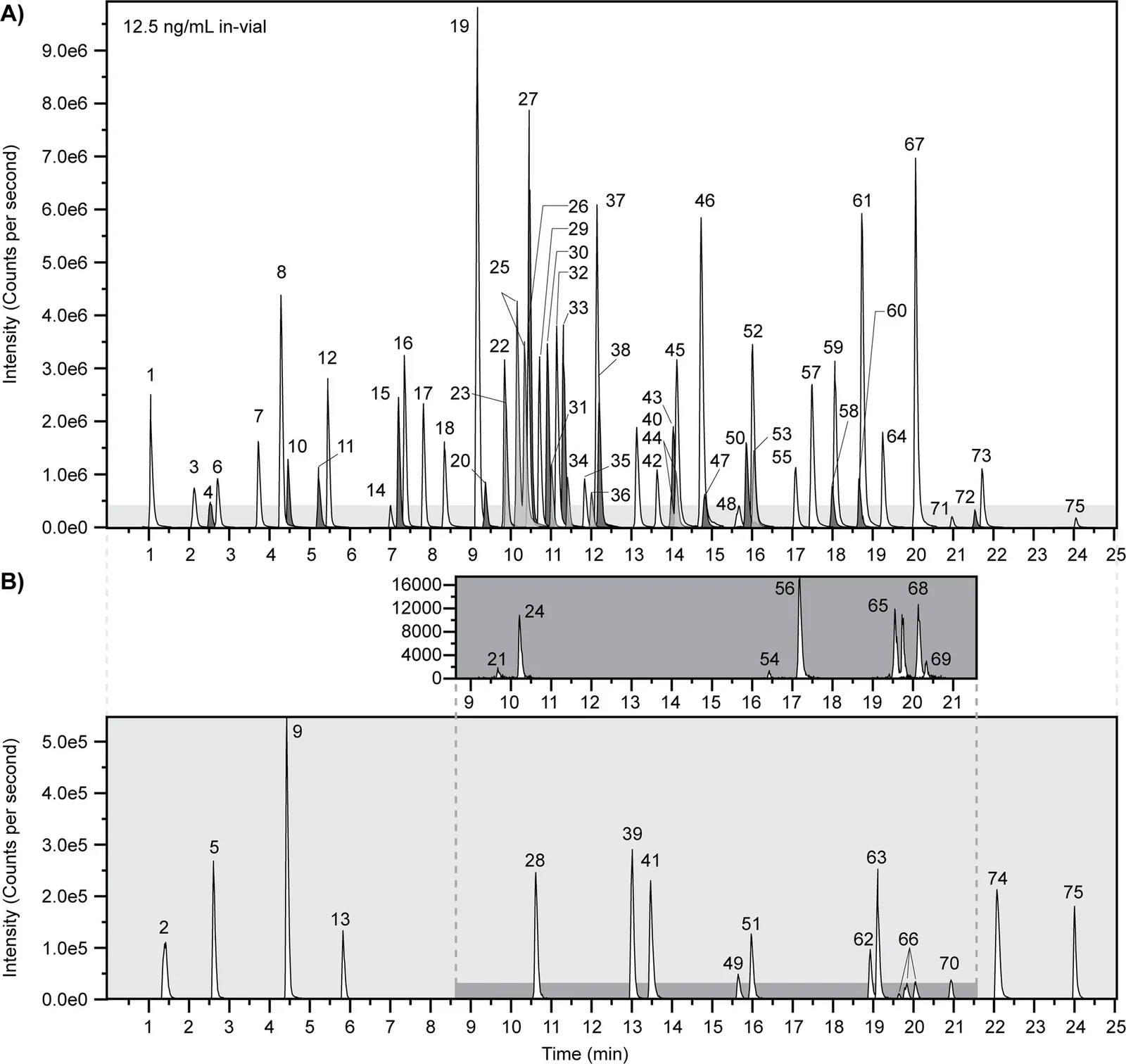
Extracted Ion Chromatogram (XIC) of 75 Pesticide Components. XICs for each analyte at level 12 (64 pesticides each at 12.5 ng/mL in-vial, refer to Supplementary Table 3 for individual component concentrations) were overlaid. The x-axis indicates retention time (min) and the y-axis indicates signal intensity (counts per second). **A** and **B** share the same x-axis. Two inset panels are shown in **B**, each with a different y-axis scale to help visualize varying degrees of ionization efficiency. Each XIC trace is labeled with a number corresponding to the XIC index listed in Supplementary Table 9

Development of the LC method was directed toward maximizing the separation of analytes, which span 8.4 units of LogP. The LC method sufficiently separates 75 components (Fig. 1 and Supplementary Table 9), 61 SIL-IS components, and 4 additional surrogate pesticides (Allethrin, Danitol, Ivermectin, and Emamectin), which are utilized as surrogate ISs (Supplementary Fig. 1 and Supplementary Table 9); totaling 140 components.

The LC method employs a multi-slope gradient elution profile across 25 min (Supplementary Table 5). A wide range of ionization efficiencies, exceeding 3 orders of magnitude in signal/chromatographic peak intensity, was observed across the 75 components. Extracted chromatograms were organized into 3 panels to illustrate the chromatographic behavior of components with favorable (Fig. 1A), moderate (Fig. 1B; lower panel), or poor (Fig. 1B; upper panel) ionization efficiencies.

### Extensive isotope dilution supports accurate and precise quantification

**Fig. 2:**
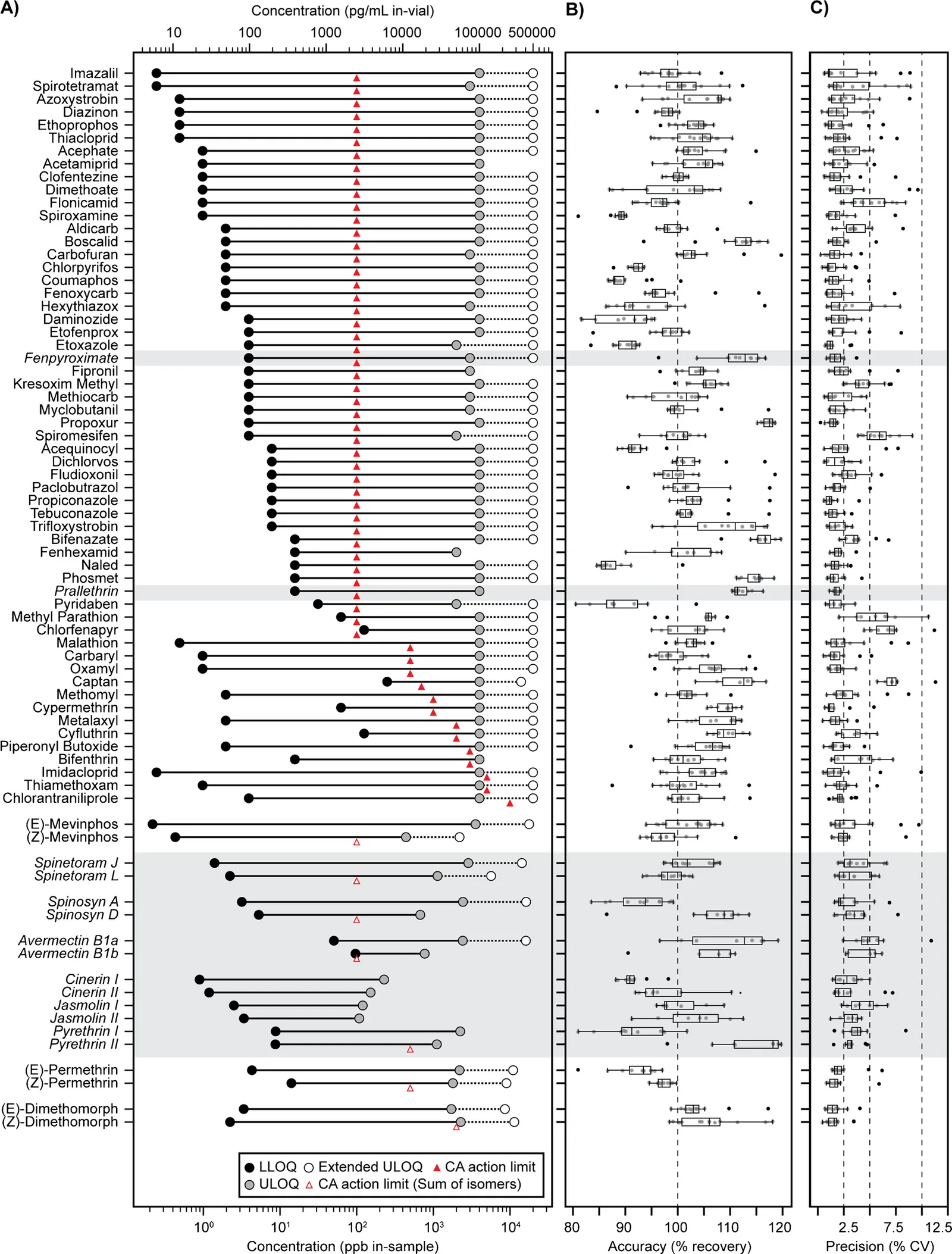
Analytical measurement range (AMR), accuracy, and precision for all analytes. **A** AMRs are shown as horizontal bars (lower limit of quantification [LLOQ] to upper limit of quantification [ULOQ]); dashed extensions indicate the extended analytical measurement range (AMR). Circles mark the LLOQ (solid black), ULOQ (gray fill), and extended ULOQ (white fill). California action limits (ALs) are indicated by red triangles; Due to the lack of defined ALs for each isomer, isomers sharing a summed AL are marked with white-filled triangles with red outline. Concentrations are on a logarithmic scale (lower x-axis: parts per billion [ppb] in-sample; upper x-axis: pg/mL in-vial). Grey shading indicates analytes utilizing a surrogate instead of its stable isotope-labeled (SIL) analog as internal standard (IS). **B** Box-and-whisker plots of accuracy (% recovery) across all calibration levels for each analyte. Boxes span the interquartile range (IQR); whiskers extend to 1.5 × IQR; outliers are shown as filled black circles. Dashed line is at 100% recovery. **C** Box-and-whisker plots of the coefficients of variation (%CVs) across all calibration levels. Dashed line at 2.5, 5, and 10% CVs

The AMRs for 75 components are shown in Fig. 2A and Supplementary Table 10. 61 of the 75 components were isotope-diluted using a stable isotope-labeled analog IS (^2^H, ^13^C, ^15^N, or ^18^O), while 14 components utilized a surrogate IS (Supplementary Table 9). All analytes were injected after serial dilution at 17 concentrations to obtain data for the construction of calibration curves (Supplementary Table 3). These curves were then manually inspected to ensure appropriate inclusion/exclusion of calibrators (see acceptance criteria in “Methods”).The minimum number of calibration levels utilized across 75 calibration curves was 7 for Avermectin B1b; the maximum was 18 for (E)-Mevinphos, Imazalil, and Imidacloprid; and the median was 13. 27 of the 75 components utilized 15 or more calibrators; another 49 utilized 10 or more; and the remaining 8 utilized at least 7 calibrators. Strong linearity was observed across the calibration range for all analytes, with a minimum coefficient of determination (R^2^) of 0.9951 and a median R^2^ of 0.9983. 36 of the 75 (48%) components demonstrated linearity across at least three orders of magnitude within the primary AMR.

The CA DCC Text of Regulations for residual pesticide testing in inhalable cannabis products specifies that laboratories must be able to quantify Category I pesticides at 100 ppb in-sample or below. For Category II pesticides, individual ALs vary (Supplementary Table 12). For the purpose of this manuscript, we refer to the 100 ppb requirement as the AL for Category I pesticides. Therefore, the lowest prescribed AL across 66 regulated pesticides is 100 ppb in-sample. In accordance with these regulations, pesticide concentrations reported here reflect regulatory measurands and are defined as one of the following: single analyte concentration, the summed concentrations of pesticide isomers, or the summed concentrations of chemically similar analytes. The lower end of the calibration ranges for all pesticides extended to at least 100 ppb in-sample, with the exception of Captan, Chlorfenapyr, and Cyfluthrin. Of these 3 analytes, the lowest calibration concentrations utilized for Captan (250 ppb in-sample) and Cyfluthrin (125 ppb in-sample) are below their respective ALs of 700 ppb and 2000 ppb in-sample. The only Category I pesticide that does not have an AMR covering 100 ppb in-sample is Chlorfenapyr; the lowest calibrator concentration utilized for Chlorfenapyr was validated at 3.125 ng/mL in-vial, corresponding to 125 ppb in-sample. If necessary, a separate analysis using a method specific to Chlorfenapyr could be performed when it is detected at concentrations near the AL.

The primary AMRs for each of the 75 components were established upon meeting recovery and precision (%CV) criteria of 80–120% and ≤ 15%, respectively (Supplementary Tables 13 and 14). Assessment of recovery was performed using 7 independent spike-and-recoveries performed in hemp oil at up to 16 concentration levels across the primary AMR. For all 75 components, recoveries across the AMR at each concentration level met the criteria of 80–120% (n = 7) (Fig. 2B and Supplementary Table 13), with an average recovery (n = 75) of 101.9%. The minimum recovery observed was for Naled (88.1%), and the highest average recovery observed was for Propoxur (117.4%). 62 of the 75 components (82.7%) had average recoveries between 90 and 110% across their primary AMR (Supplementary Table 15).

Precision (%CV) across the primary AMR was determined similarly (n = 7) at each calibration level (Fig. 2C and Supplementary Table 14). %CV for all 75 analytes at each level within the primary and extended AMR was ≤ 15%. The average %CV across the primary AMR for all 75 analytes was 2.7%. 43 components had an average %CV of ≤ 2.5% across their respective AMRs, while 4 components had an average %CV ≥ 5% (Captan: 6.8%, Chlorfenapyr: 6.3%, Methyl Parathion: 5.3%, and Spiromesifen: 5.8%) (Supplementary Table 15).

Extension of the primary AMRs was achieved by performing independent spike and recoveries (n = 7) and reducing the amount of sample on-column by tenfold. Evaluation of accuracy and precision at 500 ng/mL in-vial (20 parts per million [ppm] in-sample) revealed that 62 components recovered within 80–120% with %CVs of ≤ 15%, thereby extending the AMRs for these analytes to 20 ppm in-sample (Fig. 2 and Supplementary Table 10). 13 components did not meet the criteria of 80–120% recovery or %CV of ≤ 15% and were limited to the primary AMRs.

With the exception of Captan, which has a well-characterized rapid degradation profile [49, 50], LLOQs were established by assessments of recovery and precision at the lowest calibration level. The LLOQs across all 75 components ranged from 0.217 to 250 ppb in-sample (0.0054–6.25 ng/mL in-vial). Out of the LLOQs validated for the 75 components, 60 (80%) were ≤ 10 ppb in-sample (0.25 ng/mL in-vial), 50 (67%) were ≤ 5 ppb in-sample (0.125 ng/mL in-vial), and 20 (27%) were ≤ 1 ppb in-sample (0.025 ng/mL in-vial). The LLOQ for Captan was established at 350 ppb in-sample, with a mean recovery 103.4% and a %CV of 7.1%. Notably, Captan at 175 ppb in-sample recovered at 85.7% with a %CV of 17.6%.

### Evaluation of carryover, ion ratios, and retention time stability

Carryover for each of the 75 components was evaluated at pesticide concentrations of 8 ppm in-sample (200 ng/mL in-vial) (Supplementary Table 16). Acceptable carryover was considered as ≤ 20% of the LLOQ for each component following two injections of pesticides at 8 ppm in-sample, corresponding to a concentration of at least twofold above the primary AMR. Observed carryover is ≤ 10% of the LLOQ following an 8 ppm injection for all components, with Propoxur having the highest carryover of 9.9% relative to its LLOQ (3.91 ppb in-sample, 156.4 in-vial).

Ion ratio tolerances were established using the 7 spike-and-recoveries performed at each level assessed within the primary AMR to determine %CVs of the ion ratios for each component (Supplementary Table 17). Tolerances were determined based on %CVs; components with ion ratio %CVs < 6.7% were required to fall within ± 20% of the average calibrator ion ratios, while components with ion ratios ≥ 6.7% (%CV*3 ≥ 20%) were required to fall within ± 30%. %CVs of ion ratios ranged from 0.4 to 14% across all 75 components. 67 components had ion ratio %CVs ≤ 5.9% and 8 components had ion ratio %CVs greater than 6.7% (Avermectin B1a, 9.1%; Avermectin B1b, 13.6%; Azoxystrobin, 10.2%; Bifenthrin, 11.8%; Chlorfenapyr, 9.1%; Cypermethrin, 11.3%; Jasmolin I, 14%; and Trifloxystrobin, 11.3%). Ion ratio monitoring was performed for all reported measurements (4 batches performed over a span of 7 weeks).

Expected retention times (RTs) and relative retention times (RRTs) to each component’s IS are shown in Supplementary Table 9. Expected RTs and RRTs were established using the average of the 7 spike-and-recoveries performed at each level assessed within the AMR. Relative retention times for all 75 analyte components were stable and fell within ± 1% of the average retention time.

### Evaluation of matrix effects

Matrix effects (ME) for all 75 components were evaluated at 500, 1000, 2000, and 4000 ppb in-sample (12.5, 25, 50, and 100 ng/mL in-vial) in hemp oil by comparing the peak areas of pesticides spiked into solvent vs pesticides spiked into extracted matrix (Supplementary Table 18). The majority of components demonstrated minimal matrix effects (80–120%) with the exception of the following 9 components: Acequinocyl (155.7% ME), Bifenthrin (Average ME: 198.5%), Captan (134.8% ME), Cyfluthrin (Average ME: 160%), Cypermethrin (Average ME: 164.4%), Etofenprox (Average ME: 128.6%), Jasmolin I (Average ME: 125.0%), (E)-Permethrin (Average ME: 151.0%), and (Z)-Permethrin (Average ME: 143.3%). 4 components were only evaluated at 50 ng/mL as they exhibited poor stability in either solvent (Acequinocyl, Captan, and Phosmet) or extracted matrix (Bifenazate) as illustrated in Supplementary Fig. 2. Notably, matrix effects for these analytes were compensated for by the SIL-IS or surrogate IS, with all 9 analytes having SIL Matrix Effects of ≤ 15% (Supplementary Table 19).

### Evaluation of the accuracy and precision at the lowest CA AL

**Fig. 3:**
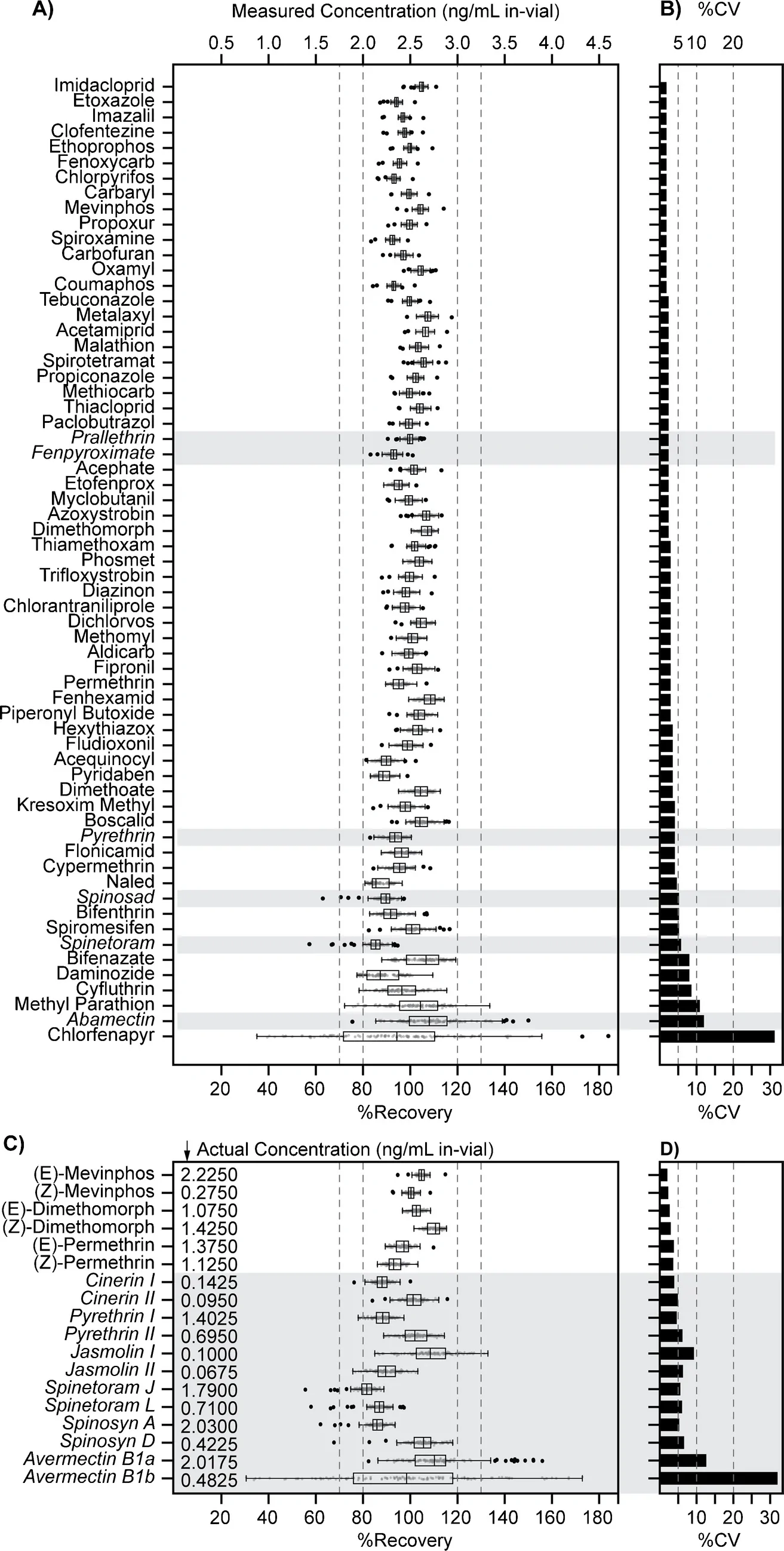
Evaluation of the Accuracy and Precision at 100 ppb in-sample (2.5 ng/mL in-vial). **A** Accuracy of spike-and-recovery at 100 ppb in-sample (2.5 ng/mL in-vial). Box-and-whisker plots illustrate the % recoveries per analyte (boxes = IQR, center line = median, whiskers = 1.5 × IQR). Replicate measurements overlaid as translucent grey dots; outliers (Tukey’s criterion) are shown as solid black circles. Dashed vertical lines mark ± 20% and ± 30% recovery from nominal values. Bottom x-axis: % recovery; top x-axis: measured concentration. Analytes quantified with surrogate IS (instead of SIL-IS) are shaded grey with italicized names. **B** Precision of spike-recovery at 100 ppb in-sample (2.5 ng/mL in-vial). Bars show %CV per analyte; dashed lines mark 5%, 10%, and 20% CV. For multi-component pesticides, measurements are summed and evaluated against the nominal total concentration (2.5 ng/mL in-vial). **C** Accuracy and **D** precision for individual components of multi-component pesticides, with actual spike concentrations are noted on the y-axis

Evaluation of accuracy and precision for all pesticides, with the exception of Captan, was performed using 120 independent spike-and-recoveries performed in hemp oil across three independent batches at the lowest AL prescribed in the CA DCC Text of Regulations (100 ppb in-sample, 2.5 ng/mL in-vial) (Fig. 3 and Supplementary Table 15). The average recoveries at 100 ppb in-sample for 63 pesticides were all within 80–120% (Fig. 3A). %CVs for all pesticides were ≤ 10% across all 120 spike-and-recoveries with the exception of Methyl Parathion (11.0%), Abamectin (12.1%), and Chlorfenapyr (31.0%) (Fig. 3B). The accuracy and precision of the 18 components that comprise the multi-component pesticides are individually plotted (Fig. 3C and D). These 18 components recovered within 80–120% (Fig. 3C) with %CVs ≤ 10%, except for Avermectin B1a (%CV = 12.5%) and Avermectin B1b (%CV = 31.8%). Notably, Avermectin B1b is a minor component of Abamectin, and its component concentration in these AL spikes was at 19.3 ppb in-sample (483 pg/mL in-vial).

### Evaluation of measurement uncertainty across the AMR and at the lowest CA AL

**Fig. 4:**
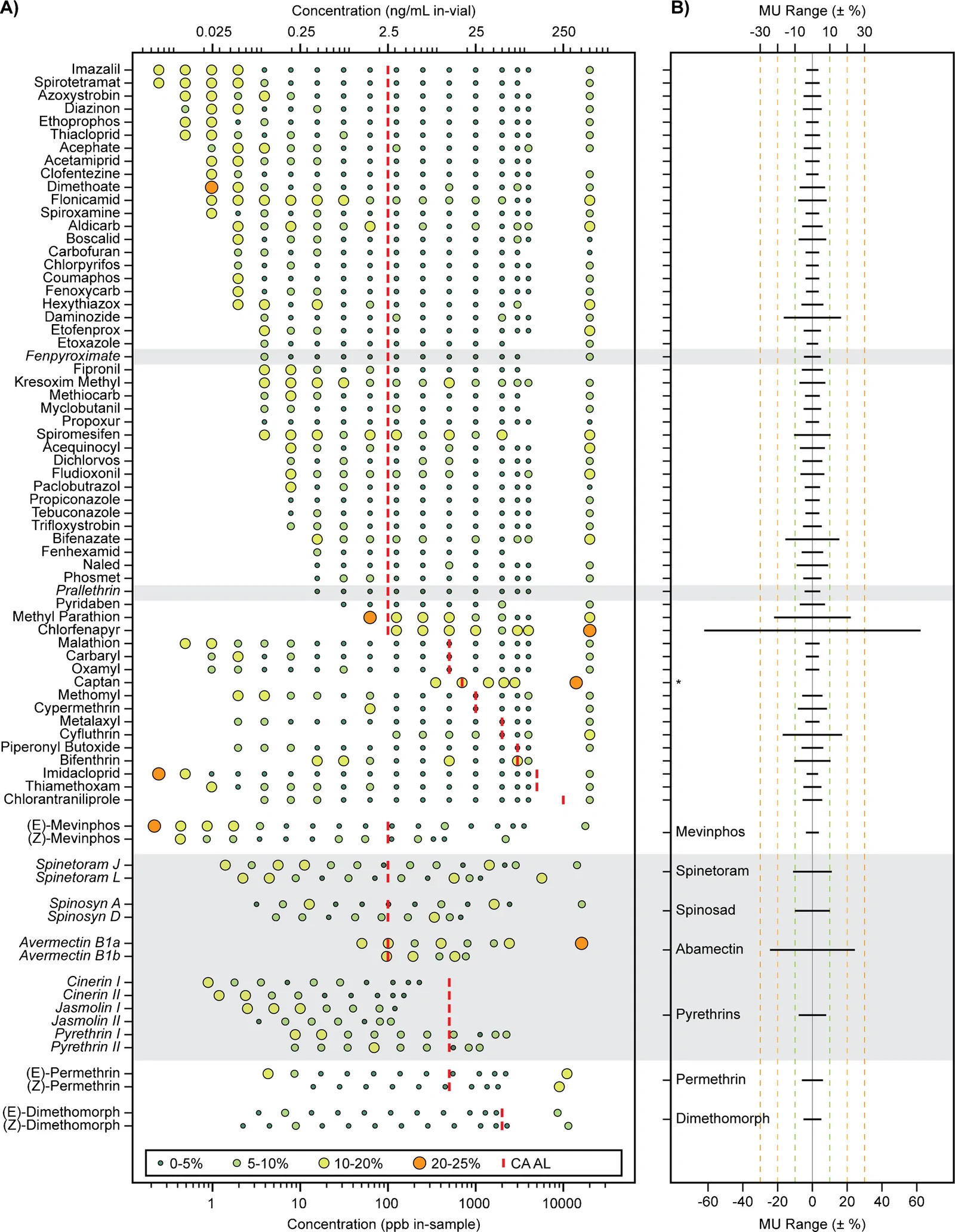
Expanded analytical measurement uncertainty (MU) across the AMR and at 100 ppb in-sample (2.5 ng/mL in-vial). **A** Expanded measurement uncertainty (MU) across the AMR. MUs are binned into four categories: 0–5%, 5–10%, 10–20%, 20–25%, represented by markers of increasing size and color. Top x-axis: in-vial concentration (ng/mL); bottom x-axis: in-sample concentration (ppb). Red vertical markers indicate California ALs. Analytes quantified with surrogate IS are italicized and shaded grey. **B** Expanded MU at 100 ppb in-sample (2.5 ng/mL in-vial). Horizontal lines span twice the expanded MUs. MUs for multi-component pesticides are reported as the MUs of the sum of individual component quantifications. Dashed lines mark ± 5, ± 10, ± 20, and ± 30% expanded MU. *Captan was not evaluated at 100 ppb in-sample

Expanded combined analytical measurement uncertainties (MUs) were estimated according to CLSI EP29-A (31) and evaluated across the primary AMRs (Fig. 4A and Supplementary Table 20). Average MUs across 75 components ranged from (± 2.3–± 14.6%), with an average MU (n = 75) of 5.7% for all 75 components. The lowest average MU was for Propiconazole (± 2.3%) and the highest average MU was for Captan (± 14.6%); 38 components (50.6%) had average MUs within ± 5%. MUs across the LLOQs for all 75 components ranged from ± 2.1% to ± 22.7%. The lowest MU observed across all LLOQs for all 75 components was ± 2.1% for (E)-Dimethomorph (LLOQ: 3.4 ppb in-sample; 0.084 ng/mL in-vial), while the largest was ± 22.7% for Methyl Parathion (LLOQ: 62.5 ppb; 1.56 ng/mL in-vial). At the LLOQ, 50 of 75 components (66%) had MUs ± 15%, and the average MU at the LLOQ for all 75 components was 11.5%.

Across all 75 components, 856 unique MUs were determined at discrete concentrations within the primary AMR. All 856 MUs were within ± 25%, 852 MUs (99.5%) were within ± 20%, 736 MUs (86.0%) were within ± 10%, and 537 MUs (62.7%) were within ± 5%. At the upper limits of the 62 extended AMRs, MUs ranged from ± 9.2 to ± 24.2%, and all components had MUs within ± 20.0%, with the exception of Avermectin B1a (23.3%), Chlorfenapyr (23.9%), and Captan (24.2%). Expanded combined MUs for the 120 AL spikes were within ± 15.0% for all pesticides (n = 63) (Fig. 4B), with the exceptions of Bifenazate (15.5%), Daminozide (16.5%), Cyfluthrin (17.1%), Methyl Parathion (22.0%), Abamectin (24.4%), and Chlorfenapyr (62.2%). Of these 6 pesticides, Abamectin lacks a commercially available stable-isotope analog, which likely contributed to the observed high MU. Methyl Parathion and Chlorfenapyr have been shown to perform robustly on GC-MS assays [24, 26, 36].

### Systematic benchmarking defines the limits of surrogate internal-standard correction

**Fig. 5:**
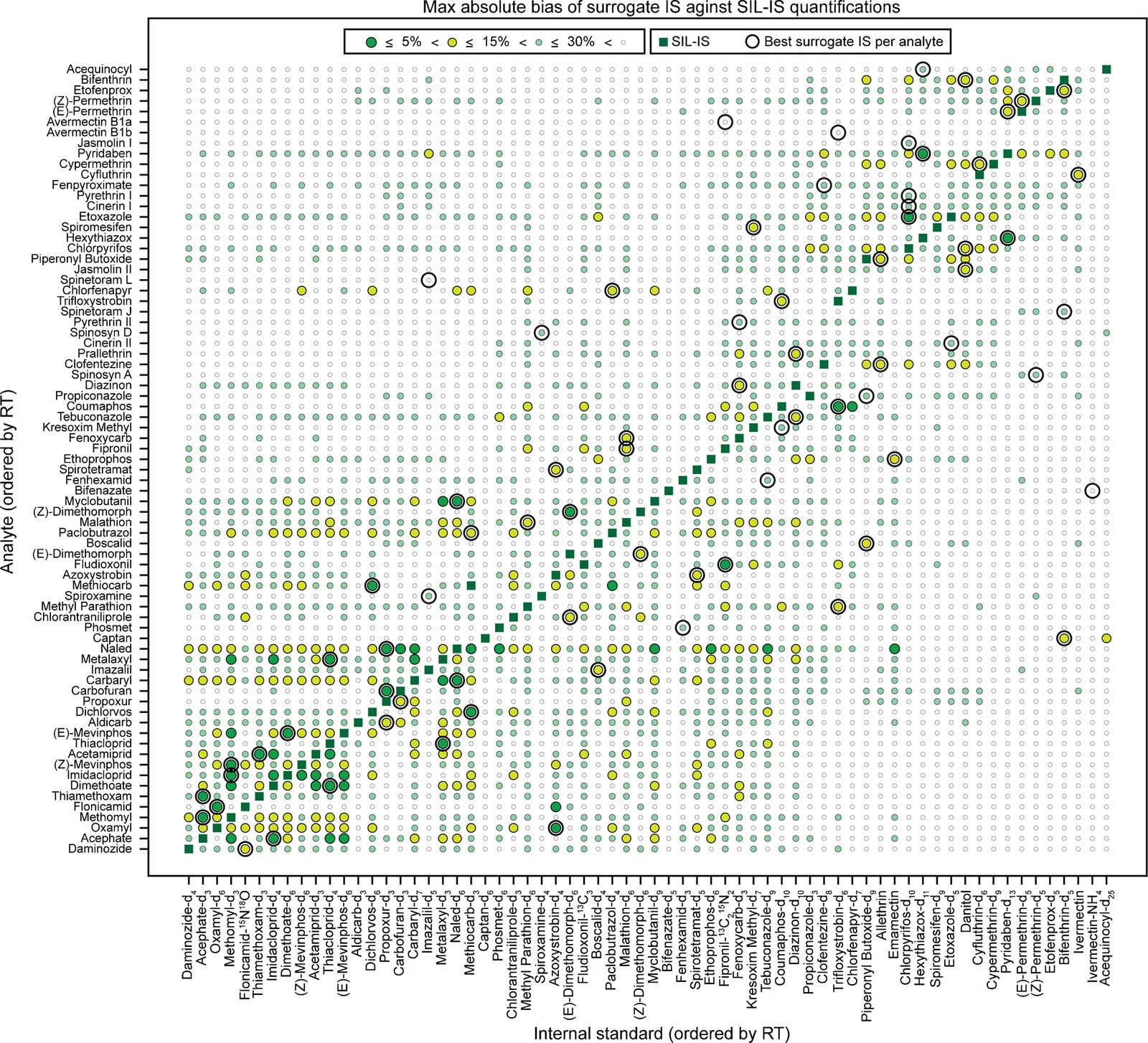
Internal standard (IS)-analyte performance ordered by retention time. IS-analyte pairs are displayed on a uniformly spaced grid in which both axes are categorical, with the analyte (y-axis) and IS (x-axis) ordered by increasing chromatographic retention time (RT). Each point represents an IS-analyte pair, with marker color and size encoding the max absolute percent bias (MAB) using four discrete bins: ≤ 5%, ≤ 15%, ≤ 30%, and > 30% (darker and larger markers indicate lower bias). Pairs with analog SIL-IS are indicated by a green square marker and provide optimal accuracy (MAB = 0). For each analyte, the best-performing surrogate IS, defined as the component with the lowest MAB, is highlighted with a hollow circular outline

The applicability of surrogate ISs was systematically evaluated by assessing calibration accuracy (see Methods). This analysis incorporated data from both the 17 × 7 spike-and-recovery experiments and the 120 AL spikes. Linear regression was carried out using calibrators prepared and analyzed across four independent analytical batches, resulting in 4950 unique component-IS regression models and a total of 19,800 (4950 × 4) individual linear regression fits (Supplementary Table 21). These curves were subsequently used to assess accuracy from the spike-and-recoveries performed across the AMR and at 100 ppb. Biases between calculated concentrations derived from either component-SIL-IS pairs or component-surrogate IS pairs were used to assess the applicability of the respective surrogate ISs. The nominal spiked-in concentrations were used when an SIL-IS was unavailable. After filtering to include only quantifier ion measurements and quantifications within the primary AMR of each component, a total of 1,030,787 individual bias values were computed and reported (Fig. 5). These data were summarized as a heatmap showing the maximum absolute bias (MAB) observed for each of the 4950 component-IS pairs (Fig. 5 and Supplementary Table 11). On average, 207 individual measurements were used to determine the MAB for each component-IS pair. Captan was excluded from the AL spike-and-recovery evaluation at 100 ppb and was only assessed using the 17 × 7 spike-and-recovery data (n = 39 points).

Evaluation of all component-IS pairings demonstrated substantial variability in surrogate IS performance across all components. Of the 66 IS components evaluated, 32 (48.5%) demonstrated a MAB of ≤ 5% for at least one component when used as a surrogate IS. Among these, 5 components demonstrated broader applicability as surrogate ISs, generating a MAB of ≤ 5% for three or more components: Methomyl-d_3_ (6 components), Imidacloprid-d_4_ and Thiacloprid-d_4_ (4 components), and Metalaxyl-d_3_ and (E)-Mevinphos-d_6_ (3 components). When a more permissive performance threshold was applied, 59 of the 66 IS components (89.4%) generated a MAB of ≤ 15% for at least one component when used as a surrogate IS. Of these, 34 IS components (51.5%) demonstrated a MAB of ≤ 15% for at least 5 components. Thiacloprid-d_4_ provided the greatest overall surrogate IS coverage, generating a MAB of ≤ 15% for 12 components: Acephate (4.7%), Acetamiprid (4.7%), Carbaryl (6.5%), Imidacloprid (2.1%), Malathion (11.3%), Metalaxyl (2.1%), Methomyl (9.9%), (E)-Mevinphos (8.1%), Myclobutanil (10.2%), Naled (6.8%), Oxamyl (13.8%), and Paclobutrazol (14.9%).

Six pesticides, comprising 14 individual components, were quantified without isotope-dilution due to a lack of generally available SIL analogs: Abamectin (Avermectin B1a and Avermectin B1b), Prallethrin, Fenpyroximate, Pyrethrins (Pyrethrin I, Pyrethrin II, Cinerin I, Cinerin II, Jasmolin I, Jasmolin II), Spinosad (Spinosyn A and Spinosyn D), and Spinetoram (Spinetoram J and Spinetoram L). For Prallethrin, 12 surrogate ISs generated maximum MABs of ≤ 20%, with the five lowest observed MABs obtained using Diazinon-d_10_ (11.6%), Fenoxycarb-d_3_ (13.4%), Clofentezine-d_8_ (15.3%), Allethrin (15.9%), and Coumaphos-d_10_ (16.3%). For Fenpyroximate, 6 surrogate ISs generated MABs of ≤ 20%, with the best-performing ISs being Clofentezine-d_8_ (15.4%), (E)-Permethrin-d_5_ (15.4%), Imazalil-d_5_ (17.5%), Etoxazole-d_5_ (18.6%), and Danitol (19.3%). For individual pyrethrin components, surrogate ISs generating MABs of ≤ 20% were identified as listed in Supplementary Table 22.

Surrogate ISs generating MABs of ≤ 30% were identified for Jasmolin I (Chlorpyrifos-d_10_ [29.1%]), Spinetoram J (Bifenthrin-d_5_ [27.3%], (E)-Permethrin-d_5_ [29.5%], (Z)-Permethrin-d_5_ [28.0%]), Spinosyn A (Bifenthrin-d_5_ [27.3%], (E)-Permethrin-d_5_ [27.4%], (Z)-Permethrin-d_5_ [25.4%]), and Spinosyn D (Acequinocyl-d_25_ [29.6%], Spiroxamine-d_4_ [29.1%]). No surrogate ISs evaluated demonstrated a MAB of ≤ 30% for either Avermectin B1a or Avermectin B1b. The lowest observed MABs for Avermectin B1a were obtained using Fipronil-^13^C₂^15^N₂ (55.1%), Ivermectin-NH₄⁺ (58.2%), Acephate-d_3_ (58.7%), and Emamectin (60.5%).

High applicability of surrogate ISs was found to be inversely associated with chromatographic retention time, with 9.8-fold more component-IS pairs exhibiting MABs ≤ 5% eluting in the first half of the gradient (RT ≤ 12 min; 49 vs 5) compared to the second half (RT 12–25 min). This association persisted for MAB thresholds of ≤ 10% (3.9-fold; 136 vs 35) and ≤ 15% (2.4-fold; 239 vs 99).

Across the panel, bias was associated with the surrogate IS’s chemical similarity and chromatographic elution profile relative to the target analyte. Surrogates with similar retention times and structural features generally produced lower bias components across their established primary AMR, as indicated by clusters of analyte-IS pairs with MABs ≤ 5% observed along the diagonal of the heatmap (Fig. 5). This diagonal trend observed in the heatmap indicates that surrogates with retention times near those of the target analytes tend to yield lower biases, supporting the use of chromatographically matched surrogates when SIL analogs are unavailable.

A subset of analyte-surrogate combinations achieved ≤ 5% MAB (1.1%; 54 of 4889), indicating near-equivalent performance to the matched SIL-IS, while a larger fraction (6.6%; 322 of 4889) fell within ≤ 15% MAB. However, numerous pairings exceeded 30% MAB (68.9%; 3369 of 4889), demonstrating that an inappropriately matched surrogate IS can introduce substantial bias. This analysis illustrates that while isotope-dilution mass spectrometry using SIL analogs provides robust quantification, carefully selected surrogate ISs, particularly those with similar physicochemical properties, can provide accurate quantification for the majority of components in the panel. Nevertheless, for compounds like Avermectin B1a and B1b, a SIL analog might be necessary, as indicated by the observed poor performances among surrogates.

### Replicate analysis to reach target measurement uncertainty

**Fig. 6:**
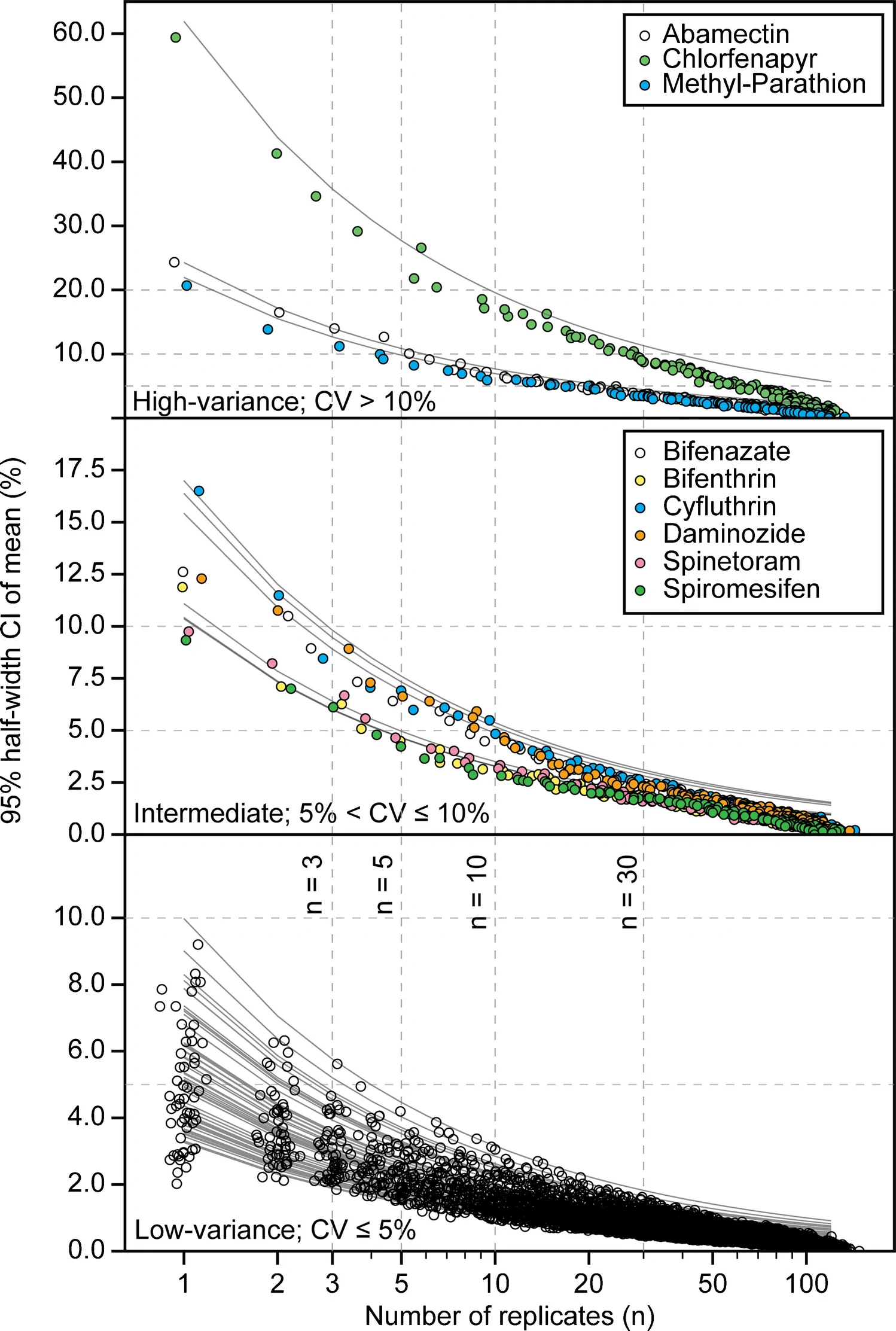
Empirical and model-based estimation of measurement uncertainty for pesticide spike-recovery measurements. Empirical uncertainty values are shown as scatter points. Model predictions are shown as smooth curves overlaid on the empirically sampled data. Plots are stratified by analyte precision into three panels: high-variance (%CV > 10%), intermediate-variance (5% < %CV ≤ 10%), and low-variance pesticides (%CV ≤ 5%). The x-axis shows the number of replicate measurements (n) on a logarithmic scale. The y-axis shows the half-width of the 95% confidence interval of the mean. Horizontal dashed lines indicate practical uncertainty thresholds (5%, 10%, and 20%), and vertical dotted lines indicate common replicate counts (n = 3, 5, 10, and 30)

The 120 spike-and-recoveries at 100 ppb were used to estimate the sample size needed to reach MUs ≤ 20% at the lowest CA AL. MU was modeled and empirically sampled to simulate how analytical variability influences the interpretability of measured concentrations near regulatory compliance limits. Assuming normal error distributions and that the observed MUs were sufficiently close to the intrinsic MUs, a %CV-based MU model could be constructed with an expected $1/\sqrt{n}$ scaling of uncertainty in the mean of repeated measurements. This resulted in 63 MU models using the %CVs calculated from the 120 AL spikes (Fig. 6 and Supplementary Table 23). To simulate the number of replicate measurements required for a stable estimate of recovery, sampled means were calculated for replicate counts at n = 1–120. At each n, the empirical distribution of the sampled mean was summarized by its 95% confidence interval (CI), and MU was expressed as the % half-width of that CI. The MUs at each n, calculated from the sample means, are then plotted alongside the %CV-based MU models.

The number of replicates required to estimate means within ± 5, 10, and 20% of the “true mean” (n = 120) scaled with analyte %CV, ranging from as few as a single replicate for low-variance compounds to over 30 replicates for high-variance analytes (Table 1). Each pesticide was categorized as high, intermediate, or low-variance based on their %CVs. For all three groups, MU decreased predictably with increasing replicate measurements (Fig. 6 and Supplementary Table 23). For measurements performed in duplicate (n = 2), uncertainty in the mean concentration was within ± 20% at the 95% CI for all pesticides except Chlorfenapyr (41.3%). For measurements performed in triplicate (n = 3), 3 high-variance pesticides exceeded ± 10% at the 95% CI (Abamectin [14.0%], Methyl Parathion [11.2%], and Chlorfenapyr [34.6%]). These 3 pesticides exhibited the largest 95% CIs, exceeding 20% MU at n = 1 and remaining above 10% until additional replicates were performed: 4 for Methyl Parathion, 6 for Abamectin, and 27 for Chlorfenapyr. 6 intermediate-variance pesticides (5% < %CV ≤ 10%) demonstrated moderate uncertainty of the mean, with 95%CI half-widths of approximately 8–18% at n = 1 that declined below 5% by roughly n = 5–12. In contrast, the remaining 54 pesticides were observed to have low variance (%CV ≤ 5%), showing narrow uncertainty in the mean concentration across all replicate counts, generally achieving ≤ 5% uncertainty by n = 3 and approaching ≤ 2% by n = 20.

**Table 1:** Number of replicate measurements needed to obtain a mean within thresholds of ± 5%, ± 10%, and ± 20% of the true mean with 95% confidence

Analyte	%CV	5%	10%	20%
Chlorfenapyr	30.96	64	27	8
Abamectin	12.14	18	6	2
Methyl Parathion	10.98	17	4	2
Cyfluthrin	8.50	10	3	1
Daminozide	8.19	10	3	1
Bifenazate	7.72	9	3	1
Bifenthrin	5.18	5	2	1
Spinetoram	5.55	5	1	1
Spiromesifen	5.21	4	1	1
Spinosad	4.99	4	1	1
Naled	4.51	3	1	1
Cypermethrin	4.15	3	1	1
Flonicamid	4.06	3	1	1
Pyrethrins	3.94	3	1	1
Boscalid	3.94	3	1	1
Kresoxim Methyl	3.68	3	1	1
Dimethoate	3.64	2	1	1
Pyridaben	3.61	2	1	1
Acequinocyl	3.55	2	1	1
Fludioxonil	3.41	2	1	1
Hexythiazox	3.13	2	1	1
Piperonyl Butoxide	3.10	2	1	1
Fenhexamid	3.08	2	1	1
Permethrin	3.01	2	1	1
Fipronil	3.00	2	1	1
Aldicarb	2.92	2	1	1
Methomyl	2.90	2	1	1
Acetamiprid	2.05	2	1	1
Phosmet	2.61	2	1	1
Myclobutanil	2.50	2	1	1
Dichlorvos	2.83	1	1	1
Chlorantraniliprole	2.79	1	1	1
Diazinon	2.68	1	1	1
Trifloxystrobin	2.67	1	1	1
Thiamethoxam	2.58	1	1	1
Dimethomorph	2.54	1	1	1
Azoxystrobin	2.51	1	1	1
Etofenprox	2.47	1	1	1
Acephate	2.46	1	1	1
Fenpyroximate	2.41	1	1	1
Prallethrin	2.24	1	1	1
Paclobutrazol	2.18	1	1	1
Thiacloprid	2.18	1	1	1
Methiocarb	2.12	1	1	1
Propiconazole	2.07	1	1	1
Spirotetramat	2.06	1	1	1
Malathion	2.05	1	1	1
Metalaxyl	2.03	1	1	1
Tebuconazole	1.98	1	1	1
Coumaphos	1.97	1	1	1
Oxamyl	1.95	1	1	1
Carbofuran	1.95	1	1	1
Spiroxamine	1.94	1	1	1
Propoxur	1.89	1	1	1
Mevinphos	1.84	1	1	1
Carbaryl	1.83	1	1	1
Chlorpyrifos	1.82	1	1	1
Fenoxycarb	1.80	1	1	1
Ethoprophos	1.77	1	1	1
Clofentezine	1.76	1	1	1
Imazalil	1.72	1	1	1
Etoxazole	1.65	1	1	1
Imidacloprid	1.63	1	1	1

Across all pesticides, uncertainty declined exponentially with increasing replication, with the largest reductions occurring between n = 1 and n = 5, followed by progressively smaller gains thereafter. Increasing replication beyond approximately 30 measurements yielded only modest improvement. Collectively, these findings demonstrate that the replicate requirements to achieve practical uncertainty thresholds (e.g., 5%, 10%, or 20%) depend strongly on assay precision. The quantitative measurement of analytes with higher variance leads to a higher replicate requirement to lower uncertainty in the mean measured concentration.

## Discussion

Residual pesticide testing in cannabis-derived products is analytically demanding because quantitative determinations directly inform regulatory compliance. This study aims to address challenges presented and highlighted by prior literature. Moulins et al. developed workflows in cannabis leaves, flowers, and oil. However, poor recoveries (< 70%) were reported for several pesticides, including Diazinon (7–8%), Bifenthrin (19–49%), Chlorpyrifos (36–61%), Cyfluthrin (29–52%), and Cypermethrin (32–38%) [24]. More recently, MacKenzie et al. emphasized the matrix complexity of cannabis dried inflorescence and validated a method using matrix-matched calibration [26]. However, the published methods used SIL-IS only to a limited extent, which may not sufficiently compensate for matrix effects for pesticides lacking matched internal standards. MacKenzie et al. also showed that this limitation is further exacerbated by the heterogeneity of cannabis products, in which subtle but significant differences in composition across cultivars give rise to highly variable recoveries due to analyte-specific matrix effects. In contrast, this study shows that extensive use of isotope dilution greatly improves the accuracy and precision of multiplex quantification across pesticides with widely differing physicochemical behavior and compensates for matrix effects in highly lipophilic cannabis-derived oil. Moreover, this study provides a framework for identifying acceptable surrogate internal standards when matched SIL-IS are not readily available.

In the United States, cannabis remains classified as a Schedule I controlled substance by the Drug Enforcement Administration, resulting in a decentralized regulatory framework in which individual states independently establish pesticide panels, ALs, and testing requirements [2, 8, 51–71]. This regulatory fragmentation has led to substantial variability in permissible pesticide concentrations and analytical performance expectations, limiting harmonization of testing methodologies across laboratories and increasing reliance on individual laboratory expertise to maintain analytical robustness and the reliability of reported results. These regulatory realities elevate the importance of analytical methods that provide not only sufficient sensitivity at compliance thresholds but also robust quantitative performance for physicochemically diverse pesticides across a variety of cannabis-derived matrices.

Several US states, including Colorado, New York, and Washington, have established quantitative pesticide ALs defined as maximum allowable concentrations (e.g., µg/g), which serve as regulatory decision thresholds for determining product compliance [64, 72, 73]. In California, the DCC has established ALs for 66 pesticides and uniquely defines differential limits based on two regulatory product categories: inhalable and non-inhalable products [4]. While regulatory classification is binary, these categories encompass a broad spectrum of cannabis-derived matrices, including plant material, concentrated extracts, distillates, oils, infused beverages, and other manufactured products. These matrices differ substantially in chemical composition and physicochemical properties, introducing matrix-dependent analytical variability that complicates accurate and reproducible quantification of pesticide residues across product types. Variability in co-extracted constituents such as cannabinoids, terpenes, flavonoids, and triglycerides can affect analyte extraction recovery and analytical measurements, underscoring the importance of analytical methods that compensate for matrix effects [11].

In contrast to the state-specific regulatory framework in the United States, Canada regulates cannabis federally and maintains a list of 96 pesticides with defined limits of quantification (regulatory equivalent to CA DCC AL, referred to as AL from here on) for fresh cannabis, dried cannabis, and cannabis oil [6, 7]. Of the 64 pesticides evaluated in this method, 62 are also included in Canada’s regulated pesticide panel, reflecting substantial overlap in pesticides subject to regulatory monitoring across jurisdictions. Canadian regulations also define matrix-specific ALs and explicitly recognize cannabis oil as a distinct analytical matrix due to its unique physicochemical properties relative to plant material. These matrix-specific thresholds reflect differences in achievable analytical sensitivity and precision across product types. In contrast, uniform ALs applied across chemically diverse cannabis-derived matrices place an increased burden on the development of analytical methods capable of maintaining accuracy and precision across matrices with differing physicochemical properties and polarities. This regulatory and analytical overlap highlights the broader applicability of analytical methods designed to meet stringent performance requirements in oil matrices, which are among the more analytically challenging cannabis-derived product types.

The multiplex LC-MS/MS method described here was developed primarily to address the analytical challenges faced by laboratories seeking to maintain compliance with the CA DCC regulatory framework. However, the extensive chromatographic optimization, implementation of isotope dilution using SIL-IS, and structured characterization of measurement uncertainty provide a broader analytical foundation for residual pesticide testing across diverse regulatory environments. This assay enables simultaneous quantification of 64 pesticides (75 components) specified in the DCC Text of Regulations (2026) within a cannabis-derived oil matrix at concentrations relevant to regulatory decision thresholds. Of these 64 pesticides, 62 are also included in Canada’s federally regulated pesticide panel. Notably, only five of these overlapping pesticides (Chlorfenapyr, Methyl Parathion, Bifenazate, Phosmet, and Pyridaben) did not meet the lowest LOQs defined across all Canadian product types. However, the LLOQ for Chlorfenapyr in this method (125 ppb in hemp oil) remains well below Canada’s LOQ requirement for cannabis oil (1500 ppb), while Canada does not define LOQs for Methyl Parathion or Phosmet in cannabis oil, reflecting known analytical challenges associated with these compounds in oil matrices. For Bifenazate and Pyridaben, in-sample LLOQs of 16 ppb and 31 ppb (0.39 and 0.78 ng/mL in-vial), respectively, were established based on predefined acceptance criteria designed to preserve an extended AMR encompassing California’s lowest regulatory AL (100 ppb) while supporting accurate quantification of highly contaminated specimens. Although lower LLOQs were technically achievable, doing so required lowering the upper limit of the AMR, thereby limiting reliable quantification at elevated concentrations. This illustrates the inherent trade-off between analytical sensitivity and the dynamic range required to ensure accurate quantification at regulatory decision thresholds and for heavily contaminated samples.

A defining characteristic of this assay is its ability to simultaneously quantify chemically diverse pesticides. Based on experimentally determined octanol-water partition coefficients (LogP), this method quantitatively measures pesticides with LogP values ranging from − 1.5 to 6.9, representing an 8.4-unit span corresponding to over a 250-million-fold difference in polarity (Supplementary Table 9). Despite this physicochemical diversity, LLOQs were ≤ 10 ppb in-sample for the majority of analytes, while upper limits of the AMR extended to as high as 20,000 ppb in-sample for most analytes. Most analytes exhibited AMRs spanning three to four orders of magnitude, ensuring that regulatory decision thresholds were positioned well within validated regions of assay performance while preserving sufficient dynamic range to quantify highly contaminated specimens. The chromatographic gradient was deliberately structured to distribute analytes across the chromatographic run, minimizing co-elution of compounds with substantially different ionization efficiencies and reducing transition competition within the mass spectrometer. This chromatographic architecture supported stable quantitative performance across analytes exhibiting wide variation in retention time, polarity, and ionization efficiency. Additionally, extending the AMR to 20,000 ppb in-sample for most analytes through reduced injection volume provides operational flexibility for quantifying highly contaminated samples without requiring additional dilution.

SIL-IS played a central role in maintaining quantitative accuracy and precision across diverse analytes. Because SIL-ISs possess nearly identical physicochemical properties to their non-labeled analogs, they experience equivalent recovery losses during extraction and analytical behavior during LC-MS/MS analysis. In principle, matrix effects and extraction variability can be addressed using standard addition; however, standard addition requires preparation and analysis of multiple levels of fortified replicates for each specimen, substantially increasing analytical workload, reducing throughput, and introducing additional opportunities for preparation-related variability. In contrast, isotope dilution using SIL-ISs provides an analogous correction mechanism within a single measurement. Monitoring IS recovery enables sample-specific assessment of extraction efficiency and analytical stability, allowing matrix effects and variability to be detected and compensated without requiring replicate analysis. This approach preserves the accuracy benefits of matrix-matched correction while providing the efficiency and scalability required for routine regulatory testing. The extensive incorporation of SIL-ISs therefore represents a critical analytical safeguard against matrix-induced bias and supports reliable multiplex quantification in chemically complex cannabis-derived matrices.

Moreover, the incorporation of SIL-IS enables implementation of an exception-based review framework that alleviates the significant data-review burden for large panels of pesticides. For example, a batch of 40 samples in this study produces over 9000 chromatograms (quantifier, qualifier, and IS ions), rising to more than 15,000 once calibrators and quality control samples are included, all of which would require extensive manual review without SIL-ISs. However, since most analytes have a matched SIL-IS, data review can be semi-automated through predefined acceptance criteria for IS retention time (e.g., ± 0.2 min) and recovery (e.g., 25–175% relative to calibrators). These criteria qualify chromatographic performance and flag unexpected matrix effects, extraction variability, or analytical instability, so that only samples failing these thresholds require detailed manual evaluation. Because 58 of the 64 pesticides are quantified by isotope dilution, additional confirmatory strategies such as standard addition are needed only for the small subset of analytes lacking matched SIL analogs. Isotope dilution also compensates for analyte instability and recovery losses during sample preparation and extended runs, as shown for Bifenazate, which degraded rapidly in the extracted matrix (Supplementary Fig. 2). Given the approximately 40-min cycle time and multi-day durations of large batches, SIL-IS preserves quantitative accuracy despite time-dependent changes in analyte response.

Systematic benchmarking of surrogate IS further demonstrated that only a small fraction of surrogate pairings achieved quantitative performance comparable to matched SIL-ISs. Surrogate ISs with dissimilar chromatographic retention profiles exhibited a reduced ability to compensate for matrix effects, particularly for highly lipophilic analytes that elute later in the chromatographic gradient. These findings extend beyond this study by providing a generalizable analytical framework for IS selection in complex multi-residue LC-MS/MS methods. These findings reinforce the importance of chromatographic co-elution and physicochemical similarity between analyte and IS for robust quantification.

Structured characterization of analytical MU demonstrated that the benefits of extensive isotope dilution were not limited to recovery but also extended to precision at regulatory decision thresholds. Analytical MU, estimated in accordance with CLSI EP29-A, remained within approximately ± 15% for most analytes at 100 ppb in-sample. Moreover, a comparison of modeled and empirically sampled data using 120 replicate measurements confirmed the convergence of uncertainty estimates and demonstrated that analytical variability was well characterized. These findings provide a metrologically grounded framework for interpreting measured concentrations near fixed compliance limits and demonstrate that quantitative imprecision near regulatory thresholds can be systematically characterized and controlled.

It is important to note that the expanded MU described here reflects analytical variability within the laboratory workflow and does not account for additional sources of variability arising from field sampling, specimen handling, storage, transport, or heterogeneity in bulk cannabis-derived products. These pre-analytical factors may contribute additional variability beyond the analytical uncertainty quantified here and collectively define the total MU associated with reported concentrations. Accordingly, the uncertainty characterized in this study likely represents a lower bound of total variability encountered in real-world testing environments.

Certain pesticides included in multi-residue cannabis panels may also be amenable to quantification by GC-MS/MS, particularly compounds that are sufficiently volatile, thermally stable, and well-suited to electron ionization. Notably, the remaining 2 pesticides regulated by the CA DCC that are not incorporated into the described LC-MS/MS method, Pentachloronitrobenzene and Chlordane, are organochlorine compounds historically characterized with high sensitivity and specificity by GC-MS/MS. Chlorfenapyr, although included in this study, shares similar physicochemical properties and is likewise amenable to GC-based analysis. In this study, Chlorfenapyr exhibited comparatively higher analytical MU at regulatory thresholds under ESI LC-MS/MS conditions. Although replicate analysis reduces uncertainty in the mean concentration, this strategy is operationally impractical for routine compliance testing given typical batch sizes, instrument cycle times, and the associated data-review burden. Alternative ionization strategies, such as APCI, have been shown to improve robustness for less polar, thermally stable compounds that ionize poorly under ESI conditions [74]. Nevertheless, operationally transitioning from an ESI to an APCI source introduces additional operational complexity and risk in a regulated production testing environment.

Importantly, several pesticides that perform well under LC-MS/MS conditions are also amenable to GC-MS analysis, providing an opportunity for orthogonal confirmation of measured concentrations when warranted. As the majority of analytes in this panel are quantified using SIL-IS, a single initial extract can be split and used for both LC-MS/MS and GC-MS/MS analysis without requiring duplicate sample preparation. Isotope dilution compensates for variability during sample preparation and enables cross-platform comparability. For analytes exhibiting elevated uncertainty under LC-MS/MS conditions, orthogonal GC-MS/MS confirmation represents an analytically robust strategy to reduce MU near regulatory decision thresholds.

There are several limitations to this method that should be considered. First, chromatographic separation of a large multi-residue pesticide panel required a relatively long LC method, with a total run time of more than 40 min per injection. Although this chromatographic separation was intentionally designed to support robust quantification across chemically diverse analytes, the run time may limit implementation in high-throughput production settings. Future work could be optimized further to reduce analysis time while maintaining analytical performance.

Second, the method was validated using a single hemp oil matrix. Because matrix composition can vary across hemp oil sources and related cannabis-derived products, the present validation should not be assumed to establish equivalent performance in dried flower, concentrates, distillates, infused products, or other cannabis-derived matrices without additional matrix-specific verification. Evaluation of additional hemp oil lots from multiple sources and broader cannabis-derived matrix types would further clarify matrix effects and method robustness.

Third, the current LC-MRM-MS panel did not include two pesticides regulated by the CA DCC, chlordane and pentachloronitrobenzene, and several additional pesticides regulated in Canada were also outside the scope of this method. In addition, chlorfenapyr showed comparatively poor analytical performance using the present ESI-LC-MS/MS workflow. Given the extensive chromatographic separation achieved, future iterations may incorporate additional pesticides into the LC-MS/MS panel. However, highly nonpolar or ESI-unfavorable compounds, including pentachloronitrobenzene, chlordane, and chlorfenapyr, may be better suited to alternative or complementary approaches such as GC-MS/MS or LC-APCI-MS/MS. 

Fourth, the surrogate-IS benchmarking results are specific to the validated matrix, chromatographic method, instrument platform, and data-processing workflow. Although the analysis provides a systematic framework for selecting surrogate internal standards, the identified surrogate assignments should be re-evaluated and verified before transferring to other matrices, LC gradients, instruments, or laboratories.

Finally, the reported expanded measurement uncertainties reflect analytical uncertainty within the validated workflow and do not include pre-analytical sources of variability such as sampling, product heterogeneity, storage, transport, or standard-material uncertainty.

## Conclusion

Taken together, the analytical architecture demonstrated here supports accurate and precise multiplex quantification of pesticides across chemically diverse analytes in a cannabis-derived oil matrix. To our knowledge, this is the first study to utilize extensive stable isotope dilution to evaluate the applicability of surrogate ISs and validate a method for the robust quantification of this panel of 64 pesticides in hemp oil. Robust chromatographic separation, extensive SIL-IS coverage, and structured characterization of analytical MU collectively provide a rigorous analytical workflow for regulatory pesticide testing in cannabis products. These performance characteristics support the use of isotope-dilution LC-MS/MS as a reliable platform for quantitative pesticide analysis in cannabis-derived products. While the present study was validated for a specific multi-residue pesticide panel and matrix, the principles demonstrated here, benchmarking of surrogate IS applicability with SIL-IS, chromatographic resolution, and empirical evaluation of uncertainty may inform the development and validation of other multiplex LC-MS/MS assays in chemically complex matrices. These performance characteristics support the use of isotope-dilution LC-MS/MS for quantitative pesticide analysis in cannabis-derived products, where regulatory decisions depend on accurate and reproducible quantitative measurements.

## Supplementary Information

Below is the link to the electronic supplementary material.

Supplementary file1 (DOCX 447 KB)

Supplementary file2 Surrogate IS Analysis - Maximum Absolute Bias across 4 Analytical Batches (XLSX 83.7 KB)

Supplementary file3 Surrogate IS Contiguous Linear Regression Fittings (XLSX 2.57 MB)

## Data Availability

The raw data supporting this study are provided in the Supplementary Tables. All code used for data analysis is deposited at [GitHub/Zenodo: https://github.com/CMCR-Reference-Lab/Surrogate_Internal_Standard_Analysis; https://doi.org/10.5281/zenodo.19776298]. Additional data is available from the corresponding author upon request.

## Abbreviations

H_2_O: 18 MΩ·cm water
ACN: Acetonitrile
AL: Action limit
NH_4_HCOO: Ammonium formate
AMR: Analytical measurement range
MU: Analytical measurement uncertainty
AUC: Area under the curve
APCI: Atmospheric pressure chemical ionization
XLogP: Calculated octanol-water partition coefficient
R^2^: Coefficient of determination
%CV: Coefficient of variation
CI: Confidence interval
k: Coverage factor
DCC: Department of Cannabis Control
ESI: Electrospray ionization
XIC: Extracted ion chromatogram
GC-MS/MS: Gas chromatography-tandem mass spectrometry
IS: Internal standard
IQR: Interquartile range
IDMS: Isotope dilution mass spectrometry
LC-MS/MS: Liquid chromatography tandem mass spectrometry
LLOQ: Lower limit of quantification
ME: Matrix effect
MAB: Maximum absolute bias
MeOH: Methanol
MRM: Multiple reaction monitoring
LogP: Octanol-water partition coefficient
ppb: Parts per billion
CA: California
CRM: Certified reference material
ppm: Parts per million
SIL: Stable isotope-labeled
