# Supplementary material for: Enhanced quantification of 64 pesticides in cannabis-derived oil by isotope-dilution LC-MRM-MS at regulatory decision thresholds
Source: Anal Bioanal Chem. 2026 Jul 18;418(19):6357–80. doi: 10.1007/s00216-026-06661-5 (PMC13585869; doi:10.1007/s00216-026-06661-5)
Supplement: Supplementary file 1 [file 216_2026_6661_MOESM1_ESM.docx]

# Supplementary Figure and Tables

# Journal: Analytical and Bioanalytical Chemistry

# TITLE AND AUTHORS

Enhanced Quantification of 64 Pesticides in Cannabis-Derived Oil by Isotope-Dilution LC-MS/MS at Regulatory Decision Thresholds

Raymond T. Suhandynata^1,2^, Jiaxi Cai^3^, Benjamin J. Spiegel^2^, Thomas D. Marcotte^4^, Igor Grant^4^, Robert L. Fitzgerald^1^, Jeremiah D. Momper^2^

Affiliations:

1. Department of Pathology, University of California San Diego
2. Center for Medicinal Cannabis Research Reference Laboratory, Skaggs School of Pharmacy and Pharmaceutical Sciences, University of California San Diego
3. Shu Chien-Gene Lay Department of Bioengineering, Jacobs School of Engineering, University of California San Diego
4. Center for Medicinal Cannabis Research, Department of Psychiatry, University of California San Diego

Corresponding Author: Raymond T. Suhandynata^1,2^ PhD DABCC

Email: rtsuhandynata@health.ucsd.edu

Supplementary Figure 1. Extracted ion chromatogram of internal standard (IS) components.

XICs for each IS were overlaid. The x-axis indicates retention time (min) and the y-axis indicates signal intensity (counts per second). **A)** and **B)** share the same x-axis. Two inset panels are shown in **B)**, each with a different y-axis scale to help visualize varying degrees of ionization efficiency. Each XIC trace is labeled with a number corresponding to the XIC index listed in **Supplementary Table 9**.

Supplementary Figure 2. Matrix and solvent recovery by peak area for unstable pesticides.

%recovery calculated as (peak area)/(peak area at time 0)*100 are plotted as bar graphs for consecutive injections of the same sample. Filled black bars correspond to recovery in SNM, and unfilled bars correspond to recovery in PEM. SNM: Solvent-only no-matrix; PEM: Post-extracted matrix.

Supplementary Table 1. Standard Materials

| **Compound** | **Molecular formula** | **CAS #** | **Vendor PID/Catalog #** | **Monoisotopic mass (Da)** |
| --- | --- | --- | --- | --- |
| Acephate | C_4_H_10_NO_3_PS | 30560-19-1 | AccuStandard/CA-CP-04 | 183.0119 |
| Acequinocyl | C_24_H_32_O_4_ | 57960-19-7 | AccuStandard/CA-CP-04 | 384.2301 |
| Acetamiprid | C_10_H_11_ClN_4_ | 135410-20-7 | AccuStandard/CA-CP-03 | 222.0672 |
| Aldicarb | C_7_H_14_N_2_O_2_S | 116-06-3 | AccuStandard/CA-CP-01 | 190.0776 |
| Avermectin B1a | C_48_H_72_O_14_ | 65195-55-3 | AccuStandard/CA-CP-04 | 872.4922 |
| Avermectin B1b | C_47_H_70_O_14_ | 65195-56-4 | AccuStandard/CA-CP-04 | 858.4766 |
| Azoxystrobin | C_22_H_17_N_3_O_5_ | 131860-33-8 | AccuStandard/CA-CP-03 | 403.1168 |
| Bifenazate | C_17_H_20_N_2_O_3_ | 149877-41-8 | AccuStandard/CA-CP-03 | 300.1474 |
| Bifenthrin | C_23_H_22_ClF_3_O_2_ | 82657-04-3 | AccuStandard/CA-CP-04 | 422.1260 |
| Boscalid | C_18_H_12_Cl_2_N_2_O | 188425-85-6 | AccuStandard/CA-CP-03 | 342.0327 |
| Captan | C_9_H_8_Cl_3_NO_2_S | 133-06-2 | AccuStandard/CA-CP-03 | 298.9341 |
| Carbaryl | C_12_H_11_NO_2_ | 63-25-2 | AccuStandard/CA-CP-03 | 201.0790 |
| Carbofuran | C_12_H_15_NO_3_ | 1563-66-2 | AccuStandard/CA-CP-01 | 221.1052 |
| Chlorantraniliprole | C_18_H_14_BrCl_2_N_5_O_2_ | 500008-45-7 | AccuStandard/CA-CP-03 | 480.9708 |
| Chlorfenapyr | C_15_H_11_BrClF_3_N_2_O | 122453-73-0 | AccuStandard/CA-CP-01 | 405.9695 |
| Chlorpyrifos | C_9_H_11_Cl_3_NO_3_PS | 2921-88-2 | AccuStandard/CA-CP-01 | 348.9263 |
| Cinerin I | C_20_H_28_O_3_ | 25402-06-6 | AccuStandard/CA-CP-04 | 316.2038 |
| Cinerin II | C_21_H_28_O_5_ | 121-20-0 | AccuStandard/CA-CP-04 | 360.1937 |
| Clofentezine | C_14_H_8_Cl_2_N_4_ | 74115-24-5 | AccuStandard/CA-CP-03 | 302.0126 |
| Coumaphos | C_14_H_16_ClO_5_PS | 56-72-4 | AccuStandard/CA-CP-01 | 362.0145 |
| Cyfluthrin | C_22_H_18_Cl_2_FNO_3_ | 68359-37-5 | AccuStandard/CA-CP-04 | 433.0648 |
| Cypermethrin | C_22_H_19_Cl_2_NO_3_ | 52315-07-8 | AccuStandard/CA-CP-04 | 415.0742 |
| Daminozide | C_6_H_12_N_2_O_3_ | 1596-84-5 | AccuStandard/CA-CP-01 | 160.0848 |
| Diazinon | C_12_H_21_N_2_O_3_PS | 333-41-5 | AccuStandard/CA-CP-04 | 304.1011 |
| Dichlorvos | C_4_H_7_Cl_2_O_4_P | 62-73-7 | AccuStandard/CA-CP-01 | 219.9459 |
| Dimethoate | C_5_H_12_NO_3_PS_2_ | 60-51-5 | AccuStandard/CA-CP-01 | 228.9996 |
| (E)-Dimethomorph | C_21_H_22_ClNO_4_ | 113210-97-2 | AccuStandard/CA-CP-03 | 387.1237 |
| (Z)-Dimethomorph | C_21_H_22_ClNO_4_ | 113210-98-3 | AccuStandard/CA-CP-03 | 387.1237 |
| Etoxazole | C_21_H_23_F_2_NO_2_ | 153233-91-1 | AccuStandard/CA-CP-03 | 359.1697 |
| Ethoprophos | C_8_H_19_O_2_PS_2_ | 13194-48-4 | AccuStandard/CA-CP-01 | 242.0564 |
| Etofenprox | C_25_H_28_O_3_ | 80844-07-1 | AccuStandard/CA-CP-01 | 376.2038 |
| Fenhexamid | C_14_H_17_Cl_2_NO_2_ | 126833-17-8 | AccuStandard/CA-CP-03 | 301.0636 |
| Fenoxycarb | C_17_H_19_NO_4_ | 72490-01-8 | AccuStandard/CA-CP-01 | 301.1314 |
| Fenpyroximate | C_24_H_27_N_3_O_4_ | 134098-61-6 | AccuStandard/CA-CP-03 | 421.2002 |
| Fipronil | C_12_H_4_Cl_2_F_6_N_4_OS | 120068-37-3 | AccuStandard/CA-CP-01 | 435.9387 |
| Flonicamid | C_9_H_6_F_3_N_3_O | 158062-67-0 | AccuStandard/CA-CP-03 | 229.0463 |
| Fludioxonil | C_12_H_6_F_2_N_2_O_2_ | 131341-86-1 | AccuStandard/CA-CP-03 | 248.0397 |
| Hexythiazox | C_17_H_21_ClN_2_O_2_S | 78587-05-0 | AccuStandard/CA-CP-03 | 352.1012 |
| Imazalil | C_14_H_14_Cl_2_N_2_O | 35554-44-0 | AccuStandard/CA-CP-01 | 296.0483 |
| Imidacloprid | C_9_H_10_ClN_5_O_2_ | 138261-41-3 | AccuStandard/CA-CP-03 | 255.0523 |
| Jasmolin I | C_21_H_30_O_3_ | 4466-14-2 | AccuStandard/CA-CP-04 | 330.2195 |
| Jasmolin II | C_22_H_30_O_5_ | 1172-63-0 | AccuStandard/CA-CP-04 | 374.2093 |
| Kresoxim Methyl | C_18_H_19_NO_4_ | 143390-89-0 | AccuStandard/CA-CP-03 | 313.1314 |
| Malathion | C_10_H_19_O_6_PS_2_ | 121-75-5 | AccuStandard/CA-CP-04 | 330.0361 |
| Metalaxyl | C_15_H_21_NO_4_ | 57837-19-1 | AccuStandard/CA-CP-03 | 279.1471 |
| Methiocarb | C_11_H_15_NO_2_S | 2032-65-7 | AccuStandard/CA-CP-01 | 225.0824 |
| Methomyl | C_5_H_10_N_2_O_2_S | 16752-77-5 | AccuStandard/CA-CP-03 | 162.0463 |
| Methyl Parathion | C_8_H_10_NO_5_PS | 298-00-0 | AccuStandard/CA-CP-01 | 263.0017 |
| (E)-Mevinphos | C_7_H_13_O_6_P | 7786-34-7 | AccuStandard/CA-CP-01 | 224.0450 |
| (Z)-Mevinphos | C_7_H_13_O_6_P | 7786-34-7 | AccuStandard/CA-CP-01 | 224.0450 |
| Myclobutanil | C_15_H_17_ClN_4_ | 88671-89-0 | AccuStandard/CA-CP-03 | 288.1142 |
| Naled | C_4_H_7_Br_2_Cl_2_O_4_P | 300-76-5 | AccuStandard/CA-CP-04 | 377.7826 |
| Oxamyl | C_7_H_13_N_3_O_3_S | 23135-22-0 | AccuStandard/CA-CP-03 | 219.0678 |
| Paclobutrazol | C_15_H_20_ClN_3_O | 76738-62-0 | AccuStandard/CA-CP-01 | 293.1295 |
| (E)-Permethrin | C_21_H_20_Cl_2_O_3_ | 61949-77-7 | AccuStandard/CA-CP-04 | 390.0789 |
| (Z)-Permethrin | C_21_H_20_Cl_2_O_3_ | 61949-76-6 | AccuStandard/CA-CP-04 | 390.0789 |
| Phosmet | C_11_H_12_NO_4_PS_2_ | 732-11-6 | AccuStandard/CA-CP-04 | 316.9945 |
| Piperonyl Butoxide | C_19_H_30_O_5_ | 51-03-6 | AccuStandard/CA-CP-04 | 338.2093 |
| Prallethrin | C_19_H_24_O_3_ | 23031-36-9 | AccuStandard/CA-CP-04 | 300.1725 |
| Propiconazole | C_15_H_17_Cl_2_N_3_O_2_ | 60207-90-1 | AccuStandard/CA-CP-03 | 341.0698 |
| Propoxur | C_11_H_15_NO_3_ | 114-26-1 | AccuStandard/CA-CP-01 | 209.1052 |
| Pyrethrin I | C_21_H_28_O_3_ | 121-21-1 | AccuStandard/CA-CP-04 | 328.2038 |
| Pyrethrin II | C_22_H_28_O_5_ | 121-29-9 | AccuStandard/CA-CP-04 | 372.1937 |
| Pyridaben | C_19_H_25_ClN_2_OS | 96489-71-3 | AccuStandard/CA-CP-03 | 364.1376 |
| Spinetoram J | C_42_H_69_NO_10_ | 187166-40-1 | AccuStandard/CA-CP-03 | 747.4921 |
| Spinetoram L | C_43_H_69_NO_10_ | 187166-15-0 | AccuStandard/CA-CP-03 | 759.4921 |
| Spinosyn A | C_41_H_65_NO_10_ | 131929-60-7 | AccuStandard/CA-CP-03 | 731.4608 |
| Spinosyn D | C_42_H_67_NO_10_ | 131929-63-0 | AccuStandard/CA-CP-03 | 745.4765 |
| Spiromesifen | C_23_H_30_O_4_ | 283594-90-1 | AccuStandard/CA-CP-04 | 370.2144 |
| Spirotetramat | C_21_H_27_NO_5_ | 203313-25-1 | AccuStandard/CA-CP-03 | 373.1889 |
| Spiroxamine | C_18_H_35_NO_2_ | 118134-30-8 | AccuStandard/CA-CP-01 | 297.2668 |
| Tebuconazole | C_16_H_22_ClN_3_O | 107534-96-3 | AccuStandard/CA-CP-03 | 307.1451 |
| Thiacloprid | C_10_H_9_ClN_4_S | 111988-49-9 | AccuStandard/CA-CP-01 | 252.0237 |
| Thiamethoxam | C_8_H_10_ClN_5_O_3_S | 153719-23-4 | AccuStandard/CA-CP-03 | 291.0193 |
| Trifloxystrobin | C_20_H_19_F_3_N_2_O_4_ | 141517-21-7 | AccuStandard/CA-CP-03 | 408.1297 |
| Acephate-d_3_ | C_4_H_7_D_3_NO_3_PS | 2140327-70-2 | Cayman/39579 | 186.0307 |
| Acequinocyl-d_25_ | C_24_H_7_D_25_O_4_ | 2469735-25-7 | Cayman/39589 | 409.3870 |
| Acetamiprid-d_3_ | C_10_D_3_H_8_ClN_4_ | 1353869-35-8 | Cayman/39565 | 225.0861 |
| Aldicarb-d_3_ | C_7_H_11_D_3_N_2_O_2_S | 1795142-83-4 | Cayman/39580 | 193.0964 |
| (D)-cis, trans-Allethrin | C_19_H_26_O_3_ | 584-79-2 | AccuStandard/P-267N | 302.1882 |
| Azoxystrobin-d_4_ | C_22_H_13_D_4_N_3_O_5_ | 1346606-39-0 | SigmaAldrich/51949 | 407.1419 |
| Bifenazate-d_5_ | C_17_H_15_D_5_N_2_O_3_ |  | Cayman/39592 | 305.1788 |
| Bifenthrin-d_5_ | C_23_H_17_ClD_5_F_3_O_2_ | 998-851-4 | Cayman/39588 | 427.1574 |
| Boscalid-d_4_ | C_18_H_8_D_4_Cl_2_N_2_O | 2468796-76-9 | TRC/TRC-B675852 | 346.0578 |
| Captan-d_6_ | C_9_H_2_Cl_3_D_6_NO_2_S | 1330190-00-5 | Cayman/39577 | 304.9718 |
| Carbaryl-d_7_ | C_12_H_4_D_7_NO_2_ | 362049-56-7 | Cayman/39550 | 208.1229 |
| Carbofuran-d_3_ | C_12_H_12_D_3_NO_3_ | 1007459-98-4 | Cayman/39558 | 224.1240 |
| Chlorantraniliprole-d_3_ | C_18_H_11_D_3_BrCl_2_N_5_O_2_ | 1392493-28-5 | TRC/C325384 | 483.9896 |
| Chlorfenapyr-d_7_ | C_15_H_4_BrD_7_ClF_3_N_2_O |  | Cayman/39599 | 413.0135 |
| Chlorpyrifos-d_10_ | C_9_HCl_3_D_10_NO_3_PS | 285138-81-0 | Cayman/30953 | 358.9891 |
| Clofentezine-d_8_ | C_14_D_8_Cl_2_N_4_ | 2732862-38-1 | LGC/TRC-C585616 | 310.0628 |
| Coumaphos-d_10_ | C_14_H_6_ClD_10_O_5_PS | 287397-86-8 | Cayman/39563 | 372.0772 |
| Cyfluthrin-d_6_ | C_22_H_12_Cl_2_D_6_FNO_3_ | 2483831-11-2 | Cayman/39600 | 439.1024 |
| Cypermethrin-d_9_ | C_22_H_10_D_9_Cl_2_NO_3_ |  | Cayman/39266 | 424.1307 |
| Daminozide-d_4_ | C_6_H_8_D_4_N_2_O_3_ |  | Cayman/39264 | 164.1099 |
| Danitol | C_22_H_23_NO_3_ | 39515-41-8 | AccuStandard/P-263N | 349.1678 |
| Diazinon-d_10_ | C_12_H_11_D_10_N_2_O_3_PS | 100155-47-3 | Cayman/39556 | 314.1638 |
| Dichlorvos-d_6_ | C_4_HCl_2_D_6_O_4_P | 203645-53-8 | Cayman/34517 | 225.9836 |
| Dimethoate-d_6_ | C_5_H_6_D_6_NO_3_PS_2_ | 1219794-81-6 | Cayman/39260 | 235.0373 |
| (E)-Dimethomorph-d_6_ | C_21_D_6_H_16_ClNO_4_ |  | SigmaAldrich/89953 | 393.1614 |
| (Z)-Dimethomorph-d_6_ | C_21_D_6_H_16_ClNO_4_ |  | SigmaAldrich/89953 | 393.1614 |
| Emamectin | C_49_H_75_NO_13_ | 119791-41-2 | Cayman/36201 | 885.5238 |
| Ethoprophos-d_6_ | C_8_H_13_D_6_O_2_PS_2_ | 2733774-72-4 | Cayman/39601 | 248.0941 |
| Etofenprox-d_5_ | C_25_H_23_D_5_O_3_ |  | LGC/DRE-XA13363010AC | 381.2352 |
| Etoxazole-d_5_ | C_21_H_18_D_5_F_2_NO_2_ |  | MCE/HY-W059342S | 364.2011 |
| Fenhexamid-d_3_ | C_14_H_14_D_3_Cl_2_NO_2_ | 2140327-31-5 | LGC/DRE-C13476010 | 304.0825 |
| Fenoxycarb-d_3_ | C_17_H_16_D_3_NO_4_ | 2468639-22-5 | Cayman/39598 | 304.1502 |
| Fipronil-^13^C_2_^15^N_2_ | C_10_^13^C_2_H_4_Cl_2_F_6_N_2_^15^N_2_OS |  | Cayman/39587 | 439.9395 |
| Flonicamid-^15^N^18^O | C_9_H_6_F_3_N_2_^15^N^18^O | 2483830-60-8 | CIL/39578 | 232.0476 |
| Fludioxonil-^13^C_3_ | C_9_^13^C_3_H_6_F_2_N_2_O_2_ | 1185003-07-9 | LGC/TRC-F427002 | 251.0498 |
| Hexythiazox-d_11_ | C_17_H_10_ClD_11_N_2_O_2_S |  | Cayman/39602 | 363.1703 |
| Imazalil-d_5_ | C_14_D_5_H_9_Cl_2_N_2_O |  | Cayman/39261 | 301.0797 |
| Imidacloprid-d_4_ | C_9_H_6_D_4_ClN_5_O_2_ | 1015855-75-0 | Cayman/39549 | 259.0774 |
| Ivermectin | C_48_H_74_O_14_ | 70288-86-7 | Cayman/35518 | 874.5079 |
| Kresoxim Methyl-d_7_ | C_18_H_12_D_7_NO_4_ |  | Cayman/39603 | 320.1753 |
| Malathion-d_6_ | C_10_H_13_D_6_O_6_PS_2_ | 1189877-72-2 | Cayman/39262 | 336.0737 |
| Metalaxyl-d_3_ | C_15_H_18_D_3_NO_4_ |  | SigmaAldrich/08963 | 282.1659 |
| Methomyl-d_3_ | C_5_H_7_D_3_N_2_O_2_S | 1398109-07-3 | Cayman/39576 | 165.0651 |
| Methiocarb-d_3_ | C_11_H_12_D_3_NO_2_S | 1581694-94-1 | Cayman/27776 | 228.1012 |
| Methyl Parathion-d_6_ | C_8_H_4_D_6_NO_5_PS | 96740-32-8 | Cayman/37894 | 269.0394 |
| (E)-Mevinphos-d_6_ | C_7_D_6_H_7_O_6_P |  | LGC/DRE-C15220010 | 230.0826 |
| (Z)-Mevinphos-d_6_ | C_7_D_6_H_7_O_6_P |  | LGC/DRE-C15220010 | 230.0826 |
| Myclobutanil-d_9_ | C_15_H_8_D_9_ClN_4_ |  | Cayman/39263 | 297.1707 |
| Naled-d_6_ | C_4_HD_6_Br_2_Cl_2_O_4_P | 1216605-57-0 | LGC/TRC-N245302 | 383.8202 |
| Oxamyl-d_6_ | C_7_H_7_D_6_N_3_O_3_S | 2470229-60-6 | Cayman/39031 | 225.1054 |
| Paclobutrazol-d_4_ | C_15_D_4_H_16_ClN_3_O |  | MCE/HY-W749242 | 297.1546 |
| Phosmet-d_6_ | C_11_H_6_D_6_NO_4_PS_2_ | 2083623-41-8 | Cayman/39569 | 323.0322 |
| (E)-Permethrin-d_5_ | C_21_D_5_H_15_Cl_2_O_3_ | 1794760-19-2 | SigmaAldrich/74567 | 395.1103 |
| (Z)-Permethrin-d_5_ | C_21_D_5_H_15_Cl_2_O_3_ | 1794760-19-2 | SigmaAldrich/74567 | 395.1103 |
| Piperonyl Butoxide-d_9_ | C_19_H_21_D_9_O_5_ | 1329834-53-8 | Cayman/39581 | 347.2658 |
| Propoxur-d_3_ | C_11_H_12_D_3_NO_3_ | 1219798-56-7 | Cayman/39582 | 212.1240 |
| Propiconazole-d_3_ | C_15_H_14_D_3_Cl_2_N_3_O_2_ | 2699607-26-4 | Cayman/26788 | 344.0886 |
| Pyridaben-d_13_ | C_19_H_12_ClD_13_N_2_OS | 2468638-05-1 | Cayman/39259 | 377.2192 |
| Spiromesifen-d_9_ | C_23_H_21_D_9_O_4_ | 2470126-90-8 | Cayman/39033 | 379.2709 |
| Spirotetramat-d_5_ | C_21_H_22_D_5_NO_5_ | 2469007-09-6 | Cayman/39032 | 378.2203 |
| Spiroxamine-d_4_ | C_18_H_31_D_4_NO_2_ | 2469272-82-8 | Cayman/39604 | 301.2919 |
| Tebuconazole-d_9_ | C_16_H_13_ClD_9_N_3_O | 1246818-83-6 | Cayman/33287 | 316.2016 |
| Thiacloprid-d_4_ | C_10_H_5_ClD_4_N_4_S | 1793071-39-2 | SigmaAldrich/30673 | 256.0488 |
| Thiamethoxam-d_3_ | C_8_H_7_ClD_3_N_5_O_3_S | 1294048-82-0 | Cayman/35685 | 294.0381 |
| Trifloxystrobin-d_6_ | C_20_H_13_D_6_F_3_N_2_O_4_ | 2470226-50-5 | Cayman/39591 | 414.1674 |

Supplementary Table 2. Internal Standard Solutions

| **Compound** | **Primary IS (µg/mL)** | **Working IS (ng/mL)** | **In Vial (ng/mL)** |
| --- | --- | --- | --- |
| Acephate-d_3_ | 5 | 400 | 20 |
| Acequinocyl-d_25_ | 10 | 800 | 40 |
| Acetamiprid-d_3_ | 5 | 400 | 20 |
| Aldicarb-d_3_ | 5 | 400 | 20 |
| Allethrin | 5 | 400 | 20 |
| Azoxystrobin-d_4_ | 5 | 400 | 20 |
| Bifenazate-d_5_ | 2.5 | 200 | 10 |
| Bifenthrin-d_5_ | 5 | 400 | 20 |
| Boscalid-d_4_ | 5 | 400 | 20 |
| Captan-d_6_ | 50 | 4000 | 200 |
| Carbaryl-d_7_ | 5 | 400 | 20 |
| Carbofuran-d_3_ | 5 | 400 | 20 |
| Chlorfenapyr-d_7_ | 25 | 2000 | 100 |
| Chlorpyrifos-d_10_ | 5 | 400 | 20 |
| Chorantraniliprole-d_3_ | 5 | 400 | 20 |
| Clofentezine-d_8_ | 5 | 400 | 20 |
| Coumaphos-d_10_ | 5 | 400 | 20 |
| Cyfluthrin-d_6_ | 20 | 1600 | 80 |
| Cypermethrin-d_9_ | 25 | 2000 | 100 |
| Daminozide-d_4_ | 5 | 400 | 20 |
| Danitol | 5 | 400 | 20 |
| Diazinon-d_10_ | 5 | 400 | 20 |
| Dichlorvos-d_6_ | 5 | 400 | 20 |
| Dimethoate-d_6_ | 5 | 400 | 20 |
| Dimethomorph-d_6_ (mixture of isomers) | 5 | 400 | 20 |
| Emamectin | 5 | 400 | 20 |
| Ethoprophos-d_6_ | 5 | 400 | 20 |
| Etofenprox-d_5_ | 5 | 400 | 20 |
| Etoxazole-d_5_ | 5 | 400 | 20 |
| Fenhexamid-d_3_ | 5 | 400 | 20 |
| Fenoxycarb-d_3_ | 5 | 400 | 20 |
| Fipronil-^13^C_2_,^15^N_2_ | 5 | 400 | 20 |
| Flonicamid-^15^N,^18^O | 5 | 400 | 20 |
| Fludioxonil-^13^C | 5 | 400 | 20 |
| Hexythiazox-d_11_ | 5 | 400 | 20 |
| Imazalil-d_5_ | 2.5 | 200 | 10 |
| Imidacloprid-d_4_ | 5 | 400 | 20 |
| Ivermectin | 10 | 800 | 40 |
| Kresoxim Methyl-d_7_ | 5 | 400 | 20 |
| Malathion-d_6_ | 2.5 | 200 | 10 |
| Metalaxyl-d_3_ | 5 | 400 | 20 |
| Methiocarb-d_3_ | 5 | 400 | 20 |
| Methomyl-d_3_ | 40 | 3200 | 160 |
| Methyl Parathion-d_6_ | 25 | 2000 | 100 |
| Mevinphos-d_6_ (mixture of isomers) | 5 | 400 | 20 |
| Myclobutanil-d_9_ | 5 | 400 | 20 |
| Naled-d_6_ | 5 | 400 | 20 |
| Oxamyl-d_6_ | 5 | 400 | 20 |
| Paclobutrazol-d_4_ | 5 | 400 | 20 |
| Permethrin-d_5_ | 5 | 400 | 20 |
| Phosmet-d_6_ | 5 | 400 | 20 |
| Piperonyl Butoxide-d_9_ | 2.5 | 200 | 10 |
| Propiconazole-d_3_ | 5 | 400 | 20 |
| Propoxur-d_3_ | 5 | 400 | 20 |
| Pyridaben-d_13_ | 5 | 400 | 20 |
| Spiromesifen-d_9_ | 5 | 400 | 20 |
| Spirotetramat-d_5_ | 5 | 400 | 20 |
| Spiroxamine-d_4_ | 5 | 400 | 20 |
| Tebucanazole-d_9_ | 5 | 400 | 20 |
| Thiacloprid-d_4_ | 5 | 400 | 20 |
| Thiamethoxam-d_3_ | 5 | 400 | 20 |
| Trifloxystrobin-d_6_ | 5 | 400 | 20 |

Supplementary Table 3. Calibrator Concentrations (ng/mL in-vial)

| **Analyte** | **Calibrator Level** | | | | | | | | | | | | | | | | | |  |
| --- | --- | --- | --- | --- | --- | --- | --- | --- | --- | --- | --- | --- | --- | --- | --- | --- | --- | --- | --- |
|  | **1** | **2** | **3** | **4** | **5** | **6** | **7** | **8** | **9** | **10** | **11** | **12** | **13** | **14** | **15** | **16** | **17** | **18** | |
| Acephate |  |  | 0.024 | 0.049 | 0.098 | 0.195 | 0.391 | 0.781 | 1.563 | 3.125 | 6.25 | 12.5 | 18.75 | 25 | 37.5 | 50 | 75 | 100 | |
| Acequinocyl |  |  |  |  |  | 0.195 | 0.391 | 0.781 | 1.563 | 3.125 | 6.25 | 12.5 | 18.75 | 25 | 37.5 | 50 | 75 | 100 | |
| Acetamiprid |  |  | 0.024 | 0.049 | 0.098 | 0.195 | 0.391 | 0.781 | 1.563 | 3.125 | 6.25 | 12.5 | 18.75 | 25 | 37.5 | 50 | 75 | 100 | |
| Aldicarb |  |  |  | 0.049 | 0.098 | 0.195 | 0.391 | 0.781 | 1.563 | 3.125 | 6.25 | 12.5 | 18.75 | 25 | 37.5 | 50 | 75 | 100 | |
| Avermectin B1a |  |  |  |  |  |  |  |  | 1.261 | 2.522 | 5.044 | 10.09 | 15.13 | 20.18 | 30.26 | 40.35 | 60.53 |  | |
| Avermectin B1b |  |  |  |  |  |  |  |  |  |  |  | 2.413 | 3.619 | 4.825 | 7.238 | 9.65 | 14.48 | 19.3 | |
| Azoxystrobin |  | 0.012 | 0.024 | 0.049 | 0.098 | 0.195 | 0.391 | 0.781 | 1.563 | 3.125 | 6.25 | 12.5 | 18.75 | 25 | 37.5 | 50 | 75 | 100 | |
| Bifenazate |  |  |  |  |  |  | 0.391 | 0.781 | 1.563 | 3.125 | 6.25 | 12.5 | 18.75 | 25 | 37.5 | 50 | 75 | 100 | |
| Bifenthrin |  |  |  |  |  |  | 0.391 | 0.781 | 1.563 | 3.125 | 6.25 | 12.5 | 18.75 | 25 | 37.5 | 50 | 75 | 100 | |
| Boscalid |  |  |  | 0.049 | 0.098 | 0.195 | 0.391 | 0.781 | 1.563 | 3.125 | 6.25 | 12.5 | 18.75 | 25 | 37.5 | 50 | 75 | 100 | |
| Captan |  |  |  |  |  |  |  |  |  |  | 6.25 | 12.5 | 18.75 | 25 | 37.5 | 50 | 75 | 100 | |
| Carbaryl |  |  | 0.024 | 0.049 | 0.098 | 0.195 | 0.391 | 0.781 | 1.563 | 3.125 | 6.25 | 12.5 | 18.75 | 25 | 37.5 | 50 | 75 | 100 | |
| Carbofuran |  |  |  | 0.049 | 0.098 | 0.195 | 0.391 | 0.781 | 1.563 | 3.125 | 6.25 | 12.5 | 18.75 | 25 | 37.5 |  | 75 |  | |
| Chlorantraniliprole |  |  |  |  | 0.098 | 0.195 | 0.391 | 0.781 | 1.563 | 3.125 | 6.25 | 12.5 | 18.75 | 25 | 37.5 | 50 | 75 | 100 | |
| Chlorfenapyr |  |  |  |  |  |  |  |  |  | 3.125 | 6.25 | 12.5 | 18.75 | 25 | 37.5 | 50 | 75 | 100 | |
| Chlorpyrifos |  |  |  | 0.049 | 0.098 | 0.195 | 0.391 | 0.781 | 1.563 | 3.125 | 6.25 | 12.5 | 18.75 | 25 | 37.5 | 50 | 75 | 100 | |
| Cinerin I |  |  |  |  |  |  | 0.022 | 0.045 | 0.089 | 0.178 | 0.356 | 0.713 | 1.069 | 1.425 | 2.138 | 2.85 | 4.275 | 5.7 | |
| Cinerin II |  |  |  |  |  |  |  | 0.03 | 0.059 | 0.119 | 0.238 | 0.475 | 0.713 | 0.95 | 1.425 | 1.9 | 2.85 | 3.8 | |
| Clofentezine |  |  | 0.024 | 0.049 | 0.098 | 0.195 | 0.391 | 0.781 | 1.563 | 3.125 | 6.25 | 12.5 | 18.75 | 25 | 37.5 | 50 | 75 | 100 | |
| Coumaphos |  |  |  | 0.049 | 0.098 | 0.195 | 0.391 | 0.781 | 1.563 | 3.125 | 6.25 | 12.5 | 18.75 | 25 | 37.5 | 50 | 75 | 100 | |
| Cyfluthrin |  |  |  |  |  |  |  |  |  | 3.125 | 6.25 | 12.5 | 18.75 | 25 | 37.5 | 50 | 75 | 100 | |
| Cypermethrin |  |  |  |  |  |  |  |  | 1.563 | 3.125 | 6.25 | 12.5 | 18.75 | 25 | 37.5 | 50 | 75 | 100 | |
| Daminozide |  |  |  |  | 0.098 | 0.195 | 0.391 | 0.781 | 1.563 | 3.125 | 6.25 | 12.5 | 18.75 | 25 | 37.5 | 50 | 75 | 100 | |
| Diazinon |  | 0.012 | 0.024 | 0.049 | 0.098 | 0.195 | 0.391 | 0.781 | 1.563 | 3.125 | 6.25 | 12.5 | 18.75 | 25 | 37.5 | 50 | 75 | 100 | |
| Dichlorvos |  |  |  |  |  | 0.195 | 0.391 | 0.781 | 1.563 | 3.125 | 6.25 | 12.5 | 18.75 | 25 | 37.5 | 50 | 75 | 100 | |
| Dimethoate |  |  | 0.024 | 0.049 | 0.098 | 0.195 | 0.391 | 0.781 | 1.563 | 3.125 | 6.25 | 12.5 | 18.75 | 25 | 37.5 | 50 | 75 | 100 | |
| (E)-Dimethomorph |  |  |  |  |  | 0.084 | 0.168 | 0.336 | 0.672 | 1.344 | 2.688 | 5.375 | 8.063 | 10.75 | 16.13 | 21.5 | 32.25 | 43 | |
| (Z)-Dimethomorph |  |  |  |  | 0.056 | 0.111 | 0.223 | 0.445 | 0.891 | 1.781 | 3.563 | 7.125 | 10.69 | 14.25 | 21.38 | 28.5 | 42.75 | 57 | |
| Etoxazole |  |  |  |  | 0.098 | 0.195 | 0.391 | 0.781 | 1.563 | 3.125 | 6.25 | 12.5 | 18.75 | 25 | 37.5 | 50 |  |  | |
| Ethoprophos |  | 0.012 | 0.024 | 0.049 | 0.098 | 0.195 | 0.391 | 0.781 | 1.563 | 3.125 | 6.25 | 12.5 | 18.75 | 25 | 37.5 | 50 | 75 | 100 | |
| Etofenprox |  |  |  |  | 0.098 | 0.195 | 0.391 | 0.781 | 1.563 | 3.125 | 6.25 | 12.5 | 18.75 | 25 | 37.5 | 50 | 75 | 100 | |
| Fenhexamid |  |  |  |  |  |  | 0.391 | 0.781 | 1.563 | 3.125 | 6.25 | 12.5 | 18.75 | 25 | 37.5 | 50 |  |  | |
| Fenoxycarb |  |  |  | 0.049 | 0.098 | 0.195 | 0.391 | 0.781 | 1.563 | 3.125 | 6.25 | 12.5 | 18.75 | 25 | 37.5 | 50 | 75 | 100 | |
| Fenpyroximate |  |  |  |  | 0.098 | 0.195 | 0.391 | 0.781 | 1.563 | 3.125 | 6.25 | 12.5 | 18.75 | 25 | 37.5 | 50 | 75 |  | |
| Fipronil |  |  |  |  | 0.098 | 0.195 | 0.391 | 0.781 | 1.563 | 3.125 | 6.25 | 12.5 | 18.75 | 25 | 37.5 | 50 | 75 |  | |
| Flonicamid |  |  | 0.024 | 0.049 | 0.098 | 0.195 | 0.391 | 0.781 | 1.563 | 3.125 | 6.25 | 12.5 | 18.75 | 25 | 37.5 | 50 | 75 | 100 | |
| Fludioxonil |  |  |  |  |  | 0.195 | 0.391 | 0.781 | 1.563 | 3.125 | 6.25 | 12.5 | 18.75 | 25 | 37.5 | 50 | 75 | 100 | |
| Hexythiazox |  |  |  | 0.049 | 0.098 | 0.195 | 0.391 | 0.781 | 1.563 | 3.125 | 6.25 | 12.5 | 18.75 | 25 | 37.5 | 50 | 75 |  | |
| Imazalil | 0.006 | 0.012 | 0.024 | 0.049 | 0.098 | 0.195 | 0.391 | 0.781 | 1.563 | 3.125 | 6.25 | 12.5 | 18.75 | 25 | 37.5 | 50 | 75 | 100 | |
| Imidacloprid | 0.006 | 0.012 | 0.024 | 0.049 | 0.098 | 0.195 | 0.391 | 0.781 | 1.563 | 3.125 | 6.25 | 12.5 | 18.75 | 25 | 37.5 | 50 | 75 | 100 | |
| Jasmolin I |  |  |  |  |  |  |  |  | 0.063 | 0.125 | 0.25 | 0.5 | 0.75 | 1 | 1.5 | 2 | 3 |  | |
| Jasmolin II |  |  |  |  |  |  |  |  |  | 0.084 | 0.169 | 0.338 | 0.506 | 0.675 | 1.013 | 1.35 | 2.025 | 2.7 | |
| Kresoxim Methyl |  |  |  |  | 0.098 | 0.195 | 0.391 | 0.781 | 1.563 | 3.125 | 6.25 | 12.5 | 18.75 | 25 | 37.5 | 50 | 75 | 100 | |
| Malathion |  | 0.012 | 0.024 | 0.049 | 0.098 | 0.195 | 0.391 | 0.781 | 1.563 | 3.125 | 6.25 | 12.5 | 18.75 | 25 | 37.5 | 50 | 75 | 100 | |
| Metalaxyl |  |  |  | 0.049 | 0.098 | 0.195 | 0.391 | 0.781 | 1.563 | 3.125 | 6.25 | 12.5 | 18.75 | 25 | 37.5 | 50 | 75 | 100 | |
| Methiocarb |  |  |  |  | 0.098 | 0.195 | 0.391 | 0.781 | 1.563 | 3.125 | 6.25 | 12.5 | 18.75 | 25 | 37.5 | 50 | 75 |  | |
| Methomyl |  |  |  | 0.049 | 0.098 | 0.195 | 0.391 | 0.781 | 1.563 | 3.125 | 6.25 | 12.5 | 18.75 | 25 | 37.5 | 50 | 75 | 100 | |
| Methyl Parathion |  |  |  |  |  |  |  |  | 1.563 | 3.125 | 6.25 | 12.5 | 18.75 | 25 | 37.5 | 50 | 75 | 100 | |
| (Z)-Mevinphos |  |  |  |  | 0.011 | 0.021 | 0.043 | 0.086 | 0.172 | 0.344 | 0.688 | 1.375 | 2.063 | 2.75 | 4.125 | 5.5 | 8.25 | 11 | |
| (E)-Mevinphos | 0.005 | 0.011 | 0.022 | 0.043 | 0.087 | 0.174 | 0.348 | 0.695 | 1.391 | 2.781 | 5.563 | 11.125 | 16.69 | 22.25 | 33.38 | 44.5 | 66.75 | 89 | |
| Myclobutanil |  |  |  |  | 0.098 | 0.195 | 0.391 | 0.781 | 1.563 | 3.125 | 6.25 | 12.5 | 18.75 | 25 | 37.5 | 50 | 75 |  | |
| Naled |  |  |  |  |  |  | 0.391 | 0.781 | 1.563 | 3.125 | 6.25 | 12.5 | 18.75 | 25 | 37.5 | 50 | 75 | 100 | |
| Oxamyl |  |  | 0.024 | 0.049 | 0.098 | 0.195 | 0.391 | 0.781 | 1.563 | 3.125 | 6.25 | 12.5 | 18.75 | 25 | 37.5 | 50 | 75 | 100 | |
| Paclobutrazol |  |  |  |  |  | 0.195 | 0.391 | 0.781 | 1.563 | 3.125 | 6.25 | 12.5 | 18.75 | 25 | 37.5 | 50 | 75 | 100 | |
| (E)-Permethrin |  |  |  |  |  | 0.107 | 0.215 | 0.43 | 0.859 | 1.719 | 3.438 | 6.875 | 10.31 | 13.75 | 20.63 | 27.5 | 41.25 | 55 | |
| (Z)-Permethrin |  |  |  |  |  |  |  | 0.352 | 0.703 | 1.406 | 2.813 | 5.625 | 8.438 | 11.25 | 16.88 | 22.5 | 33.75 | 45 | |
| Phosmet |  |  |  |  |  |  | 0.391 | 0.781 | 1.563 | 3.125 | 6.25 | 12.5 | 18.75 | 25 | 37.5 | 50 | 75 | 100 | |
| Piperonyl Butoxide |  |  |  | 0.049 | 0.098 | 0.195 | 0.391 | 0.781 | 1.563 | 3.125 | 6.25 | 12.5 | 18.75 | 25 | 37.5 | 50 | 75 | 100 | |
| Prallethrin |  |  |  |  |  |  | 0.391 | 0.781 | 1.563 | 3.125 | 6.25 | 12.5 | 18.75 | 25 | 37.5 | 50 | 75 | 100 | |
| Propiconazole |  |  |  |  |  | 0.195 | 0.391 | 0.781 | 1.563 | 3.125 | 6.25 | 12.5 | 18.75 | 25 | 37.5 | 50 | 75 | 100 | |
| Propoxur |  |  |  |  | 0.098 | 0.195 | 0.391 | 0.781 | 1.563 | 3.125 | 6.25 | 12.5 | 18.75 | 25 | 37.5 | 50 | 75 | 100 | |
| Pyrethrin I |  |  |  |  |  |  | 0.219 | 0.438 | 0.877 | 1.753 | 3.506 | 7.013 | 10.52 | 14.03 | 21.04 | 28.05 | 42.08 | 56.1 | |
| Pyrethrin II |  |  |  |  |  |  |  | 0.217 | 0.434 | 0.869 | 1.738 | 3.475 | 5.213 | 6.95 | 10.43 | 13.9 | 20.85 | 27.8 | |
| Pyridaben |  |  |  |  |  |  |  | 0.781 | 1.563 | 3.125 | 6.25 | 12.5 | 18.75 | 25 | 37.5 | 50 |  |  | |
| Spinetoram J |  |  |  | 0.035 | 0.07 | 0.14 | 0.28 | 0.559 | 1.119 | 2.238 | 4.475 | 8.95 | 13.43 | 17.9 | 26.85 | 35.8 | 53.7 | 71.6 | |
| Spinetoram L |  |  |  |  |  | 0.055 | 0.111 | 0.222 | 0.444 | 0.888 | 1.775 | 3.55 | 5.325 | 7.1 | 10.65 | 14.2 | 21.3 | 28.4 | |
| Spinosyn A |  |  |  |  | 0.079 | 0.159 | 0.317 | 0.634 | 1.269 | 2.538 | 5.075 | 10.15 | 15.23 | 20.3 | 30.45 | 40.6 | 60.9 |  | |
| Spinosyn D |  |  |  |  |  |  |  | 0.132 | 0.264 | 0.528 | 1.056 | 2.113 | 3.169 | 4.225 | 6.338 | 8.45 | 12.68 | 16.9 | |
| Spiromesifen |  |  |  |  | 0.098 | 0.195 | 0.391 | 0.781 | 1.563 | 3.125 | 6.25 | 12.5 | 18.75 | 25 | 37.5 | 50 |  |  | |
| Spirotetramat | 0.006 | 0.012 | 0.024 | 0.049 | 0.098 | 0.195 | 0.391 | 0.781 | 1.563 | 3.125 | 6.25 | 12.5 | 18.75 | 25 | 37.5 | 50 | 75 |  | |
| Spiroxamine |  |  | 0.024 | 0.049 | 0.098 | 0.195 | 0.391 | 0.781 | 1.563 | 3.125 | 6.25 | 12.5 | 18.75 | 25 | 37.5 | 50 | 75 | 100 | |
| Tebuconazole |  |  |  |  |  | 0.195 | 0.391 | 0.781 | 1.563 | 3.125 | 6.25 | 12.5 | 18.75 | 25 | 37.5 | 50 | 75 | 100 | |
| Thiacloprid |  | 0.012 | 0.024 | 0.049 | 0.098 | 0.195 | 0.391 | 0.781 | 1.563 | 3.125 | 6.25 | 12.5 | 18.75 | 25 | 37.5 | 50 | 75 | 100 | |
| Thiamethoxam |  |  | 0.024 | 0.049 | 0.098 | 0.195 | 0.391 | 0.781 | 1.563 | 3.125 | 6.25 | 12.5 | 18.75 | 25 | 37.5 | 50 | 75 | 100 | |
| Trifloxystrobin |  |  |  |  |  | 0.195 | 0.391 | 0.781 | 1.563 | 3.125 | 6.25 | 12.5 | 18.75 | 25 | 37.5 | 50 | 75 | 100 | |

Supplementary Table 4. Calibrator Concentration (ppb in-sample)

| **Analyte** | **Calibrator Level** | | | | | | | | | | | | | | | | | |
| --- | --- | --- | --- | --- | --- | --- | --- | --- | --- | --- | --- | --- | --- | --- | --- | --- | --- | --- |
|  | **1** | **2** | **3** | **4** | **5** | **6** | **7** | **8** | **9** | **10** | **11** | **12** | **13** | **14** | **15** | **16** | **17** | **18** |
| Acephate |  |  | 0.98 | 1.952 | 3.908 | 7.812 | 15.64 | 31.24 | 62.52 | 125 | 250 | 500 | 750 | 1000 | 1500 | 2000 | 3000 | 4000 |
| Acequinocyl |  |  |  |  |  | 7.812 | 15.64 | 31.24 | 62.52 | 125 | 250 | 500 | 750 | 1000 | 1500 | 2000 | 3000 | 4000 |
| Acetamiprid |  |  | 0.976 | 1.952 | 3.908 | 7.812 | 15.64 | 31.24 | 62.52 | 125 | 250 | 500 | 750 | 1000 | 1500 | 2000 | 3000 | 4000 |
| Aldicarb |  |  |  | 1.952 | 3.908 | 7.812 | 15.64 | 31.24 | 62.52 | 125 | 250 | 500 | 750 | 1000 | 1500 | 2000 | 3000 | 4000 |
| Avermectin B1a |  |  |  |  |  |  |  |  | 50.44 | 100.9 | 201.8 | 403.5 | 605.2 | 807 | 1211 | 1614 | 2421 |  |
| Avermectin B1b |  |  |  |  |  |  |  |  |  |  |  | 96.52 | 144.8 | 193 | 289.5 | 386 | 579 | 772 |
| Azoxystrobin |  | 0.488 | 0.976 | 1.952 | 3.908 | 7.812 | 15.64 | 31.24 | 62.52 | 125 | 250 | 500 | 750 | 1000 | 1500 | 2000 | 3000 | 4000 |
| Bifenazate |  |  |  |  |  |  | 15.64 | 31.24 | 62.52 | 125 | 250 | 500 | 750 | 1000 | 1500 | 2000 | 3000 | 4000 |
| Bifenthrin |  |  |  |  |  |  | 15.64 | 31.24 | 62.52 | 125 | 250 | 500 | 750 | 1000 | 1500 | 2000 | 3000 | 4000 |
| Boscalid |  |  |  | 1.952 | 3.908 | 7.812 | 15.64 | 31.24 | 62.52 | 125 | 250 | 500 | 750 | 1000 | 1500 | 2000 | 3000 | 4000 |
| Captan |  |  |  |  |  |  |  |  |  |  | 250 | 500 | 750 | 1000 | 1500 | 2000 | 3000 | 4000 |
| Carbaryl |  |  | 0.976 | 1.952 | 3.908 | 7.812 | 15.64 | 31.24 | 62.52 | 125 | 250 | 500 | 750 | 1000 | 1500 | 2000 | 3000 | 4000 |
| Carbofuran |  |  |  | 1.952 | 3.908 | 7.812 | 15.64 | 31.24 | 62.52 | 125 | 250 | 500 | 750 | 1000 | 1500 |  | 3000 |  |
| Chlorantraniliprole |  |  |  |  | 3.908 | 7.812 | 15.64 | 31.24 | 62.52 | 125 | 250 | 500 | 750 | 1000 | 1500 | 2000 | 3000 | 4000 |
| Chlorfenapyr |  |  |  |  |  |  |  |  |  | 125 | 250 | 500 | 750 | 1000 | 1500 | 2000 | 3000 | 4000 |
| Chlorpyrifos |  |  |  | 1.952 | 3.908 | 7.812 | 15.64 | 31.24 | 62.52 | 125 | 250 | 500 | 750 | 1000 | 1500 | 2000 | 3000 | 4000 |
| Cinerin I |  |  |  |  |  |  | 0.88 | 1.8 | 3.56 | 7.12 | 14.24 | 28.52 | 42.76 | 57 | 85.52 | 114 | 171 | 228 |
| Cinerin II |  |  |  |  |  |  |  | 1.2 | 2.36 | 4.76 | 9.52 | 19 | 28.52 | 38 | 57 | 76 | 114 | 152 |
| Clofentezine |  |  | 0.976 | 1.952 | 3.908 | 7.812 | 15.64 | 31.24 | 62.52 | 125 | 250 | 500 | 750 | 1000 | 1500 | 2000 | 3000 | 4000 |
| Coumaphos |  |  |  | 1.952 | 3.908 | 7.812 | 15.64 | 31.24 | 62.52 | 125 | 250 | 500 | 750 | 1000 | 1500 | 2000 | 3000 | 4000 |
| Cyfluthrin |  |  |  |  |  |  |  |  |  | 125 | 250 | 500 | 750 | 1000 | 1500 | 2000 | 3000 | 4000 |
| Cypermethrin |  |  |  |  |  |  |  |  | 62.52 | 125 | 250 | 500 | 750 | 1000 | 1500 | 2000 | 3000 | 4000 |
| Daminozide |  |  |  |  | 3.908 | 7.812 | 15.64 | 31.24 | 62.52 | 125 | 250 | 500 | 750 | 1000 | 1500 | 2000 | 3000 | 4000 |
| Diazinon |  | 0.488 | 0.976 | 1.952 | 3.908 | 7.812 | 15.64 | 31.24 | 62.52 | 125 | 250 | 500 | 750 | 1000 | 1500 | 2000 | 3000 | 4000 |
| Dichlorvos |  |  |  |  |  | 7.812 | 15.64 | 31.24 | 62.52 | 125 | 250 | 500 | 750 | 1000 | 1500 | 2000 | 3000 | 4000 |
| Dimethoate |  |  | 0.976 | 1.952 | 3.908 | 7.812 | 15.64 | 31.24 | 62.52 | 125 | 250 | 500 | 750 | 1000 | 1500 | 2000 | 3000 | 4000 |
| (E)-Dimethomorph |  |  |  |  |  | 3.36 | 6.72 | 13.44 | 26.88 | 53.76 | 107.5 | 215 | 322.5 | 430 | 645 | 860 | 1290 | 1720 |
| (Z)-Dimethomorph |  |  |  |  | 2.228 | 4.452 | 8.92 | 17.8 | 35.64 | 71.24 | 142.5 | 285 | 427.5 | 570 | 855 | 1140 | 1710 | 2280 |
| Etoxazole |  |  |  |  | 3.908 | 7.812 | 15.64 | 31.24 | 62.52 | 125 | 250 | 500 | 750 | 1000 | 1500 | 2000 |  |  |
| Ethoprophos |  | 0.488 | 0.976 | 1.952 | 3.908 | 7.812 | 15.64 | 31.24 | 62.52 | 125 | 250 | 500 | 750 | 1000 | 1500 | 2000 | 3000 | 4000 |
| Etofenprox |  |  |  |  | 3.908 | 7.812 | 15.64 | 31.24 | 62.52 | 125 | 250 | 500 | 750 | 1000 | 1500 | 2000 | 3000 | 4000 |
| Fenhexamid |  |  |  |  |  |  | 15.64 | 31.24 | 62.52 | 125 | 250 | 500 | 750 | 1000 | 1500 | 2000 |  |  |
| Fenoxycarb |  |  |  | 1.952 | 3.908 | 7.812 | 15.64 | 31.24 | 62.52 | 125 | 250 | 500 | 750 | 1000 | 1500 | 2000 | 3000 | 4000 |
| Fenpyroximate |  |  |  |  | 3.908 | 7.812 | 15.64 | 31.24 | 62.52 | 125 | 250 | 500 | 750 | 1000 | 1500 | 2000 | 3000 |  |
| Fipronil |  |  |  |  | 3.908 | 7.812 | 15.64 | 31.24 | 62.52 | 125 | 250 | 500 | 750 | 1000 | 1500 | 2000 | 3000 |  |
| Flonicamid |  |  | 0.976 | 1.952 | 3.908 | 7.812 | 15.64 | 31.24 | 62.52 | 125 | 250 | 500 | 750 | 1000 | 1500 | 2000 | 3000 | 4000 |
| Fludioxonil |  |  |  |  |  | 7.812 | 15.64 | 31.24 | 62.52 | 125 | 250 | 500 | 750 | 1000 | 1500 | 2000 | 3000 | 4000 |
| Hexythiazox |  |  |  | 1.952 | 3.908 | 7.812 | 15.64 | 31.24 | 62.52 | 125 | 250 | 500 | 750 | 1000 | 1500 | 2000 | 3000 |  |
| Imazalil | 0.244 | 0.488 | 0.976 | 1.952 | 3.908 | 7.812 | 15.64 | 31.24 | 62.52 | 125 | 250 | 500 | 750 | 1000 | 1500 | 2000 | 3000 | 4000 |
| Imidacloprid | 0.244 | 0.488 | 0.976 | 1.952 | 3.908 | 7.812 | 15.64 | 31.24 | 62.52 | 125 | 250 | 500 | 750 | 1000 | 1500 | 2000 | 3000 | 4000 |
| Jasmolin I |  |  |  |  |  |  |  |  | 2.52 | 5 | 10 | 20 | 30 | 40 | 60 | 80 | 120 |  |
| Jasmolin II |  |  |  |  |  |  |  |  |  | 3.36 | 6.76 | 13.52 | 20.24 | 27 | 40.52 | 54 | 81 | 108 |
| Kresoxim Methyl |  |  |  |  | 3.908 | 7.812 | 15.64 | 31.24 | 62.52 | 125 | 250 | 500 | 750 | 1000 | 1500 | 2000 | 3000 | 4000 |
| Malathion |  | 0.488 | 0.976 | 1.952 | 3.908 | 7.812 | 15.64 | 31.24 | 62.52 | 125 | 250 | 500 | 750 | 1000 | 1500 | 2000 | 3000 | 4000 |
| Metalaxyl |  |  |  | 1.952 | 3.908 | 7.812 | 15.64 | 31.24 | 62.52 | 125 | 250 | 500 | 750 | 1000 | 1500 | 2000 | 3000 | 4000 |
| Methiocarb |  |  |  |  | 3.908 | 7.812 | 15.64 | 31.24 | 62.52 | 125 | 250 | 500 | 750 | 1000 | 1500 | 2000 | 3000 |  |
| Methomyl |  |  |  | 1.952 | 3.908 | 7.812 | 15.64 | 31.24 | 62.52 | 125 | 250 | 500 | 750 | 1000 | 1500 | 2000 | 3000 | 4000 |
| Methyl Parathion |  |  |  |  |  |  |  |  | 62.52 | 125 | 250 | 500 | 750 | 1000 | 1500 | 2000 | 3000 | 4000 |
| (Z)-Mevinphos |  |  |  |  | 0.428 | 0.86 | 1.72 | 3.44 | 6.88 | 13.76 | 27.52 | 55 | 82.52 | 110 | 165 | 220 | 330 | 440 |
| (E)-Mevinphos | 0.216 | 0.436 | 0.868 | 1.74 | 3.476 | 6.952 | 13.92 | 27.8 | 55.64 | 111.24 | 222.5 | 445 | 667.5 | 890 | 1335 | 1780 | 2670 | 3560 |
| Myclobutanil |  |  |  |  | 3.908 | 7.812 | 15.64 | 31.24 | 62.52 | 125 | 250 | 500 | 750 | 1000 | 1500 | 2000 | 3000 |  |
| Naled |  |  |  |  |  |  | 15.64 | 31.24 | 62.52 | 125 | 250 | 500 | 750 | 1000 | 1500 | 2000 | 3000 | 4000 |
| Oxamyl |  |  | 0.976 | 1.952 | 3.908 | 7.812 | 15.64 | 31.24 | 62.52 | 125 | 250 | 500 | 750 | 1000 | 1500 | 2000 | 3000 | 4000 |
| Paclobutrazol |  |  |  |  |  | 7.812 | 15.64 | 31.24 | 62.52 | 125 | 250 | 500 | 750 | 1000 | 1500 | 2000 | 3000 | 4000 |
| (E)-Permethrin |  |  |  |  |  | 4.296 | 8.6 | 17.2 | 34.36 | 68.76 | 137.5 | 275 | 412.5 | 550 | 825 | 1100 | 1650 | 2200 |
| (Z)-Permethrin |  |  |  |  |  |  |  | 14.08 | 28.12 | 56.24 | 112.52 | 225 | 337.5 | 450 | 675 | 900 | 1350 | 1800 |
| Phosmet |  |  |  |  |  |  | 15.64 | 31.24 | 62.52 | 125 | 250 | 500 | 750 | 1000 | 1500 | 2000 | 3000 | 4000 |
| Piperonyl Butoxide |  |  |  | 1.952 | 3.908 | 7.812 | 15.64 | 31.24 | 62.52 | 125 | 250 | 500 | 750 | 1000 | 1500 | 2000 | 3000 | 4000 |
| Prallethrin |  |  |  |  |  |  | 15.64 | 31.24 | 62.52 | 125 | 250 | 500 | 750 | 1000 | 1500 | 2000 | 3000 | 4000 |
| Propiconazole |  |  |  |  |  | 7.812 | 15.64 | 31.24 | 62.52 | 125 | 250 | 500 | 750 | 1000 | 1500 | 2000 | 3000 | 4000 |
| Propoxur |  |  |  |  | 3.908 | 7.812 | 15.64 | 31.24 | 62.52 | 125 | 250 | 500 | 750 | 1000 | 1500 | 2000 | 3000 | 4000 |
| Pyrethrin I |  |  |  |  |  |  | 8.76 | 17.52 | 35.08 | 70.12 | 140.2 | 280.5 | 420.8 | 561 | 841.5 | 1122 | 1683 | 2244 |
| Pyrethrin II |  |  |  |  |  |  |  | 8.68 | 17.36 | 34.76 | 69.52 | 139 | 208.5 | 278 | 417 | 556 | 834 | 1112 |
| Pyridaben |  |  |  |  |  |  |  | 31.24 | 62.52 | 125 | 250 | 500 | 750 | 1000 | 1500 | 2000 |  |  |
| Spinetoram J |  |  |  | 1.4 | 2.796 | 5.592 | 11.2 | 22.36 | 44.76 | 89.52 | 179 | 358 | 537 | 716 | 1074 | 1432 | 2148 | 2864 |
| Spinetoram L |  |  |  |  |  | 2.22 | 4.44 | 8.88 | 17.76 | 35.52 | 71 | 142 | 213 | 284 | 426 | 568 | 852 | 1136 |
| Spinosyn A |  |  |  |  | 3.172 | 6.344 | 12.68 | 25.36 | 50.76 | 101.5 | 203 | 406 | 609 | 812 | 1218 | 1624 | 2436 |  |
| Spinosyn D |  |  |  |  |  |  |  | 5.28 | 10.56 | 21.12 | 42.24 | 84.52 | 126.8 | 169 | 253.5 | 338 | 507 | 676 |
| Spiromesifen |  |  |  |  | 3.908 | 7.812 | 15.64 | 31.24 | 62.52 | 125 | 250 | 500 | 750 | 1000 | 1500 | 2000 |  |  |
| Spirotetramat | 0.244 | 0.488 | 0.976 | 1.952 | 3.908 | 7.812 | 15.64 | 31.24 | 62.52 | 125 | 250 | 500 | 750 | 1000 | 1500 | 2000 | 3000 |  |
| Spiroxamine |  |  | 0.976 | 1.952 | 3.908 | 7.812 | 15.64 | 31.24 | 62.52 | 125 | 250 | 500 | 750 | 1000 | 1500 | 2000 | 3000 | 4000 |
| Tebuconazole |  |  |  |  |  | 7.812 | 15.64 | 31.24 | 62.52 | 125 | 250 | 500 | 750 | 1000 | 1500 | 2000 | 3000 | 4000 |
| Thiacloprid |  | 0.488 | 0.976 | 1.952 | 3.908 | 7.812 | 15.64 | 31.24 | 62.52 | 125 | 250 | 500 | 750 | 1000 | 1500 | 2000 | 3000 | 4000 |
| Thiamethoxam |  |  | 0.976 | 1.952 | 3.908 | 7.812 | 15.64 | 31.24 | 62.52 | 125 | 250 | 500 | 750 | 1000 | 1500 | 2000 | 3000 | 4000 |
| Trifloxystrobin |  |  |  |  |  | 7.812 | 15.64 | 31.24 | 62.52 | 125 | 250 | 500 | 750 | 1000 | 1500 | 2000 | 3000 | 4000 |

Supplementary Table 5. Liquid Chromatography Method

| **Time (min)** | **MPA (%)** | **MPB (%)** | **Flow (mL/min)** |
| --- | --- | --- | --- |
| 0.00 | 80.00 | 20.00 | 0.35 |
| 0.50 | 80.00 | 20.00 | 0.35 |
| 7.00 | 45.00 | 55.00 | 0.35 |
| 9.00 | 38.00 | 62.00 | 0.35 |
| 13.00 | 30.00 | 70.00 | 0.35 |
| 16.00 | 22.50 | 77.50 | 0.35 |
| 19.00 | 15.00 | 85.00 | 0.35 |
| 25.00 | 0.00 | 100.00 | 0.35 |
| 30.00 | 0.00 | 100.00 | 0.35 |
| 30.01 | 80.00 | 20.00 | 0.35 |
| 40.01 | 80.00 | 20.00 | 0.35 |

**A:** 5 mM NH_4_HCOO/0.1% FA/dH_2_O

**B:** 5mM NH_4_HCOO/0.1% FA/MeOH

Supplementary Table 6. Multiple Reaction Monitoring (MRM) Parameters

| **Compound** | **RT (min)** | **MRM Window (RT +/- s)** | **Q1**  **(m/z)** | **Q3**  **(m/z)** | **Dwell time (ms)** | **DP (V)** | **EP (V)** | **CE (V)** | **CXP (V)** |
| --- | --- | --- | --- | --- | --- | --- | --- | --- | --- |
| Quantifier ions | | | | | | | | | |
| Acephate | 1.41 | 30 | 184.1 | 49 | 88.51 | 10.0 | 9.0 | 26.0 | 15.0 |
| Acequinocyl | 24.02 | 30 | 402.2 | 343.2 | 190.28 | 50.0 | 6.5 | 16.0 | 20.0 |
| Acetamiprid | 4.47 | 30 | 223.2 | 99 | 57.60 | 40.0 | 14.0 | 50.0 | 12.5 |
| Aldicarb | 5.84 | 30 | 208.1 | 88.9 | 109.23 | 25.0 | 13.0 | 22.0 | 10.0 |
| Avermectin B1a | 20.94 | 30 | 895.46 | 751 | 59.06 | 50.0 | 11.5 | 56.0 | 37.5 |
| Avermectin B1b | 20.26 | 30 | 881.418 | 737.399 | 52.74 | 50.0 | 11.5 | 55.0 | 37.5 |
| Azoxystrobin | 10.46 | 30 | 404.1 | 344 | 12.00 | 45.0 | 15.0 | 33.0 | 37.5 |
| Bifenazate | 11.84 | 30 | 301.1 | 198.1 | 40.89 | 20.0 | 12.0 | 12.0 | 10.0 |
| Bifenthrin | 22.09 | 30 | 440.2 | 166.1 | 87.95 | 20.0 | 12.0 | 57.0 | 12.0 |
| Boscalid | 10.91 | 45 | 343 | 307 | 19.29 | 60.0 | 12.0 | 27.0 | 25.0 |
| Captan | 9.61 | 30 | 317 | 264 | 34.42 | 30.0 | 8.0 | 16.0 | 15.0 |
| Carbaryl | 7.83 | 30 | 202.1 | 145 | 73.67 | 20.0 | 13.0 | 13.0 | 15.0 |
| Carbofuran | 7.36 | 30 | 221.896 | 164.9 | 55.41 | 30.0 | 13.0 | 16.0 | 15.0 |
| Chlorantraniliprole | 9.88 | 30 | 483.9 | 452.9 | 23.91 | 35.0 | 13.0 | 22.0 | 40.0 |
| Chlorfenapyr | 16.39 | 45 | 408.8 | 378.7 | 63.07 | 60.0 | 13.0 | 21.0 | 25.0 |
| Chlorpyrifos | 17.98 | 30 | 350 | 96.9 | 68.95 | 30.0 | 12.0 | 43.0 | 10.0 |
| Cinerin I | 18.94 | 30 | 317.3 | 149 | 46.08 | 25.0 | 12.0 | 12.0 | 15.0 |
| Cinerin II | 15.67 | 30 | 361.5 | 149 | 51.04 | 25.0 | 12.0 | 13.0 | 15.0 |
| Clofentezine | 14.82 | 30 | 303 | 102 | 77.86 | 20.0 | 12.0 | 50.0 | 15.0 |
| Coumaphos | 14.04 | 30 | 363 | 335 | 41.37 | 60.0 | 12.0 | 22.0 | 25.0 |
| Cyfluthrin | 19.59 | 45 | 453 | 192.8 | 40.83 | 20.0 | 9.5 | 19.0 | 10.0 |
| Cypermethrin | 19.86 | 45 | 435 | 192.8 | 44.28 | 25.0 | 12.0 | 20.0 | 10.0 |
| Daminozide | 1.05 | 30 | 161.2 | 143.1 | 117.76 | 25.0 | 9.0 | 15.0 | 10.0 |
| Diazinon | 14.12 | 30 | 305.1 | 153 | 44.61 | 30.0 | 12.0 | 27.0 | 12.5 |
| Dichlorvos | 7.01 | 30 | 222.927 | 108.9 | 85.68 | 50.0 | 13.0 | 22.0 | 10.0 |
| Dimethoate | 4.3 | 30 | 230 | 199 | 58.87 | 20.0 | 12.0 | 11.0 | 12.5 |
| (E)-Dimethomorph | 10.71 | 60 | 388 | 301 | 20.41 | 75.0 | 12.0 | 27.0 | 15.0 |
| (Z)-Dimethomorph | 11.31 | 30 | 388 | 301.01 | 23.80 | 75.0 | 12.0 | 27.0 | 17.0 |
| Etoxazole | 18.71 | 30 | 360.2 | 177.2 | 51.52 | 80.0 | 12.0 | 25.0 | 15.0 |
| Ethoprophos | 12.19 | 30 | 243 | 131 | 60.11 | 30.0 | 12.0 | 25.0 | 12.5 |
| Etofenprox | 21.69 | 30 | 394 | 177.3 | 63.34 | 25.0 | 12.0 | 19.0 | 15.0 |
| Fenhexamid | 12 | 30 | 302 | 97 | 50.76 | 75.0 | 13.0 | 28.0 | 15.0 |
| Fenoxycarb | 13.13 | 30 | 302.2 | 116 | 51.38 | 30.0 | 12.0 | 13.0 | 12.5 |
| Fenpyroximate | 19.24 | 30 | 422.2 | 135.1 | 20.00 | 60.0 | 12.0 | 40.0 | 15.0 |
| Fipronil | 13.03 | 30 | 437 | 367.6 | 55.91 | 100.0 | 12.0 | 22.0 | 25.0 |
| Flonicamid | 2.62 | 25 | 230 | 147.9 | 40.00 | 10.0 | 13.0 | 36.0 | 15.0 |
| Fludioxonil | 10.63 | 30 | 266 | 229 | 18.61 | 15.0 | 13.0 | 14.0 | 15.0 |
| Hexythiazox | 18.05 | 30 | 353.1 | 228 | 20.00 | 20.0 | 12.0 | 20.0 | 15.0 |
| Imazalil | 8.36 | 45 | 297.2 | 41.1 | 105.79 | 60.0 | 14.0 | 59.0 | 12.5 |
| Imidacloprid | 3.73 | 30 | 256.1 | 209 | 128.46 | 25.0 | 13.0 | 19.0 | 12.5 |
| Jasmolin I | 20.08 | 30 | 331 | 106.8 | 45.34 | 30.0 | 6.0 | 31.0 | 10.0 |
| Jasmolin II | 17.13 | 30 | 375 | 163 | 80.26 | 30.0 | 6.0 | 14.0 | 12.0 |
| Kresoxim Methyl | 13.48 | 30 | 314.1 | 115.9 | 39.79 | 20.0 | 12.0 | 17.0 | 15.0 |
| Malathion | 11.14 | 30 | 331.1 | 127.1 | 19.53 | 25.0 | 12.0 | 14.0 | 15.0 |
| Metalaxyl | 9.16 | 30 | 280.2 | 192.2 | 10.00 | 30.0 | 12.0 | 22.0 | 15.0 |
| Methiocarb | 10.44 | 30 | 226.1 | 169.2 | 19.82 | 20.0 | 12.0 | 12.0 | 10.0 |
| Methomyl | 2.54 | 25 | 163.1 | 88.01 | 40.00 | 10.0 | 13.0 | 12.0 | 15.0 |
| Methyl Parathion | 10.17 | 30 | 264 | 125 | 23.00 | 70.0 | 9.0 | 24.0 | 15.0 |
| (Z)-Mevinphos | 4.43 | 30 | 225 | 126.9 | 55.90 | 25.0 | 13.0 | 20.0 | 15.0 |
| (E)-Mevinphos | 5.46 | 30 | 225.06 | 126.96 | 88.92 | 25.0 | 13.0 | 19.0 | 15.0 |
| Myclobutanil | 11.41 | 30 | 289.1 | 70.1 | 26.50 | 55.0 | 12.5 | 20.0 | 12.5 |
| Naled | 9.37 | 30 | 380.8 | 127.1 | 45.15 | 35.0 | 13.0 | 16.0 | 12.5 |
| Oxamyl | 2.13 | 25 | 237.1 | 72.1 | 95.00 | 10.0 | 13.0 | 28.0 | 15.0 |
| Paclobutrazol | 11.01 | 30 | 294.1 | 70.1 | 18.54 | 45.0 | 13.0 | 21.0 | 10.0 |
| Permethrin I | 20.95 | 30 | 408.1 | 183.1 | 59.20 | 25.0 | 12.0 | 23.0 | 20.0 |
| Permethrin II | 21.51 | 30 | 408.1 | 183.11 | 59.82 | 25.0 | 12.0 | 29.0 | 20.0 |
| Phosmet | 9.85 | 30 | 318 | 160 | 24.49 | 25.0 | 12.0 | 16.0 | 15.0 |
| Piperonyl Butoxide | 17.47 | 30 | 356.2 | 119.2 | 67.08 | 20.0 | 12.0 | 47.0 | 12.0 |
| Prallethrin | 15.63 | 40 | 301.3 | 105.1 | 54.52 | 25.0 | 12.0 | 27.0 | 12.5 |
| Propiconazole | 14.05 | 30 | 342.1 | 159 | 41.59 | 50.0 | 12.0 | 35.0 | 12.5 |
| Propoxur | 7.21 | 30 | 210.1 | 111.1 | 59.24 | 20.0 | 13.5 | 18.0 | 12.5 |
| Pyrethrin I | 19.13 | 30 | 329.01 | 161.01 | 20.00 | 30.0 | 12.0 | 13.0 | 15.0 |
| Pyrethrin II | 16 | 30 | 373 | 161 | 20.00 | 30.0 | 12.0 | 14.0 | 15.0 |
| Pyridaben | 20.04 | 30 | 365.1 | 147 | 10.00 | 30.0 | 12.0 | 32.0 | 15.0 |
| Spinetoram J | 16.01 | 30 | 748.5 | 142.2 | 10.00 | 80.0 | 12.0 | 36.0 | 12.5 |
| Spinetoram L | 17.07 | 30 | 760.5 | 142.2 | 10.00 | 80.0 | 8.0 | 35.0 | 12.0 |
| Spinosyn A | 14.74 | 30 | 732.4 | 142.2 | 76.00 | 80.0 | 12.0 | 34.0 | 12.5 |
| Spinosyn D | 15.86 | 45 | 746.4 | 142.2 | 56.29 | 80.0 | 12.0 | 34.0 | 12.5 |
| Spiromesifen | 18.65 | 30 | 388.254 | 273.184 | 10.00 | 30.0 | 12.0 | 15.0 | 20.0 |
| Spirotetramat | 12.14 | 30 | 374.2 | 330 | 20.00 | 40.0 | 13.0 | 19.0 | 22.5 |
| Spiroxamine | 10.28 | 40 | 298.3 | 100.2 | 19.39 | 50.0 | 13.0 | 42.0 | 12.5 |
| Tebuconazole | 13.64 | 30 | 308.1 | 70.1 | 39.93 | 50.0 | 12.0 | 57.0 | 10.0 |
| Thiacloprid | 5.23 | 30 | 253.2 | 186 | 90.00 | 40.0 | 13.5 | 18.0 | 15.0 |
| Thiamethoxam | 2.72 | 25 | 292 | 181 | 90.00 | 10.0 | 13.0 | 28.0 | 15.0 |
| Trifloxystrobin | 16.03 | 30 | 409.1 | 116.1 | 47.23 | 30.0 | 12.0 | 27.0 | 10.0 |
| Qualifier ions | | | | | | | | | |
| Acephate | 1.41 | 30 | 201.015 | 124.9 | 88.51 | 10.0 | 9.0 | 29.0 | 15.0 |
| Acephate | 1.41 | 30 | 184.1 | 143 | 88.51 | 10.0 | 9.0 | 11.0 | 15.0 |
| Acequinocyl | 24.02 | 30 | 402.2 | 189.1 | 190.28 | 50.0 | 6.5 | 38.0 | 20.0 |
| Acetamiprid | 4.47 | 30 | 223.2 | 90 | 57.60 | 40.0 | 14.0 | 44.0 | 12.5 |
| Aldicarb | 5.84 | 30 | 208.1 | 116 | 109.23 | 25.0 | 13.0 | 10.0 | 10.0 |
| Avermectin B1a | 20.94 | 30 | 890.2 | 567.4 | 59.06 | 50.0 | 11.5 | 19.0 | 37.5 |
| Avermectin B1a | 20.94 | 30 | 890.2 | 305.3 | 59.06 | 50.0 | 11.5 | 33.0 | 37.5 |
| Avermectin B1b | 20.26 | 30 | 876.2 | 290.9 | 52.74 | 50.0 | 11.5 | 35.0 | 37.5 |
| Azoxystrobin | 10.46 | 30 | 404.1 | 372.1 | 10.00 | 45.0 | 15.0 | 19.0 | 37.5 |
| Bifenazate | 11.84 | 30 | 301.1 | 170.2 | 40.89 | 20.0 | 12.0 | 26.0 | 10.0 |
| Bifenthrin | 22.09 | 30 | 440.2 | 181.1 | 87.95 | 20.0 | 12.0 | 16.0 | 12.0 |
| Boscalid | 10.91 | 45 | 343 | 140 | 19.29 | 60.0 | 12.0 | 25.0 | 15.0 |
| Captan | 9.61 | 30 | 317 | 235.9 | 34.42 | 30.0 | 8.0 | 22.0 | 15.0 |
| Carbaryl | 7.83 | 30 | 202.1 | 127 | 73.67 | 20.0 | 13.0 | 35.0 | 15.0 |
| Carbofuran | 7.36 | 30 | 221.896 | 122.9 | 55.41 | 30.0 | 13.0 | 27.0 | 15.0 |
| Chlorantraniliprole | 9.88 | 30 | 483.9 | 285.9 | 23.91 | 35.0 | 13.0 | 17.0 | 40.0 |
| Chlorfenapyr | 16.39 | 45 | 407 | 376.9 | 63.07 | 60.0 | 13.0 | 21.0 | 25.0 |
| Chlorpyrifos | 17.98 | 30 | 350 | 198 | 68.95 | 30.0 | 12.0 | 24.0 | 10.0 |
| Cinerin I | 18.94 | 30 | 317.3 | 107 | 46.08 | 25.0 | 12.0 | 24.0 | 15.0 |
| Cinerin II | 15.67 | 30 | 361.5 | 107 | 51.04 | 25.0 | 12.0 | 27.0 | 15.0 |
| Clofentezine | 14.82 | 30 | 303 | 138 | 77.86 | 20.0 | 12.0 | 19.0 | 15.0 |
| Coumaphos | 14.04 | 30 | 363 | 226 | 41.37 | 60.0 | 12.0 | 51.0 | 25.0 |
| Cyfluthrin | 19.59 | 45 | 453 | 205.9 | 40.83 | 20.0 | 9.5 | 61.0 | 10.0 |
| Cypermethrin | 19.86 | 45 | 435 | 181.1 | 44.28 | 25.0 | 12.0 | 50.0 | 10.0 |
| Daminozide | 1.05 | 30 | 161.2 | 44 | 117.76 | 25.0 | 9.0 | 46.0 | 10.0 |
| Diazinon | 14.12 | 30 | 305.1 | 96.9 | 44.61 | 30.0 | 12.0 | 45.0 | 12.5 |
| Dichlorvos | 7.01 | 30 | 220.92 | 108.9 | 85.68 | 50.0 | 13.0 | 22.0 | 10.0 |
| Dimethoate | 4.3 | 30 | 230 | 125 | 58.87 | 20.0 | 12.0 | 26.0 | 12.5 |
| (E)-Dimethomorph | 10.71 | 60 | 388 | 165 | 20.41 | 75.0 | 12.0 | 40.0 | 15.0 |
| (Z)-Dimethomorph | 11.31 | 30 | 388 | 165.01 | 23.80 | 75.0 | 12.0 | 40.0 | 17.0 |
| Etoxazole | 18.71 | 30 | 360.2 | 141 | 51.52 | 80.0 | 12.0 | 34.0 | 15.0 |
| Ethoprophos | 12.19 | 30 | 243 | 97 | 60.11 | 30.0 | 12.0 | 40.0 | 12.5 |
| Etofenprox | 21.69 | 30 | 394 | 135.1 | 63.34 | 25.0 | 12.0 | 36.0 | 15.0 |
| Fenhexamid | 12 | 30 | 302 | 141.8 | 50.76 | 75.0 | 13.0 | 29.0 | 15.0 |
| Fenoxycarb | 13.13 | 30 | 302.2 | 87.9 | 51.38 | 30.0 | 12.0 | 26.0 | 12.5 |
| Fenpyroximate | 19.24 | 30 | 422.2 | 366.1 | 20.00 | 60.0 | 12.0 | 23.0 | 35.0 |
| Fipronil | 13.03 | 30 | 437 | 290 | 55.91 | 100.0 | 12.0 | 34.0 | 25.0 |
| Flonicamid | 2.62 | 25 | 230 | 176 | 40.00 | 10.0 | 13.0 | 31.0 | 15.0 |
| Flonicamid | 2.62 | 25 | 230 | 183.1 | 40.00 | 10.0 | 13.0 | 36.0 | 15.0 |
| Flonicamid | 2.62 | 25 | 230 | 155.1 | 40.00 | 10.0 | 13.0 | 36.0 | 15.0 |
| Fludioxonil | 10.63 | 30 | 266 | 158 | 18.61 | 15.0 | 13.0 | 44.0 | 15.0 |
| Hexythiazox | 18.05 | 30 | 353.1 | 168.1 | 20.00 | 20.0 | 12.0 | 34.0 | 15.0 |
| Imazalil | 8.36 | 45 | 297.2 | 159.1 | 105.79 | 60.0 | 14.0 | 29.0 | 12.5 |
| Imidacloprid | 3.73 | 30 | 256.1 | 175 | 128.46 | 25.0 | 13.0 | 24.0 | 12.5 |
| Jasmolin I | 20.08 | 30 | 331 | 79 | 45.34 | 30.0 | 6.0 | 50.0 | 10.0 |
| Jasmolin II | 17.13 | 30 | 375 | 107 | 80.26 | 30.0 | 6.0 | 35.0 | 12.0 |
| Kresoxim Methyl | 13.48 | 30 | 314.1 | 131 | 39.79 | 20.0 | 12.0 | 30.0 | 15.0 |
| Malathion | 11.14 | 30 | 331.1 | 285 | 19.53 | 25.0 | 12.0 | 8.0 | 15.0 |
| Metalaxyl | 9.16 | 30 | 280.2 | 148.1 | 10.00 | 30.0 | 12.0 | 27.0 | 15.0 |
| Metalaxyl | 9.16 | 30 | 280.2 | 160.2 | 10.00 | 30.0 | 12.0 | 28.0 | 15.0 |
| Metalaxyl | 9.16 | 30 | 280.2 | 248.1 | 10.00 | 30.0 | 12.0 | 13.0 | 15.0 |
| Methiocarb | 10.44 | 30 | 226.1 | 107 | 19.82 | 20.0 | 12.0 | 48.0 | 10.0 |
| Methomyl | 2.54 | 25 | 163.1 | 106 | 40.00 | 10.0 | 13.0 | 13.0 | 15.0 |
| Methomyl | 2.54 | 25 | 163.1 | 58 | 40.00 | 10.0 | 13.0 | 25.0 | 15.0 |
| Methyl Parathion | 10.17 | 30 | 264 | 232 | 23.00 | 70.0 | 9.0 | 24.0 | 15.0 |
| Methyl Parathion | 10.17 | 30 | 264 | 108.9 | 23.00 | 70.0 | 9.0 | 24.0 | 15.0 |
| (Z)-Mevinphos | 4.43 | 30 | 225 | 193 | 55.90 | 25.0 | 13.0 | 10.0 | 15.0 |
| (E)-Mevinphos | 5.46 | 30 | 225.06 | 193.06 | 88.92 | 25.0 | 13.0 | 9.0 | 15.0 |
| Myclobutanil | 11.41 | 30 | 289.1 | 125.2 | 26.50 | 55.0 | 12.5 | 45.0 | 12.5 |
| Naled | 9.37 | 30 | 382.8 | 127.1 | 45.15 | 35.0 | 13.0 | 16.0 | 12.5 |
| Oxamyl | 2.13 | 25 | 237.1 | 90.1 | 95.00 | 10.0 | 13.0 | 10.0 | 15.0 |
| Paclobutrazol | 11.01 | 30 | 294.1 | 125 | 18.54 | 45.0 | 13.0 | 51.0 | 10.0 |
| (E)-Permethrin | 20.95 | 30 | 408.1 | 355.1 | 59.20 | 25.0 | 12.0 | 10.0 | 20.0 |
| (Z)-Permethrin | 21.51 | 30 | 408.1 | 355.11 | 59.82 | 25.0 | 12.0 | 10.0 | 20.0 |
| Phosmet | 9.85 | 30 | 318 | 133.1 | 24.49 | 25.0 | 12.0 | 48.0 | 15.0 |
| Piperonyl Butoxide | 17.47 | 30 | 356.2 | 177.1 | 67.08 | 20.0 | 12.0 | 16.0 | 12.0 |
| Prallethrin | 15.63 | 40 | 301.3 | 133.1 | 54.52 | 25.0 | 12.0 | 15.0 | 12.5 |
| Propiconazole | 14.05 | 30 | 342.1 | 69.1 | 41.59 | 50.0 | 12.0 | 22.0 | 12.5 |
| Propoxur | 7.21 | 30 | 210.1 | 168.1 | 59.24 | 20.0 | 13.5 | 9.0 | 12.5 |
| Pyrethrin I | 19.13 | 30 | 329.01 | 143.01 | 20.00 | 30.0 | 12.0 | 23.0 | 15.0 |
| Pyrethrin II | 16 | 30 | 373 | 143 | 20.00 | 30.0 | 12.0 | 23.0 | 15.0 |
| Pyridaben | 20.04 | 30 | 365.1 | 309 | 10.00 | 30.0 | 12.0 | 17.0 | 15.0 |
| Spinetoram J | 16.01 | 30 | 748.5 | 98.1 | 10.00 | 80.0 | 12.0 | 101.0 | 12.5 |
| Spinetoram L | 17.07 | 30 | 760.5 | 98.1 | 10.00 | 80.0 | 8.0 | 101.0 | 12.0 |
| Spinosyn A | 14.74 | 30 | 732.4 | 98.2 | 76.00 | 80.0 | 12.0 | 97.0 | 12.5 |
| Spinosyn D | 15.86 | 45 | 746.4 | 99 | 56.29 | 80.0 | 12.0 | 79.0 | 12.5 |
| Spiromesifen | 18.65 | 30 | 371.3 | 273.3 | 10.00 | 30.0 | 12.0 | 13.0 | 20.0 |
| Spiromesifen | 18.65 | 30 | 371.3 | 255.2 | 15.00 | 30.0 | 12.0 | 27.0 | 20.0 |
| Spirotetramat | 12.14 | 30 | 374.2 | 216.1 | 20.00 | 40.0 | 13.0 | 44.0 | 22.5 |
| Spiroxamine | 10.28 | 40 | 298.3 | 144.2 | 19.39 | 50.0 | 13.0 | 26.0 | 12.5 |
| Tebuconazole | 13.64 | 30 | 308.1 | 125 | 39.93 | 50.0 | 12.0 | 53.0 | 10.0 |
| Thiacloprid | 5.23 | 30 | 253.2 | 126 | 10.00 | 40.0 | 13.5 | 26.0 | 15.0 |
| Thiamethoxam | 2.72 | 25 | 292 | 132 | 90.00 | 10.0 | 13.0 | 28.0 | 15.0 |
| Trifloxystrobin | 16.03 | 30 | 409.1 | 186.1 | 47.23 | 30.0 | 12.0 | 23.0 | 10.0 |
| Internal Standards | | | | | | | | | |
| Acephate-d_3_ | 1.39 | 30 | 187 | 143 | 89.18 | 10.0 | 8.0 | 12.0 | 15.0 |
| Acequinocyl-d_25_ | 23.83 | 45 | 427.36 | 368.2 | 250.00 | 50.0 | 6.5 | 16.0 | 20.0 |
| Acetamiprid-d_3_ | 4.43 | 30 | 226.2 | 90 | 55.90 | 40.0 | 13.0 | 46.0 | 10.0 |
| Aldicarb-d_3_ | 5.81 | 30 | 211.1 | 116 | 104.86 | 25.0 | 13.0 | 11.0 | 10.0 |
| (D)-cis,trans-Allethrin | 17.45 | 30 | 303.3 | 135.1 | 67.41 | 25.0 | 12.0 | 14.0 | 15.0 |
| Azoxystrobin-d_4_ | 10.38 | 30 | 408.1 | 348 | 10.00 | 45.0 | 13.0 | 33.0 | 35.0 |
| Bifenazate-d_5_ | 11.71 | 30 | 306.1 | 203.1 | 36.66 | 20.0 | 12.0 | 13.0 | 10.0 |
| Bifenthrin-d_5_ | 21.98 | 30 | 445.1 | 186.1 | 81.34 | 20.0 | 13.0 | 16.0 | 20.0 |
| Boscalid-d_4_ | 10.82 | 30 | 347.3 | 311 | 19.14 | 60.0 | 12.0 | 25.0 | 20.0 |
| Captan-d_6_ | 9.53 | 30 | 322.787 | 270 | 37.35 | 30.0 | 8.0 | 16.0 | 20.0 |
| Carbaryl-d_7_ | 7.72 | 30 | 209.1 | 152 | 66.94 | 20.0 | 13.0 | 15.0 | 15.0 |
| Carbofuran-d_3_ | 7.32 | 30 | 224.896 | 164.9 | 55.81 | 30.0 | 14.0 | 16.0 | 15.0 |
| Chlorantraniliprole-d_3_ | 9.84 | 30 | 486.9 | 452.9 | 24.79 | 35.0 | 12.0 | 24.0 | 35.0 |
| Chlorfenapyr-d_7_ | 16.2 | 45 | 414 | 274.07 | 61.91 | 60.0 | 12.0 | 23.0 | 30.0 |
| Chlorpyrifos-d_10_ | 17.8 | 30 | 360 | 199 | 70.52 | 30.0 | 12.0 | 25.0 | 10.0 |
| Clofentezine-d_8_ | 14.61 | 30 | 311 | 142 | 74.63 | 20.0 | 12.0 | 19.0 | 15.0 |
| Coumaphos-d_10_ | 13.85 | 30 | 373 | 341 | 40.50 | 60.0 | 12.0 | 22.0 | 25.0 |
| Cyfluthrin-d_6_ | 19.35 | 60 | 459 | 198.8 | 41.43 | 20.0 | 12.0 | 19.0 | 10.0 |
| Cypermethrin-d_9_ | 19.65 | 45 | 444 | 192.8 | 41.02 | 25.0 | 12.0 | 20.0 | 10.0 |
| Daminozide-d_4_ | 1.05 | 30 | 165.1 | 147.1 | 117.76 | 25.0 | 9.0 | 15.0 | 15.0 |
| Danitol | 19.13 | 30 | 350.2 | 125.1 | 56.90 | 30.0 | 12.0 | 15.0 | 15.0 |
| Diazinon-d_10_ | 13.92 | 30 | 315.1 | 154 | 40.50 | 60.0 | 12.0 | 29.0 | 15.0 |
| Dichlorvos-d_6_ | 6.94 | 30 | 227 | 115 | 121.38 | 50.0 | 13.0 | 23.0 | 12.5 |
| Dimethoate-d_6_ | 4.22 | 30 | 236 | 205 | 65.88 | 20.0 | 13.0 | 12.0 | 15.0 |
| (E)-Dimethomorph-d_6_ | 10.6 | 30 | 394 | 307 | 18.66 | 75.0 | 14.0 | 28.0 | 25.0 |
| (Z)-Dimethomorph-d_6_ | 11.19 | 30 | 394 | 307.01 | 22.61 | 75.0 | 14.0 | 28.0 | 25.0 |
| Emamectin | 17.63 | 30 | 886.5 | 158.1 | 71.62 | 50.0 | 10.0 | 40.0 | 10.0 |
| Ethoprophos-d_6_ | 12.08 | 30 | 249 | 132 | 55.51 | 35.0 | 14.0 | 27.0 | 15.0 |
| Etofenprox-d_5_ | 21.58 | 30 | 399 | 140.1 | 60.70 | 25.0 | 12.0 | 36.0 | 15.0 |
| Etoxazole-d_5_ | 18.59 | 30 | 365.2 | 141 | 59.71 | 80.0 | 12.0 | 36.0 | 15.0 |
| Fenhexamid-d_3_ | 11.93 | 30 | 305 | 141.8 | 46.39 | 75.0 | 13.0 | 29.0 | 15.0 |
| Fenoxycarb-d_3_ | 13.05 | 30 | 305.2 | 119 | 54.93 | 40.0 | 12.0 | 14.0 | 15.0 |
| Fipronil-^13^C_2_^15^N_2_ | 12.97 | 30 | 441 | 294 | 59.47 | 100.0 | 12.0 | 34.0 | 25.0 |
| Flonicamid-^15^N^18^O | 2.61 | 25 | 233 | 176 | 40.00 | 10.0 | 13.0 | 24.0 | 15.0 |
| Fludioxonil-^13^C_3_ | 10.6 | 30 | 269 | 232 | 18.66 | 15.0 | 13.0 | 14.0 | 15.0 |
| Fludioxonil-^13^C_3_ | 10.6 | 30 | 269 | 160 | 18.66 | 15.0 | 13.0 | 44.0 | 15.0 |
| Hexythiazox-d_11_ | 17.87 | 30 | 364 | 228 | 20.00 | 20.0 | 12.0 | 20.0 | 20.0 |
| Imazalil-d_5_ | 8.29 | 45 | 302.2 | 46.1 | 105.50 | 60.0 | 13.0 | 59.0 | 7.5 |
| Imidacloprid-d_4_ | 3.69 | 30 | 260.1 | 179 | 148.85 | 25.0 | 13.0 | 26.0 | 15.0 |
| Ivermectin-NH_4_ | 22.54 | 30 | 892.65 | 569.3 | 191.85 | 50.0 | 12.0 | 19.0 | 35.0 |
| Ivermectin-Na | 22.53 | 30 | 897.5 | 753.4 | 187.04 | 140.0 | 12.0 | 57.0 | 40.0 |
| Kresoxim Methyl-d_7_ | 13.32 | 30 | 321.1 | 115.9 | 45.93 | 20.0 | 12.0 | 19.0 | 10.0 |
| Malathion-d_6_ | 11.03 | 30 | 337.1 | 291 | 18.53 | 25.0 | 12.0 | 10.0 | 25.0 |
| Metalaxyl-d_3_ | 9.11 | 30 | 283.2 | 223 | 70.86 | 30.0 | 13.0 | 18.0 | 25.0 |
| Methomyl-d_3_ | 2.51 | 25 | 166.1 | 58 | 40.00 | 10.0 | 13.0 | 25.0 | 15.0 |
| Methiocarb-d_3_ | 9.5 | 30 | 229.1 | 169.2 | 38.52 | 20.0 | 8.0 | 11.0 | 10.0 |
| Methyl Parathion-d_6_ | 10.06 | 30 | 270 | 235 | 23.00 | 70.0 | 9.0 | 24.0 | 15.0 |
| (Z)-Mevinphos-d_6_ | 4.38 | 30 | 231 | 132.9 | 56.53 | 25.0 | 13.0 | 23.0 | 15.0 |
| (E)-Mevinphos-d_6_ | 5.39 | 30 | 231.06 | 199.06 | 87.93 | 25.0 | 13.0 | 9.0 | 15.0 |
| Myclobutanil-d_9_ | 11.3 | 30 | 298.1 | 125.2 | 23.66 | 55.0 | 13.0 | 45.0 | 15.0 |
| Naled-d_6_ | 9.29 | 30 | 386.8 | 133.1 | 51.29 | 55.0 | 12.0 | 16.0 | 15.0 |
| Oxamyl-d_6_ | 2.09 | 25 | 243.1 | 78.1 | 95.00 | 10.0 | 13.0 | 28.0 | 15.0 |
| Paclobutrazol-d_4_ | 10.93 | 30 | 298.1 | 70.1 | 18.90 | 45.0 | 13.0 | 23.0 | 10.0 |
| Phosmet-d_6_ | 9.76 | 30 | 324 | 160 | 28.06 | 25.0 | 12.0 | 16.0 | 15.0 |
| (E)-Permethrin-d_5_ | 20.83 | 30 | 413.1 | 188.1 | 59.85 | 25.0 | 12.0 | 23.0 | 20.0 |
| (Z)-Permethrin-d_5_ | 21.4 | 30 | 413.1 | 188.11 | 61.02 | 25.0 | 12.0 | 29.0 | 20.0 |
| Piperonyl Butoxide-d_9_ | 17.29 | 30 | 365.5 | 177 | 30.00 | 20.0 | 12.0 | 16.0 | 12.0 |
| Propoxur-d_3_ | 7.17 | 30 | 212.889 | 93 | 62.81 | 20.0 | 14.0 | 33.0 | 12.5 |
| Propiconazole-d_3_ | 14.01 | 30 | 345.1 | 159 | 41.04 | 50.0 | 6.0 | 37.0 | 15.0 |
| Pyridaben-d_13_ | 19.88 | 30 | 378.1 | 322.2 | 10.00 | 30.0 | 8.0 | 17.0 | 25.0 |
| Spiromesifen NH_4_-d_9_ | 18.52 | 30 | 397.311 | 273.169 | 20.00 | 30.0 | 12.0 | 20.0 | 25.0 |
| Spiromesifen-d_9_ | 18.52 | 30 | 381.247 | 273.209 | 20.00 | 30.0 | 12.0 | 15.0 | 25.0 |
| Spirotetramat-d_5_ | 12.03 | 60 | 379.2 | 335 | 20.00 | 40.0 | 12.5 | 19.0 | 25.0 |
| Spiroxamine-d_4_ | 10.24 | 40 | 302.3 | 144.2 | 19.87 | 50.0 | 12.0 | 26.0 | 12.5 |
| Tebuconazole-d_9_ | 13.49 | 30 | 317.1 | 70.1 | 39.65 | 60.0 | 12.0 | 57.0 | 10.0 |
| Thiacloprid-d_4_ | 5.19 | 30 | 257.2 | 126 | 83.68 | 40.0 | 13.5 | 27.0 | 15.0 |
| Thiamethoxam-d_3_ | 2.7 | 25 | 295 | 184 | 90.00 | 10.0 | 13.0 | 30.0 | 15.0 |
| Trifloxystrobin-d_6_ | 15.89 | 30 | 415.3 | 185.7 | 47.39 | 30.0 | 12.0 | 20.0 | 10.0 |

Supplementary Table 7. Concentrations of Components Corresponding to Each of the 17 Spike-and-Recovery Levels (ng/mL in-vial)

| **Component** | **Spike-and-Recovery Concentrations (ng/mL)** | | | | | | | | | | | | | | | | |
| --- | --- | --- | --- | --- | --- | --- | --- | --- | --- | --- | --- | --- | --- | --- | --- | --- | --- |
|  | **1** | **2** | **3** | **4** | **5** | **6** | **7** | **8** | **9** | **10** | **11** | **12** | **13** | **14** | **15** | **16** | **17** |
| Avermectin B1a | 0.00493 | 0.00985 | 0.0197 | 0.0394 | 0.0788 | 0.158 | 0.315 | 0.63 | 1.26 | 2.52 | 5.04 | 10.1 | 20.2 | 40.4 | 60.5 | 80.7 | 404 |
| Avermectin B1b | 0.00118 | 0.00236 | 0.00471 | 0.00942 | 0.0189 | 0.0377 | 0.0754 | 0.151 | 0.302 | 0.603 | 1.21 | 2.41 | 4.83 | 9.65 | 14.5 | 19.3 | 96.5 |
| Captan | 0.00427 | 0.00855 | 0.0171 | 0.0342 | 0.0684 | 0.137 | 0.273 | 0.547 | 1.09 | 2.19 | 4.38 | 8.75 | 17.5 | 35 | 52.5 | 70 | 350 |
| Cinerin I | 0.00035 | 0.0007 | 0.00139 | 0.00278 | 0.00557 | 0.0111 | 0.0223 | 0.0445 | 0.0891 | 0.178 | 0.356 | 0.713 | 1.43 | 2.85 | 4.28 | 5.7 | 28.5 |
| Cinerin II | 0.00023 | 0.00046 | 0.00093 | 0.00186 | 0.00371 | 0.00742 | 0.0148 | 0.0297 | 0.0594 | 0.119 | 0.238 | 0.475 | 0.95 | 1.9 | 2.85 | 3.8 | 19 |
| Dimethomorph I | 0.00263 | 0.00525 | 0.0105 | 0.021 | 0.042 | 0.084 | 0.168 | 0.336 | 0.672 | 1.34 | 2.69 | 5.38 | 10.8 | 21.5 | 32.3 | 43 | 215 |
| Dimethomorph II | 0.00348 | 0.00696 | 0.0139 | 0.0278 | 0.0557 | 0.111 | 0.223 | 0.445 | 0.891 | 1.78 | 3.56 | 7.13 | 14.3 | 28.5 | 42.8 | 57 | 285 |
| Jasmolin I | 0.00024 | 0.00049 | 0.00098 | 0.00195 | 0.00391 | 0.00781 | 0.0156 | 0.0313 | 0.0625 | 0.125 | 0.25 | 0.5 | 1 | 2 | 3 | 4 | 20 |
| Jasmolin II | 0.00017 | 0.00033 | 0.00066 | 0.00132 | 0.00264 | 0.00527 | 0.0106 | 0.0211 | 0.0422 | 0.0844 | 0.169 | 0.338 | 0.675 | 1.35 | 2.03 | 2.7 | 13.5 |
| Mevinphos I | 0.00067 | 0.00134 | 0.00269 | 0.00537 | 0.0107 | 0.0215 | 0.043 | 0.0859 | 0.172 | 0.344 | 0.688 | 1.38 | 2.75 | 5.5 | 8.25 | 11 | 55 |
| Mevinphos II | 0.00543 | 0.0109 | 0.0217 | 0.0435 | 0.0869 | 0.174 | 0.348 | 0.695 | 1.39 | 2.78 | 5.56 | 11.1 | 22.3 | 44.5 | 66.8 | 89 | 445 |
| Permethrin I | 0.00336 | 0.00671 | 0.0134 | 0.0269 | 0.0537 | 0.107 | 0.215 | 0.43 | 0.859 | 1.72 | 3.44 | 6.88 | 13.8 | 27.5 | 41.3 | 55 | 275 |
| Permethrin II | 0.00275 | 0.00549 | 0.011 | 0.022 | 0.044 | 0.0879 | 0.176 | 0.352 | 0.703 | 1.41 | 2.81 | 5.63 | 11.3 | 22.5 | 33.8 | 45 | 225 |
| Pyrethrin I | 0.00342 | 0.00685 | 0.0137 | 0.0274 | 0.0548 | 0.11 | 0.219 | 0.438 | 0.877 | 1.75 | 3.51 | 7.01 | 14 | 28.1 | 42.1 | 56.1 | 281 |
| Pyrethrin II | 0.0017 | 0.00339 | 0.00679 | 0.0136 | 0.0272 | 0.0543 | 0.109 | 0.217 | 0.434 | 0.869 | 1.74 | 3.48 | 6.95 | 13.9 | 20.9 | 27.8 | 139 |
| Spinetoram J | 0.00437 | 0.00874 | 0.0175 | 0.035 | 0.0699 | 0.14 | 0.28 | 0.559 | 1.12 | 2.24 | 4.48 | 8.95 | 17.9 | 35.8 | 53.7 | 71.6 | 358 |
| Spinetoram L | 0.00173 | 0.00347 | 0.00693 | 0.0139 | 0.0277 | 0.0555 | 0.111 | 0.222 | 0.444 | 0.888 | 1.78 | 3.55 | 7.1 | 14.2 | 21.3 | 28.4 | 142 |
| Spinosyn A | 0.00496 | 0.00991 | 0.0198 | 0.0397 | 0.0793 | 0.159 | 0.317 | 0.634 | 1.27 | 2.54 | 5.08 | 10.2 | 20.3 | 40.6 | 60.9 | 81.2 | 406 |
| Spinosyn D | 0.00103 | 0.00206 | 0.00413 | 0.00825 | 0.0165 | 0.033 | 0.066 | 0.132 | 0.264 | 0.528 | 1.06 | 2.11 | 4.23 | 8.45 | 12.7 | 16.9 | 84.5 |
| All others | 0.006 | 0.012 | 0.024 | 0.049 | 0.098 | 0.195 | 0.391 | 0.781 | 1.563 | 3.125 | 6.25 | 12.50 | 25.00 | 50.00 | 75.00 | 100.0 | 500.0 |

Supplementary Table 8. Concentrations of Components Corresponding to Each of the 17 Spike-and-Recovery Levels (ppb in-sample)

| **Component** | **Spike-and-Recovery Concentrations (ppb)** | | | | | | | | | | | | | | | | |
| --- | --- | --- | --- | --- | --- | --- | --- | --- | --- | --- | --- | --- | --- | --- | --- | --- | --- |
|  | **1** | **2** | **3** | **4** | **5** | **6** | **7** | **8** | **9** | **10** | **11** | **12** | **13** | **14** | **15** | **16** | **17** |
| Avermectin B1a | 0.197 | 0.394 | 0.788 | 1.58 | 3.15 | 6.3 | 12.6 | 25.2 | 50.4 | 101 | 202 | 404 | 807 | 1610 | 2420 | 3230 | 16100 |
| Avermectin B1b | 0.0472 | 0.0944 | 0.188 | 0.377 | 0.754 | 1.51 | 3.02 | 6.03 | 12.1 | 24.1 | 48.3 | 96.5 | 193 | 386 | 579 | 772 | 3860 |
| Captan | 0.171 | 0.342 | 0.684 | 1.37 | 2.73 | 5.47 | 10.9 | 21.9 | 43.8 | 87.5 | 175 | 350 | 700 | 1400 | 2100 | 2800 | 14000 |
| Cinerin I | 0.014 | 0.028 | 0.0556 | 0.111 | 0.223 | 0.445 | 0.891 | 1.78 | 3.56 | 7.13 | 14.3 | 28.5 | 57 | 114 | 171 | 228 | 1140 |
| Cinerin II | 0.0092 | 0.0184 | 0.0372 | 0.0744 | 0.148 | 0.297 | 0.594 | 1.19 | 2.38 | 4.75 | 9.5 | 19 | 38 | 76 | 114 | 152 | 760 |
| Dimethomorph I | 0.105 | 0.21 | 0.42 | 0.84 | 1.68 | 3.36 | 6.72 | 13.4 | 26.9 | 53.8 | 108 | 215 | 430 | 860 | 1290 | 1720 | 8600 |
| Dimethomorph II | 0.139 | 0.278 | 0.557 | 1.11 | 2.23 | 4.45 | 8.91 | 17.8 | 35.6 | 71.3 | 143 | 285 | 570 | 1140 | 1710 | 2280 | 11400 |
| Jasmolin I | 0.0096 | 0.0196 | 0.0392 | 0.078 | 0.156 | 0.312 | 0.625 | 1.25 | 2.5 | 5 | 10 | 20 | 40 | 80 | 120 | 160 | 800 |
| Jasmolin II | 0.0068 | 0.0132 | 0.0264 | 0.0528 | 0.106 | 0.211 | 0.422 | 0.844 | 1.69 | 3.38 | 6.75 | 13.5 | 27 | 54 | 81 | 108 | 540 |
| Mevinphos I | 0.0268 | 0.0536 | 0.108 | 0.215 | 0.43 | 0.859 | 1.72 | 3.44 | 6.88 | 13.8 | 27.5 | 55 | 110 | 220 | 330 | 440 | 2200 |
| Mevinphos II | 0.217 | 0.434 | 0.869 | 1.74 | 3.48 | 6.95 | 13.9 | 27.8 | 55.6 | 111 | 223 | 445 | 890 | 1780 | 2670 | 3560 | 17800 |
| Permethrin I | 0.134 | 0.268 | 0.537 | 1.07 | 2.15 | 4.3 | 8.59 | 17.2 | 34.4 | 68.8 | 138 | 275 | 550 | 1100 | 1650 | 2200 | 11000 |
| Permethrin II | 0.11 | 0.22 | 0.44 | 0.879 | 1.76 | 3.52 | 7.03 | 14.1 | 28.1 | 56.3 | 113 | 225 | 450 | 900 | 1350 | 1800 | 9000 |
| Pyrethrin I | 0.137 | 0.274 | 0.548 | 1.1 | 2.19 | 4.38 | 8.77 | 17.5 | 35.1 | 70.1 | 140 | 281 | 561 | 1120 | 1680 | 2240 | 11200 |
| Pyrethrin II | 0.068 | 0.136 | 0.272 | 0.543 | 1.09 | 2.17 | 4.34 | 8.69 | 17.4 | 34.8 | 69.5 | 139 | 278 | 556 | 834 | 1110 | 5560 |
| Spinetoram J | 0.175 | 0.35 | 0.699 | 1.4 | 2.8 | 5.59 | 11.2 | 22.4 | 44.8 | 89.5 | 179 | 358 | 716 | 1430 | 2150 | 2860 | 14300 |
| Spinetoram L | 0.0692 | 0.139 | 0.277 | 0.555 | 1.11 | 2.22 | 4.44 | 8.88 | 17.8 | 35.5 | 71 | 142 | 284 | 568 | 852 | 1140 | 5680 |
| Spinosyn A | 0.198 | 0.396 | 0.793 | 1.59 | 3.17 | 6.34 | 12.7 | 25.4 | 50.8 | 102 | 203 | 406 | 812 | 1620 | 2440 | 3250 | 16200 |
| Spinosyn D | 0.0412 | 0.0824 | 0.165 | 0.33 | 0.66 | 1.32 | 2.64 | 5.28 | 10.6 | 21.1 | 42.3 | 84.5 | 169 | 338 | 507 | 676 | 3380 |
| All others | 0.244 | 0.488 | 0.976 | 1.95 | 3.91 | 7.81 | 15.6 | 31.3 | 62.5 | 125 | 250 | 500 | 1000 | 2000 | 3000 | 4000 | 20000 |

Supplementary Table 9. 75 Pesticide Components and Internal Standards Used

| **XIC index** | **Compound** | **IS XIC index** | **Internal Standard** | **RRT to IS** | **LogP** | **XLogP** (75) |
| --- | --- | --- | --- | --- | --- | --- |
| 2 | Acephate | 2 | Acephate-d_3_ | 1.01 | -0.85 | -0.8 |
| 75 | Acequinocyl | 65 | Acequinocyl-d_25_ | 1.01 | 6.2 | 7.6 |
| 10 | Acetamiprid | 10 | Acetamiprid-d_3_ | 1.01 | 0.8 | 1.4 |
| 13 | Aldicarb | 13 | Aldicarb-d_3_ | 1.00 | 1.13 | 1.1 |
| 70 | Avermectin B1a | 51 | Emamectin | 1.18 |  | 3.8 |
| 69 | Avermectin B1b | 51 | Emamectin | 1.15 |  | 3.5 |
| 27 | Azoxystrobin | 27 | Azoxystrobin-d_4_ | 1.00 | 2.5 | 3.7 |
| 35 | Bifenazate | 35 | Bifenazate-d_5_ | 1.01 | 3.4 | 4.2 |
| 74 | Bifenthrin | 63 | Bifenthrin-d_5_ | 1.00 | 6 | 6 |
| 30 | Boscalid | 30 | Boscalid-d_4_ | 1.01 | 2.96 | 4.9 |
| 21 | Captan | 22 | Captan-d_6_ | 1.01 | 2.8 | 2.4 |
| 17 | Carbaryl | 17 | Carbaryl-d_7_ | 1.01 | 2.36 | 2.4 |
| 16 | Carbofuran | 16 | Carbofuran-d_3_ | 1.00 | 2.32 | 2.3 |
| 23 | Chlorantraniliprole | 24 | Chlorantraniliprole-d_3_ | 1.00 | 2.76 | 4.8 |
| 54 | Chlorfenapyr | 48 | Chlorfenapyr-d_7_ | 1.01 | 4.83 | 4.5 |
| 58 | Chlorpyrifos | 52 | Chlorpyrifos-d_10_ | 1.01 | 4.96 | 5.3 |
| 62 | Cinerin I | 50 | (D)-cis,trans-Allethrin | 1.08 |  | 5 |
| 49 | Cinerin II | 50 | (D)-cis,trans-Allethrin | 0.90 |  | 4 |
| 47 | Clofentezine | 46 | Clofentezine-d_8_ | 1.01 |  | 3.2 |
| 43 | Coumaphos | 43 | Coumaphos-d_10_ | 1.01 | 4.13 | 4.5 |
| 65 | Cyfluthrin | 57 | Cyfluthrin-d_6_ | 1.01 | 5.95 | 6.2 |
| 66 | Cypermethrin | 58 | Cypermethrin-d_9_ | 1.01 | 6.6 | 6 |
| 1 | Daminozide | 1 | Daminozide-d_4_ | 1.01 | -1.5 | -3.1 |
| 45 | Diazinon | 44 | Diazinon-d_10_ | 1.01 | 3.81 | 3.8 |
| 14 | Dichlorvos | 14 | Dichlorvos-d_6_ | 1.01 | 1.43 | 1.4 |
| 8 | Dimethoate | 8 | Dimethoate-d_6_ | 1.02 | 0.78 | 0.8 |
| 29 | (E)-Dimethomorph | 28 | (E)-Dimethomorph-d_6_ | 1.01 |  | 2.63 |
| 33 | (Z)-Dimethomorph | 33 | (Z)-Dimethomorph-d_6_ | 1.01 |  | 2.73 |
| 61 | Etoxazole | 55 | Etoxazole-d_5_ | 1.00 | 5.59 | 5.4 |
| 38 | Ethoprophos | 38 | Ethoprophos-d_6_ | 1.01 | 3.59 | 3.6 |
| 73 | Etofenprox | 62 | Etofenprox-d_5_ | 1.00 | 6.9 | 7 |
| 36 | Fenhexamid | 36 | Fenhexamid-d_3_ | 1.00 | 3.51 | 4.4 |
| 40 | Fenoxycarb | 40 | Fenoxycarb-d_3_ | 1.00 | 4.3 | 4.3 |
| 64 | Fenpyroximate | 56 | Danitol | 1.00 | 5 | 4.9 |
| 39 | Fipronil | 39 | Fipronil-^13^C_2_^15^N_2_ | 1.00 | 4 | 4.5 |
| 5 | Flonicamid | 5 | Flonicamid-^15^N^18^O | 1.00 | 0.3 | 0.8 |
| 28 | Fludioxonil | 29 | Fludioxonil-^13^C_3_ | 1.00 | 4.12 | 2.6 |
| 59 | Hexythiazox | 53 | Hexythiazox-d_11_ | 1.01 |  | 4.5 |
| 18 | Imazalil | 18 | Imazalil-d_5_ | 1.01 | 3.82 | 3.8 |
| 7 | Imidacloprid | 7 | Imidacloprid-d_4_ | 1.01 | 0.57 | 1.2 |
| 64 | Ivermectin | N/A | N/A | N/A |  | 2.84 |
| 68 | Jasmolin I | 62 | Etofenprox-d_5_ | 0.93 |  | 5.4 |
| 56 | Jasmolin II | 50 | (D)-cis,trans-Allethrin | 0.98 |  | 4.5 |
| 41 | Kresoxim Methyl | 41 | Kresoxim Methyl-d_7_ | 1.01 | 3.4 | 4.1 |
| 32 | Malathion | 32 | Malathion-d_6_ | 1.01 | 2.36 | 2.4 |
| 19 | Metalaxyl | 19 | Metalaxyl-d_3_ | 1.00 | 1.65 | 1.6 |
| 26 | Methiocarb | 21 | Methiocarb-d_3_ | 1.10 | 2.92 | 2.9 |
| 4 | Methomyl | 4 | Methomyl-d_3_ | 1.01 | 0.6 | 0.6 |
| 24 | Methyl Parathion | 25 | Methyl Parathion-d_6_ | 1.01 | 2.86 | 2.9 |
| 12 | (E)-Mevinphos | 9 | (E)-Mevinphos-d_6_ | 1.01 |  | 1.2 |
| 9 | (Z)-Mevinphos | 12 | (Z)-Mevinphos-d_6_ | 1.01 |  | 1.2 |
| 34 | Myclobutanil | 34 | Myclobutanil-d_9_ | 1.01 | 2.94 | 2.9 |
| 20 | Naled | 20 | Naled-d_6_ | 1.01 | 1.38 | 2.5 |
| 3 | Oxamyl | 3 | Oxamyl-d_6_ | 1.02 | -0.47 | -0.5 |
| 31 | Paclobutrazol | 31 | Paclobutrazol-d_4_ | 1.00 |  | 3.2 |
| 71 | (E)-Permethrin | 60 | (E)-Permethrin-d_5_ | 1.00 |  | 6.5 |
| 72 | (Z)-Permethrin | 61 | (Z)-Permethrin-d_5_ | 1.00 |  | 6.5 |
| 22 | Phosmet | 23 | Phosmet-d_6_ | 1.01 | 2.95 | 2.8 |
| 57 | Piperonyl Butoxide | 49 | Piperonyl Butoxide-d_9_ | 1.01 | 4.75 | 3.7 |
| 48 | Prallethrin | 50 | (D)-cis,trans-Allethrin | 0.89 | 4.49 | 4.3 |
| 44 | Propiconazole | 45 | Propiconazole-d_3_ | 1.00 | 3.72 | 3.5 |
| 15 | Propoxur | 15 | Propoxur-d_3_ | 1.00 | 1.52 | 1.5 |
| 63 | Pyrethrin I | 50 | (D)-cis,trans-Allethrin | 1.09 | 5.9 | 5.4 |
| 51 | Pyrethrin II | 50 | (D)-cis,trans-Allethrin | 0.91 | 4.3 | 4.4 |
| 67 | Pyridaben | 59 | Pyridaben-d_13_ | 1.01 | 6.37 | 5.3 |
| 52 | Spinetoram J | 56 | Danitol | 0.83 |  | 5.9 |
| 55 | Spinetoram L | 56 | Danitol | 0.89 |  | 5.2 |
| 46 | Spinosyn A | 56 | Danitol | 0.77 | 2.8 | 4.9 |
| 50 | Spinosyn D | 65 | Acequinocyl-d_25_ | 0.66 |  | 4.8 |
| 60 | Spiromesifen | 54 | Spiromesifen-d_9_ | 1.00 | 4.55 | 5.1 |
| 37 | Spirotetramat | 37 | Spirotetramat-d_5_ | 1.01 |  | 3.2 |
| 25 | Spiroxamine | 26 | Spiroxamine-d_4_ | 1.00 |  | 4.2 |
| 42 | Tebuconazole | 42 | Tebuconazole-d_9_ | 1.01 | 3.7 | 3.7 |
| 11 | Thiacloprid | 11 | Thiacloprid-d_4_ | 1.01 | 1.26 | 2.2 |
| 6 | Thiamethoxam | 6 | Thiamethoxam-d_3_ | 1.01 | -0.13 | 1.5 |
| 53 | Trifloxystrobin | 47 | Trifloxystrobin-d_6_ | 1.01 | 4.5 | 4.9 |

Supplementary Table 10. Analyte AMRs

| **Analyte** | **# of Calibrators** | **R^2^** | **ng/mL in-vial** | | | **ppb in-sample** | | |
| --- | --- | --- | --- | --- | --- | --- | --- | --- |
|  |  |  | **LLOQ** | **ULOQ** | **Extended ULOQ** | **LLOQ** | **ULOQ** | **Extended ULOQ** |
| Acephate | 16 | 0.9989 | 0.0244 | 100 | 2000 | 0.976 | 4000 | 80000 |
| Acequinocyl | 13 | 0.9975 | 0.195 | 100 | 2000 | 7.81 | 4000 | 80000 |
| Acetamiprid | 16 | 0.9975 | 0.0244 | 100 | N/A | 0.976 | 4000 | N/A |
| Aldicarb | 15 | 0.9987 | 0.0488 | 100 | 2000 | 1.95 | 4000 | 80000 |
| Avermectin B1a | 9 | 0.9972 | 1.26 | 60.525 | 605.25 | 50.4 | 2421 | N/A |
| Avermectin B1b | 7 | 0.9956 | 2.41 | 19.3 | N/A | 96.5 | 772 | N/A |
| Azoxystrobin | 17 | 0.9958 | 0.0122 | 100 | 2000 | 0.488 | 4000 | 80000 |
| Bifenazate | 12 | 0.9985 | 0.391 | 100 | 2000 | 15.6 | 4000 | 80000 |
| Bifenthrin | 12 | 0.9985 | 0.391 | 100 | N/A | 15.6 | 4000 | N/A |
| Boscalid | 15 | 0.9986 | 0.0488 | 100 | 2000 | 1.95 | 4000 | 80000 |
| Captan | 8 | 0.9972 | 6.25 | 100 | 1000 | 250 | 4000 | 40000 |
| Carbaryl | 16 | 0.9982 | 0.0244 | 100 | 2000 | 0.976 | 4000 | 80000 |
| Carbofuran | 13 | 0.9983 | 0.0488 | 75 | 1500 | 1.95 | 3000 | 60000 |
| Chlorantraniliprole | 14 | 0.9987 | 0.0977 | 100 | 2000 | 3.91 | 4000 | 80000 |
| Chlorfenapyr | 9 | 0.9958 | 3.13 | 100 | 1000 | 125 | 4000 | 40000 |
| Chlorpyrifos | 15 | 0.9989 | 0.0488 | 100 | 2000 | 1.95 | 4000 | 80000 |
| Cinerin I | 12 | 0.9976 | 0.0223 | 5.7 | N/A | 0.891 | 228 | N/A |
| Cinerin II | 11 | 0.9980 | 0.0297 | 3.8 | N/A | 1.19 | 152 | N/A |
| Clofentezine | 16 | 0.9986 | 0.0244 | 100 | 2000 | 0.976 | 4000 | 80000 |
| Coumaphos | 15 | 0.9984 | 0.0488 | 100 | 2000 | 1.95 | 4000 | 80000 |
| Cyfluthrin | 9 | 0.9976 | 3.13 | 100 | 2000 | 125 | 4000 | 40000 |
| Cypermethrin | 10 | 0.9979 | 1.56 | 100 | 2000 | 62.5 | 4000 | 80000 |
| Daminozide | 14 | 0.9984 | 0.0977 | 100 | 2000 | 3.91 | 4000 | 80000 |
| Diazinon | 17 | 0.9986 | 0.0122 | 100 | 2000 | 0.488 | 4000 | 80000 |
| Dichlorvos | 13 | 0.9985 | 0.195 | 100 | 2000 | 7.81 | 4000 | 80000 |
| Dimethoate | 16 | 0.9980 | 0.0244 | 100 | 2000 | 0.976 | 4000 | 80000 |
| (E)-Dimethomorph | 13 | 0.9984 | 0.0840 | 43 | 860 | 3.36 | 1720 | 34400 |
| (Z)-Dimethomorph | 14 | 0.9987 | 0.0557 | 57 | 1140 | 2.23 | 2280 | 45600 |
| Ethoprophos | 17 | 0.9981 | 0.0122 | 100 | 2000 | 0.488 | 4000 | 80000 |
| Etofenprox | 14 | 0.9982 | 0.0977 | 100 | 2000 | 3.91 | 4000 | 80000 |
| Etoxazole | 12 | 0.9985 | 0.0977 | 50 | 1000 | 3.91 | 2000 | 40000 |
| Fenhexamid | 10 | 0.9986 | 0.391 | 50 | N/A | 15.6 | 2000 | N/A |
| Fenoxycarb | 15 | 0.9986 | 0.0488 | 100 | 2000 | 1.95 | 4000 | 80000 |
| Fenpyroximate | 13 | 0.9982 | 0.0977 | 75 | 1500 | 3.91 | 3000 | 60000 |
| Fipronil | 13 | 0.9970 | 0.0977 | 75 | N/A | 3.91 | 3000 | N/A |
| Flonicamid | 16 | 0.9980 | 0.0244 | 100 | 2000 | 0.976 | 4000 | 80000 |
| Fludioxonil | 13 | 0.9985 | 0.195 | 100 | 2000 | 7.81 | 4000 | 80000 |
| Hexythiazox | 14 | 0.9983 | 0.0488 | 75 | 1500 | 1.95 | 3000 | 60000 |
| Imazalil | 18 | 0.9983 | 0.00610 | 100 | 2000 | 0.244 | 4000 | 80000 |
| Imidacloprid | 18 | 0.9985 | 0.00610 | 100 | 2000 | 0.244 | 4000 | 80000 |
| Jasmolin I | 9 | 0.9974 | 0.0625 | 3 | N/A | 2.50 | 120 | N/A |
| Jasmolin II | 9 | 0.9973 | 0.0844 | 2.7 | N/A | 3.38 | 108 | N/A |
| Kresoxim Methyl | 14 | 0.9969 | 0.0977 | 100 | 2000 | 3.91 | 4000 | 80000 |
| Malathion | 17 | 0.9984 | 0.0122 | 100 | 2000 | 0.488 | 4000 | 80000 |
| Metalaxyl | 15 | 0.9951 | 0.0488 | 100 | 2000 | 1.95 | 4000 | 80000 |
| Methiocarb | 13 | 0.9962 | 0.0977 | 75 | 1500 | 3.91 | 3000 | 60000 |
| Methomyl | 15 | 0.9975 | 0.0488 | 100 | 2000 | 1.95 | 4000 | 80000 |
| Methyl Parathion | 10 | 0.9977 | 1.560 | 100 | 2000 | 62.5 | 4000 | 80000 |
| (Z)-Mevinphos | 14 | 0.9981 | 0.0107 | 11 | 220 | 0.430 | 440 | 8800 |
| (E)-Mevinphos | 18 | 0.9986 | 0.00543 | 89 | 1780 | 0.217 | 3560 | 71200 |
| Myclobutanil | 13 | 0.9976 | 0.0977 | 75 | 1500 | 3.91 | 3000 | 60000 |
| Naled | 12 | 0.9988 | 0.391 | 100 | 2000 | 15.6 | 4000 | 80000 |
| Oxamyl | 16 | 0.9981 | 0.0244 | 100 | 2000 | 0.976 | 4000 | 80000 |
| Paclobutrazol | 13 | 0.9984 | 0.195 | 100 | 2000 | 7.81 | 4000 | 80000 |
| (E)-Permethrin | 13 | 0.9991 | 0.107 | 55 | 1100 | 4.30 | 2200 | 44000 |
| (Z)-Permethrin | 11 | 0.9989 | 0.352 | 45 | 900 | 14.1 | 1800 | 36000 |
| Phosmet | 12 | 0.9985 | 0.391 | 100 | 2000 | 15.6 | 4000 | 80000 |
| Piperonyl Butoxide | 15 | 0.9980 | 0.0488 | 100 | 2000 | 1.95 | 4000 | 80000 |
| Prallethrin | 12 | 0.9984 | 0.391 | 100 | N/A | 15.6 | 4000 | N/A |
| Propiconazole | 13 | 0.9985 | 0.195 | 100 | 2000 | 7.81 | 4000 | 80000 |
| Propoxur | 14 | 0.9988 | 0.0977 | 100 | 2000 | 3.91 | 4000 | 80000 |
| Pyrethrin I | 12 | 0.9985 | 0.219 | 56.1 | N/A | 8.77 | 2244 | N/A |
| Pyrethrin II | 11 | 0.9978 | 0.217 | 27.8 | N/A | 8.69 | 1112 | N/A |
| Pyridaben | 9 | 0.9974 | 0.781 | 50 | 1000 | 31.3 | 2000 | 40000 |
| Spinetoram J | 15 | 0.9989 | 0.0350 | 71.6 | 1432 | 1.40 | 2864 | 57280 |
| Spinetoram L | 13 | 0.9984 | 0.0555 | 28.4 | 568 | 2.22 | 1136 | 22720 |
| Spinosyn A | 13 | 0.9983 | 0.0793 | 60.9 | 1218 | 3.17 | 2436 | 48720 |
| Spinosyn D | 11 | 0.9981 | 0.132 | 16.9 | N/A | 5.28 | 676 | N/A |
| Spiromesifen | 12 | 0.9965 | 0.0977 | 50 | 1000 | 3.91 | 2000 | 40000 |
| Spirotetramat | 17 | 0.9965 | 0.00610 | 75 | 1500 | 0.244 | 3000 | 60000 |
| Spiroxamine | 16 | 0.9983 | 0.0244 | 100 | 2000 | 0.976 | 4000 | 80000 |
| Tebuconazole | 13 | 0.9985 | 0.195 | 100 | 2000 | 7.81 | 4000 | 80000 |
| Thiacloprid | 17 | 0.9971 | 0.0122 | 100 | 2000 | 0.488 | 4000 | 80000 |
| Thiamethoxam | 16 | 0.9973 | 0.0244 | 100 | 2000 | 0.976 | 4000 | 80000 |
| Trifloxystrobin | 13 | 0.9981 | 0.195 | 100 | 2000 | 7.81 | 4000 | 80000 |

Supplementary Table 11. Surrogate IS Analysis - Maximum Absolute Bias across 4 Analytical Batches

| See supplementary table 11 | (Excel file) |
| --- | --- |

Supplementary Table 12. Pesticide Action Limits in the State of California (Inhalable Products)

| **Compound** | **Category** | **Action limit** | | |
| --- | --- | --- | --- | --- |
|  |  | **µg/g** | **ppb in-sample** | **ng/mL in-vial** |
| Avermectin B1a | II | 0.1 | 100 | 2.5 |
| Avermectin B1b | II | 0.1 | 100 | 2.5 |
| Acephate | II | 0.1 | 100 | 2.5 |
| Acequinocyl | II | 0.1 | 100 | 2.5 |
| Acetamiprid | II | 0.1 | 100 | 2.5 |
| Aldicarb | I | 0.1 | 100 | 2.5 |
| Azoxystrobin | II | 0.1 | 100 | 2.5 |
| Bifenazate | II | 0.1 | 100 | 2.5 |
| Bifenthrin | II | 3 | 3000 | 75 |
| Boscalid | II | 0.1 | 100 | 2.5 |
| Captan | II | 0.7 | 700 | 17.5 |
| Carbaryl | II | 0.5 | 500 | 12.5 |
| Carbofuran | I | 0.1 | 100 | 2.5 |
| Chlorantraniliprole | II | 10 | 10000 | 250 |
| Chlordane | I | 0.1 | 100 | 2.5 |
| Chlorfenapyr | I | 0.1 | 100 | 2.5 |
| Chlorpyrifos | I | 0.1 | 100 | 2.5 |
| Clofentezine | II | 0.1 | 100 | 2.5 |
| Coumaphos | I | 0.1 | 100 | 2.5 |
| Cyfluthrin | II | 2 | 2000 | 50 |
| Cypermethrin | II | 1 | 1000 | 25 |
| Daminozide | I | 0.1 | 100 | 2.5 |
| Dichlorvos | I | 0.1 | 100 | 2.5 |
| Diazinon | II | 0.1 | 100 | 2.5 |
| Dimethoate | II | 0.1 | 100 | 2.5 |
| (E)-Dimethomorph | II | 2 | 2000 | 50 |
| (Z)-Dimethomorph | II | 2 | 2000 | 50 |
| Ethoprophos | I | 0.1 | 100 | 2.5 |
| Etofenprox | I | 0.1 | 100 | 2.5 |
| Etoxazole | II | 0.1 | 100 | 2.5 |
| Fenhexamid | II | 0.1 | 100 | 2.5 |
| Fenoxycarb | I | 0.1 | 100 | 2.5 |
| Fenpyroximate | II | 0.1 | 100 | 2.5 |
| Fipronil | I | 0.1 | 100 | 2.5 |
| Flonicamid | II | 0.1 | 100 | 2.5 |
| Fludioxonil | II | 0.1 | 100 | 2.5 |
| Hexythiazox | II | 0.1 | 100 | 2.5 |
| Imazalil | I | 0.1 | 100 | 2.5 |
| Imidacloprid | II | 5 | 5000 | 125 |
| Kresoxim Methyl | II | 0.1 | 100 | 2.5 |
| Malathion | II | 0.5 | 500 | 12.5 |
| Metalaxyl | II | 2 | 2000 | 50 |
| Methiocarb | I | 0.1 | 100 | 2.5 |
| Methomyl | II | 1 | 1000 | 25 |
| Methyl Parathion | I | 0.1 | 100 | 2.5 |
| (Z)-Mevinphos | I | 0.1 | 100 | 2.5 |
| (E)-Mevinphos | I | 0.1 | 100 | 2.5 |
| Myclobutanil | II | 0.1 | 100 | 2.5 |
| Naled | II | 0.1 | 100 | 2.5 |
| Oxamyl | II | 0.5 | 500 | 12.5 |
| Paclobutrazol | I | 0.1 | 100 | 2.5 |
| Pentachloronitrobenzene | II | 0.1 | 100 | 2.5 |
| (E)-Permethrin | II | 0.5 | 500 | 12.5 |
| (Z)-Permethrin | II | 0.5 | 500 | 12.5 |
| Phosmet | II | 0.1 | 100 | 2.5 |
| Piperonyl Butoxide | II | 3 | 3000 | 75 |
| Prallethrin | II | 0.1 | 100 | 2.5 |
| Propiconazole | II | 0.1 | 100 | 2.5 |
| Propoxur | I | 0.1 | 100 | 2.5 |
| Cinerin I | II | 0.5 | 500 | 12.5 |
| Cinerin II | II | 0.5 | 500 | 12.5 |
| Jasmolin I | II | 0.5 | 500 | 12.5 |
| Jasmolin II | II | 0.5 | 500 | 12.5 |
| Pyrethrin I | II | 0.5 | 500 | 12.5 |
| Pyrethrin II | II | 0.5 | 500 | 12.5 |
| Pyridaben | II | 0.1 | 100 | 2.5 |
| Spinetoram J | II | 0.1 | 100 | 2.5 |
| Spinetoram L | II | 0.1 | 100 | 2.5 |
| Spinosyn A | II | 0.1 | 100 | 2.5 |
| Spinosyn D | II | 0.1 | 100 | 2.5 |
| Spiromesifen | II | 0.1 | 100 | 2.5 |
| Spirotetramat | II | 0.1 | 100 | 2.5 |
| Spiroxamine | I | 0.1 | 100 | 2.5 |
| Tebuconazole | II | 0.1 | 100 | 2.5 |
| Thiacloprid | I | 0.1 | 100 | 2.5 |
| Thiamethoxam | II | 5 | 5000 | 125 |
| Trifloxystrobin | II | 0.1 | 100 | 2.5 |

Supplementary Table 13. Inter-day Accuracy (% Recovery) (n= 7 at each level assessed)

| **Compound** | **Levels Assessed**  **Within primary AMR** | **Average % Recovery across AMR** | **Average % Recovery at Level Assessed (n = 7)** | | | | | | | | | | | | | | | | | | | | | | | | | | | | | |
| --- | --- | --- | --- | --- | --- | --- | --- | --- | --- | --- | --- | --- | --- | --- | --- | --- | --- | --- | --- | --- | --- | --- | --- | --- | --- | --- | --- | --- | --- | --- | --- | --- |
|  |  |  | **1** | | **2** | | **3** | | **4** | | **5** | | **6** | | **7** | | **8** | | **9** | | **10** | | **11** | | **12** | | **13** | | **14** | **15** | **16** | **17*** |
| Acephate | 14 | 104 | N/A | N/A | | 114.9 | | 109.2 | | 108.9 | | 105.5 | | 103.9 | | 101.3 | | 102.0 | | 101.5 | | 103.2 | | 102.2 | | 101.0 | | 99.8 | | 101.0 | 101.9 | 99.8 |
| Acequinocyl | 11 | 91.3 | N/A | N/A | | N/A | | N/A | | N/A | | 90.7 | | 88.5 | | 90.5 | | 91.6 | | 89.4 | | 91.8 | | 90.8 | | 92.9 | | 94.1 | | 90.9 | 93.1 | 97.9 |
| Acetamiprid | 14 | 103.6 | N/A | N/A | | 97.5 | | 101.0 | | 104.9 | | 105.7 | | 108.1 | | 107.7 | | 108.5 | | 105.8 | | 106.9 | | 105.8 | | 103.9 | | 101.3 | | 98.0 | 95.2 | N/A |
| Aldicarb | 13 | 99.4 | N/A | N/A | | N/A | | 100.7 | | 107.6 | | 100.0 | | 101.8 | | 97.6 | | 100.7 | | 96.6 | | 97.6 | | 97.4 | | 99.9 | | 98.3 | | 97.8 | 95.9 | 97.4 |
| Avermectin B1a | 7 | 111.6 | N/A | N/A | | N/A | | N/A | | N/A | | N/A | | N/A | | N/A | | 111.2 | | 119.2 | | 103.6 | | 100.7 | | 116.0 | | 116.4 | | 114.2 | N/A | 96.6 |
| Avermectin B1b | 5 | 104.7 | N/A | N/A | | N/A | | N/A | | N/A | | N/A | | N/A | | N/A | | N/A | | N/A | | N/A | | 90.5 | | 110.0 | | 111.0 | | 104.1 | 107.8 | N/A |
| Azoxystrobin | 15 | 105.2 | N/A | 98.1 | | 105.7 | | 108.7 | | 110.0 | | 107.7 | | 108.2 | | 108.3 | | 108.7 | | 108.3 | | 108.0 | | 107.8 | | 105.6 | | 102.2 | | 97.1 | 93.2 | 97.2 |
| Bifenazate | 10 | 116.8 | N/A | N/A | | N/A | | N/A | | N/A | | N/A | | 116.8 | | 115.7 | | 118.6 | | 119.6 | | 119.7 | | 116.7 | | 116.7 | | 115.3 | | 115.3 | 113.9 | 108.3 |
| Bifenthrin | 10 | 101.7 | N/A | N/A | | N/A | | N/A | | N/A | | N/A | | 102.0 | | 95.4 | | 98.8 | | 97.7 | | 103.1 | | 107.7 | | 100.0 | | 98.5 | | 104.7 | 109.1 | N/A |
| Boscalid | 13 | 112.5 | N/A | N/A | | N/A | | 115.6 | | 114.0 | | 111.0 | | 112.4 | | 117.2 | | 115.2 | | 114.0 | | 112.9 | | 111.1 | | 113.2 | | 113.1 | | 109.0 | 103.3 | 93.5 |
| Captan | 5 | 110.6 | N/A | N/A | | N/A | | N/A | | N/A | | N/A | | N/A | | N/A | | N/A | | N/A | | N/A | | 103.4 | | 107.5 | | 111.9 | | 116.8 | 113.4 | 114.4 |
| Carbaryl | 14 | 100 | N/A | N/A | | 113.7 | | 105.8 | | 104.7 | | 101.5 | | 100.6 | | 97.8 | | 98.2 | | 97.9 | | 98.4 | | 98.7 | | 97.0 | | 95.9 | | 94.7 | 94.6 | 95.9 |
| Carbofuran | 12 | 104.6 | N/A | N/A | | N/A | | 119.8 | | 112.6 | | 105.5 | | 102.8 | | 103.1 | | 102.6 | | 101.4 | | 100.6 | | 100.5 | | 101.8 | | 103.3 | | 101.1 | N/A | 99.8 |
| Chlorantraniliprole | 12 | 102.2 | N/A | N/A | | N/A | | N/A | | 113.8 | | 108.9 | | 104.4 | | 98.7 | | 100.7 | | 98.9 | | 100.5 | | 102.0 | | 100.1 | | 98.8 | | 98.1 | 100.9 | 104.1 |
| Chlorfenapyr | 7 | 102.9 | N/A | N/A | | N/A | | N/A | | N/A | | N/A | | N/A | | N/A | | N/A | | 103.4 | | 105.0 | | 98.6 | | 105.5 | | 108.8 | | 104.2 | 95.0 | 96.9 |
| Chlorpyrifos | 13 | 92.2 | N/A | N/A | | N/A | | 87.8 | | 92.1 | | 91.2 | | 93.2 | | 92.4 | | 93.5 | | 92.5 | | 92.6 | | 93.6 | | 93.4 | | 93.3 | | 91.5 | 91.9 | 90.5 |
| Cinerin I | 10 | 91.5 | N/A | N/A | | N/A | | N/A | | N/A | | N/A | | 98.2 | | 94.1 | | 91.5 | | 90.7 | | 91.0 | | 90.1 | | 90.7 | | 91.7 | | 88.6 | 88.2 | N/A |
| Cinerin II | 9 | 98.7 | N/A | N/A | | N/A | | N/A | | N/A | | N/A | | N/A | | 112.0 | | 110.3 | | 100.7 | | 95.2 | | 93.9 | | 95.3 | | 96.1 | | 92.6 | 91.9 | N/A |
| Clofentezine | 14 | 99.9 | N/A | N/A | | 100.6 | | 100.3 | | 102.1 | | 100.3 | | 100.4 | | 97.0 | | 99.1 | | 97.8 | | 99.3 | | 101.5 | | 99.6 | | 99.6 | | 99.2 | 101.6 | 101.9 |
| Coumaphos | 13 | 90 | N/A | N/A | | N/A | | 100.6 | | 95.1 | | 94.1 | | 89.9 | | 87.2 | | 87.8 | | 87.0 | | 87.8 | | 88.5 | | 87.9 | | 87.9 | | 86.7 | 89.1 | 88.0 |
| Cyfluthrin | 7 | 109.5 | N/A | N/A | | N/A | | N/A | | N/A | | N/A | | N/A | | N/A | | N/A | | 113.7 | | 110.5 | | 112.5 | | 107.8 | | 105.6 | | 106.8 | 109.7 | 107.8 |
| Cypermethrin | 8 | 109.6 | N/A | N/A | | N/A | | N/A | | N/A | | N/A | | N/A | | N/A | | 106.4 | | 109.4 | | 111.1 | | 112.2 | | 110.4 | | 109.6 | | 107.6 | 109.7 | 105.6 |
| Daminozide | 12 | 89.7 | N/A | N/A | | N/A | | N/A | | 93.6 | | 81.7 | | 81.6 | | 81.6 | | 84.3 | | 88.6 | | 91.8 | | 94.1 | | 95.6 | | 95.0 | | 95.0 | 94.0 | 89.7 |
| Diazinon | 15 | 96.9 | N/A | 84.6 | | 92.2 | | 96.2 | | 100.2 | | 100.3 | | 99.7 | | 95.7 | | 98.2 | | 97.6 | | 98.9 | | 99.1 | | 98.3 | | 97.3 | | 97.2 | 98.7 | 99.0 |
| Dichlorvos | 11 | 103.2 | N/A | N/A | | N/A | | N/A | | N/A | | 116.6 | | 109.3 | | 104.2 | | 102.9 | | 101.0 | | 100.8 | | 99.8 | | 101.1 | | 100.8 | | 99.7 | 99.0 | 99.5 |
| Dimethoate | 14 | 100.6 | N/A | N/A | | 94.0 | | 108.2 | | 106.0 | | 104.4 | | 106.6 | | 103.2 | | 105.1 | | 103.6 | | 104.0 | | 102.3 | | 99.2 | | 94.2 | | 89.5 | 87.5 | 87.0 |
| (E)-Dimethomorph | 11 | 104.5 | N/A | N/A | | N/A | | N/A | | N/A | | 117.3 | | 109.8 | | 103.3 | | 103.7 | | 102.6 | | 103.7 | | 105.1 | | 102.2 | | 101.6 | | 98.7 | 101.4 | 100.6 |
| (Z)-Dimethomorph | 12 | 106.7 | N/A | N/A | | N/A | | N/A | | 118.1 | | 116.8 | | 111.4 | | 104.6 | | 106.0 | | 106.0 | | 107.1 | | 108.1 | | 104.2 | | 100.3 | | 98.4 | 99.1 | 100.8 |
| Ethoprophos | 15 | 103.2 | N/A | 96.7 | | 103.0 | | 103.4 | | 106.9 | | 104.5 | | 105.2 | | 103.7 | | 105.1 | | 104.5 | | 105.3 | | 104.9 | | 104.3 | | 102.2 | | 99.6 | 98.3 | 101.1 |
| Etofenprox | 12 | 97.7 | N/A | N/A | | N/A | | N/A | | 83.9 | | 94.7 | | 97.6 | | 98.7 | | 102.2 | | 100.8 | | 100.8 | | 101.0 | | 99.7 | | 99.1 | | 97.2 | 97.1 | 95.8 |
| Etoxazole | 11 | 90.4 | N/A | N/A | | N/A | | N/A | | 83.4 | | 88.1 | | 90.8 | | 90.4 | | 92.0 | | 91.9 | | 92.3 | | 92.7 | | 92.2 | | 91.7 | | N/A | N/A | 87.7 |
| Fenhexamid | 9 | 102 | N/A | N/A | | N/A | | N/A | | N/A | | N/A | | 98.8 | | 101.9 | | 106.3 | | 107.5 | | 108.3 | | 106.3 | | 103.1 | | 95.6 | | N/A | N/A | N/A |
| Fenoxycarb | 13 | 98.3 | N/A | N/A | | N/A | | 115.5 | | 107.2 | | 99.3 | | 98.2 | | 95.5 | | 96.0 | | 95.1 | | 95.7 | | 95.6 | | 95.6 | | 95.9 | | 93.8 | 94.6 | 93.8 |
| Fenpyroximate | 11 | 111.2 | N/A | N/A | | N/A | | N/A | | 96.3 | | 109.6 | | 109.7 | | 113.9 | | 116.7 | | 116.7 | | 115.5 | | 115.2 | | 114.0 | | 111.8 | | 103.6 | N/A | 111.1 |
| Fipronil | 11 | 103.3 | N/A | N/A | | N/A | | N/A | | 99.7 | | 104.1 | | 105.2 | | 104.3 | | 107.7 | | 105.0 | | 104.8 | | 103.5 | | 104.8 | | 100.9 | | 96.6 | N/A | N/A |
| Flonicamid | 14 | 97.8 | N/A | N/A | | 113.9 | | 97.7 | | 100.2 | | 98.2 | | 97.3 | | 99.6 | | 97.5 | | 96.9 | | 97.2 | | 94.1 | | 96.6 | | 95.8 | | 92.4 | 91.4 | 91.9 |
| Fludioxonil | 11 | 101.1 | N/A | N/A | | N/A | | N/A | | N/A | | 118.6 | | 104.0 | | 103.0 | | 100.5 | | 96.6 | | 99.7 | | 100.2 | | 98.1 | | 98.2 | | 96.3 | 97.3 | 95.5 |
| Hexythiazox | 12 | 92.9 | N/A | N/A | | N/A | | 86.3 | | 98.5 | | 101.4 | | 97.8 | | 86.8 | | 90.9 | | 90.8 | | 94.4 | | 98.1 | | 91.4 | | 88.4 | | 89.9 | N/A | 116.6 |
| Imazalil | 16 | 98.8 | 108.3 | 104.2 | | 92.9 | | 100.0 | | 102.2 | | 98.2 | | 100.4 | | 98.4 | | 99.0 | | 98.3 | | 98.2 | | 98.4 | | 98.2 | | 96.7 | | 94.4 | 93.7 | 95.2 |
| Imidacloprid | 16 | 104.7 | 102.2 | 107.2 | | 108.3 | | 109.0 | | 109.2 | | 108.8 | | 107.2 | | 105.2 | | 106.6 | | 104.8 | | 105.2 | | 104.4 | | 102.7 | | 100.1 | | 97.9 | 96.7 | 97.1 |
| Jasmolin I | 7 | 100.5 | N/A | N/A | | N/A | | N/A | | N/A | | N/A | | N/A | | N/A | | 108.8 | | 105.5 | | 100.7 | | 97.2 | | 97.9 | | 97.8 | | 95.9 | N/A | N/A |
| Jasmolin II | 7 | 103.1 | N/A | N/A | | N/A | | N/A | | N/A | | N/A | | N/A | | N/A | | N/A | | 112.5 | | 104.2 | | 96.3 | | 105.4 | | 110.0 | | 102.0 | 91.2 | N/A |
| Kresoxim-methyl | 12 | 105.4 | N/A | N/A | | N/A | | N/A | | 109.6 | | 99.4 | | 104.9 | | 106.5 | | 105.3 | | 106.2 | | 108.2 | | 105.4 | | 108.4 | | 107.2 | | 102.2 | 101.7 | 105.1 |
| Malathion | 15 | 102.9 | N/A | 101.8 | | 103.3 | | 104.4 | | 106.6 | | 103.1 | | 105.1 | | 101.6 | | 103.5 | | 102.9 | | 102.8 | | 103.9 | | 103.1 | | 101.7 | | 99.6 | 99.9 | 97.7 |
| Metalaxyl | 13 | 108.2 | N/A | N/A | | N/A | | 103.4 | | 106.3 | | 110.2 | | 111.0 | | 110.6 | | 111.7 | | 112.2 | | 111.1 | | 112.1 | | 110.3 | | 107.3 | | 101.8 | 98.2 | 102.7 |
| Methiocarb | 11 | 100 | N/A | N/A | | N/A | | N/A | | 91.5 | | 98.2 | | 102.7 | | 104.0 | | 105.7 | | 103.9 | | 104.1 | | 103.3 | | 100.7 | | 95.4 | | 90.4 | N/A | 93.9 |
| Methomyl | 13 | 102.3 | N/A | N/A | | N/A | | 100.6 | | 110.1 | | 105.5 | | 103.4 | | 100.0 | | 102.8 | | 102.5 | | 101.9 | | 101.3 | | 102.0 | | 101.6 | | 100.3 | 97.8 | 95.9 |
| Methyl Parathion | 8 | 104.2 | N/A | N/A | | N/A | | N/A | | N/A | | N/A | | N/A | | N/A | | 95.7 | | 106.4 | | 98.0 | | 105.4 | | 109.4 | | 107.1 | | 105.8 | 105.8 | 105.4 |
| (Z)-Mevinphos | 12 | 98.6 | N/A | N/A | | N/A | | N/A | | 111.1 | | 103.8 | | 101.6 | | 96.8 | | 99.4 | | 97.8 | | 97.8 | | 96.6 | | 95.0 | | 95.1 | | 94.0 | 94.2 | 92.8 |
| (E)-Mevinphos | 16 | 102.7 | 97.2 | 100.1 | | 108.6 | | 106.1 | | 107.1 | | 106.3 | | 106.2 | | 104.5 | | 105.8 | | 104.2 | | 103.8 | | 103.6 | | 101.7 | | 97.7 | | 96.3 | 94.0 | 96.0 |
| Myclobutanil | 11 | 101.9 | N/A | N/A | | N/A | | N/A | | 117.3 | | 108.4 | | 103.8 | | 99.2 | | 99.8 | | 98.1 | | 98.6 | | 99.1 | | 98.7 | | 99.4 | | 98.5 | N/A | 100.3 |
| Naled | 10 | 88.1 | N/A | N/A | | N/A | | N/A | | N/A | | N/A | | 100.9 | | 91.1 | | 89.2 | | 85.6 | | 87.1 | | 84.5 | | 86.4 | | 85.0 | | 85.7 | 85.2 | 86.2 |
| Oxamyl | 14 | 107.1 | N/A | N/A | | 114.8 | | 112.2 | | 113.1 | | 108.9 | | 107.8 | | 106.3 | | 107.3 | | 106.6 | | 107.1 | | 107.0 | | 106.0 | | 102.3 | | 100.2 | 99.3 | 95.6 |
| Paclobutrazol | 11 | 103.3 | N/A | N/A | | N/A | | N/A | | N/A | | 117.6 | | 110.0 | | 104.0 | | 103.9 | | 101.6 | | 101.3 | | 101.9 | | 101.1 | | 99.4 | | 97.3 | 98.2 | 90.5 |
| (E)-Permethrin | 11 | 92.3 | N/A | N/A | | N/A | | N/A | | N/A | | 81.0 | | 86.6 | | 91.2 | | 93.5 | | 94.8 | | 93.5 | | 97.0 | | 95.6 | | 95.0 | | 93.4 | 93.3 | 89.5 |
| (Z)-Permethrin | 9 | 97 | N/A | N/A | | N/A | | N/A | | N/A | | N/A | | N/A | | 94.6 | | 96.2 | | 96.2 | | 98.1 | | 99.1 | | 97.3 | | 96.4 | | 96.7 | 98.7 | 99.8 |
| Phosmet | 10 | 114.9 | N/A | N/A | | N/A | | N/A | | N/A | | N/A | | 118.4 | | 116.0 | | 115.8 | | 114.8 | | 115.5 | | 115.5 | | 115.5 | | 114.6 | | 111.6 | 111.2 | 112.1 |
| Piperonyl Butoxide | 13 | 104.8 | N/A | N/A | | N/A | | 91.1 | | 99.5 | | 102.0 | | 105.4 | | 108.6 | | 109.2 | | 108.8 | | 108.2 | | 106.2 | | 107.1 | | 107.8 | | 106.1 | 102.7 | 109.8 |
| Prallethrin | 10 | 112.2 | N/A | N/A | | N/A | | N/A | | N/A | | N/A | | 116.3 | | 113.4 | | 114.2 | | 111.6 | | 110.8 | | 110.4 | | 111.2 | | 112.4 | | 110.7 | 111.1 | N/A |
| Propiconazole | 11 | 104.7 | N/A | N/A | | N/A | | N/A | | N/A | | 117.6 | | 109.7 | | 104.4 | | 104.3 | | 102.8 | | 103.0 | | 103.7 | | 102.7 | | 101.9 | | 100.7 | 100.9 | 98.4 |
| Propoxur | 12 | 117.4 | N/A | N/A | | N/A | | N/A | | 116.0 | | 117.9 | | 118.6 | | 116.9 | | 117.4 | | 118.5 | | 118.3 | | 118.1 | | 118.0 | | 117.5 | | 115.2 | 115.9 | 116.4 |
| Pyrethrin I | 10 | 92 | N/A | N/A | | N/A | | N/A | | N/A | | N/A | | 97.5 | | 101.8 | | 96.8 | | 92.4 | | 89.2 | | 84.0 | | 90.1 | | 97.2 | | 89.7 | 81.0 | N/A |
| Pyrethrin II | 9 | 113.5 | N/A | N/A | | N/A | | N/A | | N/A | | N/A | | N/A | | 119.2 | | 118.2 | | 119.7 | | 110.8 | | 106.6 | | 118.2 | | 119.6 | | 110.9 | 98.0 | N/A |
| Pyridaben | 7 | 89.2 | N/A | N/A | | N/A | | N/A | | N/A | | N/A | | N/A | | 103.5 | | 94.3 | | 87.8 | | 87.5 | | 87.8 | | 83.2 | | 80.5 | | N/A | N/A | 91.7 |
| Spinetoram J | 13 | 102.4 | N/A | N/A | | N/A | | 97.3 | | 99.6 | | 97.6 | | 98.9 | | 101.4 | | 108.1 | | 106.0 | | 107.1 | | 102.2 | | 107.6 | | 106.9 | | 101.0 | 97.5 | 106.7 |
| Spinetoram L | 11 | 98.1 | N/A | N/A | | N/A | | N/A | | N/A | | 93.3 | | 94.1 | | 97.1 | | 100.5 | | 99.3 | | 98.8 | | 96.5 | | 102.2 | | 102.9 | | 97.4 | 97.1 | 101.3 |
| Spinosyn A | 11 | 93.1 | N/A | N/A | | N/A | | N/A | | 86.2 | | 83.5 | | 87.1 | | 96.5 | | 98.4 | | 96.5 | | 94.1 | | 90.5 | | 98.8 | | 99.2 | | 93.6 | N/A | 92.8 |
| Spinosyn D | 9 | 106.4 | N/A | N/A | | N/A | | N/A | | N/A | | N/A | | N/A | | 86.4 | | 103.0 | | 110.6 | | 113.6 | | 108.9 | | 110.3 | | 105.5 | | 111.6 | 107.5 | N/A |
| Spiromesifen | 10 | 100.7 | N/A | N/A | | N/A | | N/A | | 102.4 | | 97.9 | | 101.4 | | 101.1 | | 104.0 | | 105.3 | | 101.1 | | 99.1 | | 97.9 | | 96.8 | | N/A | N/A | 92.7 |
| Spirotetramat | 15 | 101.4 | 103.1 | 112.4 | | 98.3 | | 108.1 | | 109.9 | | 104.5 | | 103.2 | | 100.1 | | 101.3 | | 100.5 | | 101.1 | | 100.4 | | 96.5 | | 93.3 | | 88.3 | N/A | 90.2 |
| Spiroxamine | 14 | 88.6 | N/A | N/A | | 81.0 | | 87.2 | | 88.7 | | 89.3 | | 89.1 | | 88.1 | | 89.6 | | 89.2 | | 90.1 | | 90.1 | | 90.2 | | 89.9 | | 88.9 | 89.5 | 88.9 |
| Tebuconazole | 11 | 103.5 | N/A | N/A | | N/A | | N/A | | N/A | | 117.4 | | 109.7 | | 102.5 | | 102.0 | | 99.8 | | 100.8 | | 102.4 | | 100.9 | | 100.5 | | 100.0 | 102.7 | 100.2 |
| Thiacloprid | 15 | 103.7 | N/A | 99.1 | | 102.3 | | 105.2 | | 110.4 | | 105.2 | | 107.3 | | 106.4 | | 107.7 | | 105.7 | | 106.2 | | 104.3 | | 103.1 | | 100.3 | | 96.4 | 95.5 | 94.9 |
| Thiamethoxam | 14 | 101.8 | N/A | N/A | | 113.6 | | 108.0 | | 104.5 | | 105.4 | | 102.6 | | 100.3 | | 101.0 | | 100.2 | | 99.9 | | 101.3 | | 99.0 | | 97.9 | | 96.8 | 95.2 | 87.5 |
| Trifloxystrobin | 11 | 109.9 | N/A | N/A | | N/A | | N/A | | N/A | | 116.5 | | 114.1 | | 117.1 | | 116.8 | | 114.3 | | 112.3 | | 109.6 | | 108.4 | | 105.2 | | 99.5 | 95.1 | 97.1 |
|  |  |  |  |  | |  | |  | |  | |  | |  | |  | |  | |  | |  | |  | |  | |  | |  |  |  |

Yellow highlight: 90-110%; Grey fill: Levels outside the AMR.

* Level 17 was evaluated at 1.0 µL injection volumes, which corresponded to a 10-fold dilution of on column material.

Supplementary Table 14. Inter-day Precision (% CV) (n=7 at each level assessed)

|  | **Compound** | **Levels Assessed** | **Average %CV across AMR** | | **Average %CV at Level Assessed (n = 7)** | | | | | | | | | | | | | | | | | | | | | | | | | | | | | | | | | |  |
| --- | --- | --- | --- | --- | --- | --- | --- | --- | --- | --- | --- | --- | --- | --- | --- | --- | --- | --- | --- | --- | --- | --- | --- | --- | --- | --- | --- | --- | --- | --- | --- | --- | --- | --- | --- | --- | --- | --- | --- |
|  |  |  |  |  | **1** | | **2** | | **3** | | **4** | | **5** | | **6** | | **7** | | **8** | | **9** | | **10** | | **11** | | **12** | | **13** | | **14** | | **15** | | **16** | | **17*** | |  |
| Acephate | | 14 | | 2.7 | | N/A | | N/A | | 4.5 | | 5.3 | | 4.9 | | 4.4 | | 3.4 | | 2.1 | | 1.7 | | 2.6 | | 1.3 | | 1.2 | | 1.3 | | 1.1 | | 1.6 | | 2.9 | | 3.6 | |
| Acequinocyl | | 11 | | 2.4 | | N/A | | N/A | | N/A | | N/A | | N/A | | 7.7 | | 2.8 | | 2.2 | | 2.9 | | 1.8 | | 1.4 | | 2.6 | | 1.4 | | 0.6 | | 1.6 | | 1.3 | | 6.6 | |
| Acetamiprid | | 14 | | 2.2 | | N/A | | N/A | | 5.4 | | 4.7 | | 3.1 | | 2.9 | | 2.7 | | 1.6 | | 1.4 | | 1.5 | | 1.2 | | 1.1 | | 1.5 | | 1.4 | | 2.0 | | 0.7 | | N/A | |
| Aldicarb | | 13 | | 3.6 | | N/A | | N/A | | N/A | | 8.2 | | 3.5 | | 5.2 | | 2.7 | | 3.1 | | 4.9 | | 1.8 | | 3.6 | | 1.4 | | 3.6 | | 1.8 | | 3.4 | | 3.2 | | 4.9 | |
| Avermectin B1a | | 7 | | 4.6 | | N/A | | N/A | | N/A | | N/A | | N/A | | N/A | | N/A | | N/A | | 6.3 | | 5.7 | | 4.4 | | 4.9 | | 3.7 | | 2.4 | | 4.7 | | N/A | | 10.9 | |
| Avermectin B1b | | 5 | | 4.5 | | N/A | | N/A | | N/A | | N/A | | N/A | | N/A | | N/A | | N/A | | N/A | | N/A | | N/A | | 6.2 | | 5.5 | | 2.7 | | 5.0 | | 2.9 | | N/A | |
| Azoxystrobin | | 15 | | 2.8 | | N/A | | 8.8 | | 5.9 | | 4.1 | | 4.9 | | 2.7 | | 1.6 | | 2.3 | | 2.3 | | 2.1 | | 1.2 | | 1.4 | | 1.5 | | 1.2 | | 1.3 | | 1.1 | | 3.4 | |
| Bifenazate | | 10 | | 3.4 | | N/A | | N/A | | N/A | | N/A | | N/A | | N/A | | 5.6 | | 3.7 | | 2.7 | | 3.5 | | 3.7 | | 2.2 | | 2.6 | | 2.1 | | 4.0 | | 3.5 | | 6.8 | |
| Bifenthrin | | 10 | | 3.8 | | N/A | | N/A | | N/A | | N/A | | N/A | | N/A | | 7.2 | | 4.9 | | 4.0 | | 1.9 | | 1.3 | | 5.3 | | 1.5 | | 1.3 | | 5.9 | | 4.2 | | N/A | |
| Boscalid | | 13 | | 2.1 | | N/A | | N/A | | N/A | | 5.6 | | 1.6 | | 2.6 | | 2.9 | | 1.8 | | 1.4 | | 2.1 | | 1.0 | | 1.4 | | 1.7 | | 1.9 | | 2.7 | | 1.2 | | 2.1 | |
| Captan | | 5 | | 6.8 | | N/A | | N/A | | N/A | | N/A | | N/A | | N/A | | N/A | | N/A | | N/A | | N/A | | N/A | | 7.1 | | 6.5 | | 5.7 | | 7.6 | | 7.1 | | 11.3 | |
| Carbaryl | | 14 | | 1.8 | | N/A | | N/A | | 2.5 | | 5.2 | | 1.8 | | 2.5 | | 1.1 | | 1.4 | | 1.7 | | 1.2 | | 0.5 | | 1.6 | | 1.2 | | 0.7 | | 1.6 | | 1.5 | | 4.0 | |
| Carbofuran | | 12 | | 1.8 | | N/A | | N/A | | N/A | | 4.2 | | 3.2 | | 1.2 | | 3.2 | | 0.3 | | 1.6 | | 1.6 | | 1.4 | | 1.4 | | 0.7 | | 0.9 | | 1.9 | | N/A | | 2.1 | |
| Chlorantraniliprole | | 12 | | 2.2 | | N/A | | N/A | | N/A | | N/A | | 2.3 | | 3.7 | | 3.6 | | 2.2 | | 2.1 | | 2.0 | | 1.9 | | 1.7 | | 1.1 | | 1.5 | | 2.2 | | 2.1 | | 3.2 | |
| Chlorfenapyr | | 7 | | 6.3 | | N/A | | N/A | | N/A | | N/A | | N/A | | N/A | | N/A | | N/A | | N/A | | 7.0 | | 6.7 | | 7.6 | | 7.3 | | 4.4 | | 5.8 | | 5.1 | | 11.2 | |
| Chlorpyrifos | | 13 | | 1.3 | | N/A | | N/A | | N/A | | 3.6 | | 1.1 | | 2.7 | | 1.8 | | 1.0 | | 1.0 | | 0.4 | | 1.0 | | 1.1 | | 0.7 | | 0.8 | | 1.4 | | 0.9 | | 3.7 | |
| Cinerin I | | 10 | | 2.9 | | N/A | | N/A | | N/A | | N/A | | N/A | | N/A | | 5.0 | | 4.2 | | 3.5 | | 2.2 | | 3.4 | | 3.8 | | 1.6 | | 2.0 | | 1.4 | | 1.6 | | N/A | |
| Cinerin II | | 9 | | 3.2 | | N/A | | N/A | | N/A | | N/A | | N/A | | N/A | | N/A | | 7.2 | | 6.5 | | 2.9 | | 3.2 | | 1.9 | | 2.0 | | 1.6 | | 1.6 | | 1.7 | | N/A | |
| Clofentezine | | 14 | | 1.9 | | N/A | | N/A | | 7.5 | | 3.0 | | 1.9 | | 2.1 | | 2.2 | | 1.5 | | 1.5 | | 0.7 | | 0.6 | | 1.3 | | 0.7 | | 1.4 | | 1.0 | | 1.8 | | 4.1 | |
| Coumaphos | | 13 | | 1.6 | | N/A | | N/A | | N/A | | 4.9 | | 1.8 | | 2.2 | | 1.7 | | 1.0 | | 1.1 | | 1.1 | | 1.4 | | 1.1 | | 0.8 | | 0.7 | | 2.1 | | 1.5 | | 3.3 | |
| Cyfluthrin | | 7 | | 3.1 | | N/A | | N/A | | N/A | | N/A | | N/A | | N/A | | N/A | | N/A | | N/A | | 4.7 | | 3.5 | | 3.9 | | 2.4 | | 1.9 | | 1.8 | | 3.7 | | 5.7 | |
| Cypermethrin | | 8 | | 1.6 | | N/A | | N/A | | N/A | | N/A | | N/A | | N/A | | N/A | | N/A | | 5.4 | | 1.1 | | 0.7 | | 1.0 | | 1.6 | | 1.1 | | 1.3 | | 0.7 | | 3.1 | |
| Daminozide | | 12 | | 2 | | N/A | | N/A | | N/A | | N/A | | 3.4 | | 1.3 | | 1.1 | | 0.8 | | 2.3 | | 4.2 | | 1.6 | | 2.1 | | 0.9 | | 2.8 | | 1.5 | | 2.0 | | 3.0 | |
| Diazinon | | 15 | | 2.1 | | N/A | | 4.6 | | 5.3 | | 4.7 | | 2.0 | | 2.0 | | 2.3 | | 1.0 | | 1.9 | | 1.0 | | 0.4 | | 1.7 | | 0.9 | | 1.1 | | 1.3 | | 1.4 | | 4.4 | |
| Dichlorvos | | 11 | | 1.7 | | N/A | | N/A | | N/A | | N/A | | N/A | | 4.1 | | 2.3 | | 2.4 | | 0.9 | | 0.7 | | 2.4 | | 3.0 | | 0.8 | | 1.0 | | 0.8 | | 0.6 | | 3.3 | |
| Dimethoate | | 14 | | 3.3 | | N/A | | N/A | | 9.6 | | 8.8 | | 3.2 | | 2.2 | | 2.8 | | 1.5 | | 1.9 | | 1.2 | | 2.1 | | 3.2 | | 1.2 | | 2.1 | | 4.4 | | 1.4 | | 3.4 | |
| (E)-Dimethomorph | | 11 | | 1.4 | | N/A | | N/A | | N/A | | N/A | | N/A | | 1.0 | | 2.9 | | 1.3 | | 1.9 | | 0.8 | | 1.5 | | 1.7 | | 0.9 | | 0.7 | | 2.0 | | 1.0 | | 4.0 | |
| (Z)-Dimethomorph | | 12 | | 1.5 | | N/A | | N/A | | N/A | | N/A | | 1.8 | | 1.9 | | 3.5 | | 1.1 | | 1.8 | | 1.2 | | 0.9 | | 1.6 | | 0.9 | | 1.0 | | 0.4 | | 1.6 | | 3.5 | |
| Ethoprophos | | 15 | | 1.9 | | N/A | | 4.9 | | 6.3 | | 1.5 | | 3.0 | | 2.1 | | 1.2 | | 1.4 | | 1.1 | | 0.7 | | 1.1 | | 2.1 | | 0.8 | | 0.8 | | 0.9 | | 1.1 | | 3.2 | |
| Etofenprox | | 12 | | 2.2 | | N/A | | N/A | | N/A | | N/A | | 8.0 | | 3.6 | | 2.4 | | 1.5 | | 1.4 | | 1.2 | | 1.4 | | 1.5 | | 1.4 | | 1.1 | | 1.4 | | 1.2 | | 5.0 | |
| Etoxazole | | 11 | | 1.3 | | N/A | | N/A | | N/A | | N/A | | 3.2 | | 1.4 | | 1.4 | | 1.2 | | 1.4 | | 0.9 | | 0.7 | | 0.8 | | 1.0 | | 0.9 | | N/A | | N/A | | 3.1 | |
| Fenhexamid | | 9 | | 2.1 | | N/A | | N/A | | N/A | | N/A | | N/A | | N/A | | 3.7 | | 2.2 | | 2.3 | | 1.7 | | 2.1 | | 1.2 | | 1.8 | | 1.5 | | N/A | | N/A | | N/A | |
| Fenoxycarb | | 13 | | 1.8 | | N/A | | N/A | | N/A | | 7.4 | | 2.0 | | 2.5 | | 2.4 | | 1.6 | | 1.5 | | 0.8 | | 0.8 | | 0.9 | | 0.8 | | 0.9 | | 1.3 | | 1.0 | | 3.3 | |
| Fenpyroximate | | 11 | | 1.6 | | N/A | | N/A | | N/A | | N/A | | 2.5 | | 1.2 | | 2.1 | | 1.7 | | 1.8 | | 1.0 | | 1.5 | | 1.4 | | 0.8 | | 2.3 | | 1.0 | | N/A | | 3.7 | |
| Fipronil | | 11 | | 2.8 | | N/A | | N/A | | N/A | | N/A | | 7.7 | | 5.0 | | 3.1 | | 2.1 | | 2.7 | | 1.6 | | 2.0 | | 1.6 | | 1.3 | | 1.1 | | 2.3 | | N/A | | N/A | |
| Flonicamid | | 14 | | 4.8 | | N/A | | N/A | | 8.4 | | 7.6 | | 7.2 | | 6.4 | | 6.4 | | 5.2 | | 3.3 | | 2.5 | | 3.6 | | 4.3 | | 4.2 | | 3.6 | | 2.2 | | 2.3 | | 5.9 | |
| Fludioxonil | | 11 | | 2.8 | | N/A | | N/A | | N/A | | N/A | | N/A | | 5.2 | | 3.6 | | 2.8 | | 2.9 | | 3.7 | | 2.4 | | 3.2 | | 2.1 | | 1.6 | | 1.4 | | 2.4 | | 6.1 | |
| Hexythiazox | | 12 | | 3 | | N/A | | N/A | | N/A | | 7.9 | | 6.5 | | 1.0 | | 5.2 | | 0.9 | | 3.3 | | 1.0 | | 2.1 | | 1.9 | | 1.6 | | 1.3 | | 2.7 | | N/A | | 5.2 | |
| Imazalil | | 16 | | 2.5 | | 8.9 | | 8.0 | | 5.6 | | 5.1 | | 1.3 | | 1.3 | | 2.2 | | 1.1 | | 1.1 | | 0.6 | | 0.6 | | 1.1 | | 0.6 | | 1.0 | | 1.1 | | 0.7 | | 3.8 | |
| Imidacloprid | | 16 | | 2.2 | | 9.9 | | 6.0 | | 2.0 | | 2.2 | | 2.3 | | 2.2 | | 0.8 | | 1.5 | | 1.3 | | 0.9 | | 0.7 | | 0.5 | | 1.1 | | 0.9 | | 1.5 | | 2.0 | | 3.0 | |
| Jasmolin I | | 7 | | 4.3 | | N/A | | N/A | | N/A | | N/A | | N/A | | N/A | | N/A | | N/A | | 6.7 | | 5.7 | | 5.0 | | 2.8 | | 4.0 | | 3.6 | | 2.3 | | N/A | | N/A | |
| Jasmolin II | | 7 | | 3.1 | | N/A | | N/A | | N/A | | N/A | | N/A | | N/A | | N/A | | N/A | | N/A | | 1.2 | | 3.9 | | 4.2 | | 3.3 | | 2.2 | | 3.7 | | 2.9 | | N/A | |
| Kresoxim-methyl | | 12 | | 4.4 | | N/A | | N/A | | N/A | | N/A | | 6.9 | | 6.4 | | 7.0 | | 4.8 | | 2.7 | | 3.8 | | 3.9 | | 4.9 | | 3.6 | | 2.4 | | 3.9 | | 2.6 | | 4.4 | |
| Malathion | | 15 | | 2.5 | | N/A | | 8.7 | | 7.1 | | 2.4 | | 3.1 | | 2.3 | | 1.9 | | 1.7 | | 1.6 | | 1.7 | | 1.0 | | 0.8 | | 1.3 | | 1.0 | | 1.8 | | 1.2 | | 4.4 | |
| Metalaxyl | | 13 | | 1.7 | | N/A | | N/A | | N/A | | 3.8 | | 2.4 | | 1.2 | | 2.1 | | 1.3 | | 1.6 | | 1.4 | | 1.2 | | 1.8 | | 2.1 | | 1.2 | | 1.8 | | 0.5 | | 2.9 | |
| Methiocarb | | 11 | | 1.9 | | N/A | | N/A | | N/A | | N/A | | 4.1 | | 4.7 | | 3.0 | | 1.6 | | 1.1 | | 1.2 | | 1.0 | | 0.8 | | 0.6 | | 1.7 | | 1.0 | | N/A | | 4.2 | |
| Methomyl | | 13 | | 3 | | N/A | | N/A | | N/A | | 8.7 | | 6.7 | | 3.6 | | 3.9 | | 2.2 | | 2.5 | | 0.8 | | 1.6 | | 1.8 | | 1.6 | | 1.8 | | 1.8 | | 2.5 | | 2.6 | |
| Methyl Parathion | | 8 | | 5.3 | | N/A | | N/A | | N/A | | N/A | | N/A | | N/A | | N/A | | N/A | | 10.6 | | 5.5 | | 6.7 | | 7.4 | | 3.8 | | 4.2 | | 2.1 | | 2.0 | | 6.5 | |
| (Z)-Mevinphos | | 12 | | 2.7 | | N/A | | N/A | | N/A | | N/A | | 8.5 | | 2.9 | | 3.0 | | 2.0 | | 2.3 | | 1.3 | | 2.5 | | 2.5 | | 1.3 | | 2.6 | | 1.6 | | 2.2 | | 2.8 | |
| (E)-Mevinphos | | 16 | | 3.2 | | 9.7 | | 8.0 | | 4.9 | | 5.3 | | 2.5 | | 2.0 | | 2.1 | | 1.7 | | 2.1 | | 1.3 | | 1.6 | | 3.4 | | 1.3 | | 1.8 | | 2.1 | | 1.3 | | 3.6 | |
| Myclobutanil | | 11 | | 1.8 | | N/A | | N/A | | N/A | | N/A | | 2.5 | | 3.4 | | 2.1 | | 1.5 | | 1.6 | | 2.8 | | 1.1 | | 1.2 | | 1.2 | | 1.1 | | 1.8 | | N/A | | 4.6 | |
| Naled | | 10 | | 1.6 | | N/A | | N/A | | N/A | | N/A | | N/A | | N/A | | 2.2 | | 1.6 | | 1.8 | | 1.2 | | 1.4 | | 2.9 | | 0.9 | | 1.8 | | 0.7 | | 1.4 | | 3.2 | |
| Oxamyl | | 14 | | 1.7 | | N/A | | N/A | | 2.4 | | 2.3 | | 2.0 | | 1.9 | | 1.8 | | 2.4 | | 1.1 | | 1.8 | | 1.0 | | 1.0 | | 1.9 | | 1.3 | | 1.7 | | 1.1 | | 3.7 | |
| Paclobutrazol | | 11 | | 2 | | N/A | | N/A | | N/A | | N/A | | N/A | | 5.0 | | 2.3 | | 2.6 | | 1.6 | | 2.0 | | 1.5 | | 1.6 | | 0.8 | | 1.2 | | 1.4 | | 1.6 | | 2.1 | |
| (E)-Permethrin | | 11 | | 2.2 | | N/A | | N/A | | N/A | | N/A | | N/A | | 6.2 | | 2.5 | | 2.2 | | 1.8 | | 1.5 | | 2.2 | | 1.4 | | 1.4 | | 1.6 | | 1.7 | | 2.0 | | 4.9 | |
| (Z)-Permethrin | | 9 | | 1.5 | | N/A | | N/A | | N/A | | N/A | | N/A | | N/A | | N/A | | 2.2 | | 1.8 | | 1.1 | | 0.8 | | 1.4 | | 0.9 | | 1.2 | | 1.9 | | 1.9 | | 5.9 | |
| Phosmet | | 10 | | 1.5 | | N/A | | N/A | | N/A | | N/A | | N/A | | N/A | | 1.6 | | 2.4 | | 2.6 | | 1.4 | | 0.9 | | 0.9 | | 1.1 | | 1.5 | | 1.3 | | 1.5 | | 4.2 | |
| Piperonyl Butoxide | | 13 | | 1.7 | | N/A | | N/A | | N/A | | 3.0 | | 2.5 | | 2.8 | | 2.3 | | 1.3 | | 1.5 | | 0.6 | | 1.4 | | 1.5 | | 1.5 | | 1.5 | | 1.7 | | 1.1 | | 4.5 | |
| Prallethrin | | 10 | | 1.8 | | N/A | | N/A | | N/A | | N/A | | N/A | | N/A | | 2.1 | | 1.8 | | 2.2 | | 1.1 | | 2.1 | | 1.9 | | 1.6 | | 1.7 | | 1.5 | | 1.6 | | N/A | |
| Propiconazole | | 11 | | 1.1 | | N/A | | N/A | | N/A | | N/A | | N/A | | 1.5 | | 1.9 | | 0.8 | | 1.1 | | 0.8 | | 0.7 | | 0.6 | | 0.8 | | 1.2 | | 1.3 | | 1.1 | | 4.0 | |
| Propoxur | | 12 | | 1.3 | | N/A | | N/A | | N/A | | N/A | | 1.6 | | 0.7 | | 1.8 | | 1.7 | | 1.5 | | 0.3 | | 1.1 | | 1.5 | | 1.3 | | 1.2 | | 1.8 | | 1.5 | | 1.9 | |
| Pyrethrin I | | 10 | | 4 | | N/A | | N/A | | N/A | | N/A | | N/A | | N/A | | 8.5 | | 4.7 | | 4.1 | | 3.5 | | 2.4 | | 3.9 | | 3.7 | | 1.6 | | 3.2 | | 4.0 | | N/A | |
| Pyrethrin II | | 9 | | 3.3 | | N/A | | N/A | | N/A | | N/A | | N/A | | N/A | | N/A | | 3.2 | | 2.7 | | 3.1 | | 4.7 | | 3.3 | | 4.6 | | 1.5 | | 2.9 | | 3.3 | | N/A | |
| Pyridaben | | 7 | | 1.5 | | N/A | | N/A | | N/A | | N/A | | N/A | | N/A | | N/A | | 2.2 | | 1.6 | | 1.0 | | 1.5 | | 1.2 | | 0.7 | | 2.5 | | N/A | | N/A | | 3.6 | |
| Spinetoram J | | 13 | | 3.8 | | N/A | | N/A | | N/A | | 6.4 | | 3.7 | | 4.9 | | 6.4 | | 4.4 | | 2.4 | | 2.1 | | 3.0 | | 2.9 | | 2.2 | | 6.6 | | 1.9 | | 3.0 | | 3.2 | |
| Spinetoram L | | 11 | | 3.3 | | N/A | | N/A | | N/A | | N/A | | N/A | | 5.1 | | 5.5 | | 3.5 | | 2.0 | | 2.6 | | 2.0 | | 3.5 | | 1.6 | | 5.9 | | 2.4 | | 2.1 | | 5.2 | |
| Spinosyn A | | 11 | | 2.9 | | N/A | | N/A | | N/A | | N/A | | 1.9 | | 2.4 | | 6.9 | | 2.0 | | 1.8 | | 2.1 | | 2.3 | | 3.5 | | 1.6 | | 5.4 | | 2.0 | | N/A | | 3.8 | |
| Spinosyn D | | 9 | | 3.7 | | N/A | | N/A | | N/A | | N/A | | N/A | | N/A | | N/A | | 3.5 | | 4.2 | | 1.8 | | 4.5 | | 4.4 | | 3.1 | | 7.7 | | 2.7 | | 1.6 | | N/A | |
| Spiromesifen | | 10 | | 5.8 | | N/A | | N/A | | N/A | | N/A | | 9.1 | | 7.8 | | 6.2 | | 4.4 | | 6.0 | | 5.9 | | 3.9 | | 5.1 | | 4.1 | | 5.4 | | N/A | | N/A | | 7.0 | |
| Spirotetramat | | 15 | | 3.4 | | 8.9 | | 8.6 | | 7.8 | | 6.5 | | 3.3 | | 1.7 | | 1.9 | | 1.3 | | 2.2 | | 1.1 | | 1.5 | | 1.7 | | 1.5 | | 1.1 | | 1.5 | | N/A | | 4.4 | |
| Spiroxamine | | 14 | | 1.9 | | N/A | | N/A | | 7.4 | | 1.8 | | 2.8 | | 1.7 | | 2.4 | | 0.9 | | 1.8 | | 1.3 | | 1.1 | | 1.8 | | 1.0 | | 0.9 | | 0.9 | | 1.2 | | 3.6 | |
| Tebuconazole | | 11 | | 1.4 | | N/A | | N/A | | N/A | | N/A | | N/A | | 1.8 | | 2.6 | | 1.6 | | 1.4 | | 0.8 | | 0.8 | | 0.7 | | 1.1 | | 1.2 | | 1.4 | | 1.9 | | 3.3 | |
| Thiacloprid | | 15 | | 2.5 | | N/A | | 7.6 | | 6.1 | | 3.0 | | 1.7 | | 2.6 | | 2.1 | | 2.5 | | 2.1 | | 1.2 | | 1.8 | | 0.9 | | 1.6 | | 0.7 | | 1.2 | | 1.8 | | 3.0 | |
| Thiamethoxam | | 14 | | 2.4 | | N/A | | N/A | | 5.7 | | 2.3 | | 3.7 | | 2.7 | | 2.7 | | 2.1 | | 2.5 | | 1.8 | | 0.8 | | 2.1 | | 1.9 | | 2.0 | | 1.6 | | 1.6 | | 2.9 | |
| Trifloxystrobin | | 11 | | 1.8 | | N/A | | N/A | | N/A | | N/A | | N/A | | 2.8 | | 3.3 | | 2.6 | | 1.3 | | 1.1 | | 1.7 | | 2.0 | | 1.7 | | 1.2 | | 1.0 | | 0.9 | | 3.3 | |

Yellow highlight: 5-10%; Orange highlight: 10-15%; Grey fill: Levels outside the AMR.

* Level 17 was evaluated at 1.0 µL injection volumes, which corresponded to a 10-fold dilution of on column material.

Supplementary Table 15. Average Inter-day Accuracy (% Recovery) and Precision (%CV) (n=7 at each level assessed)

| **Compound** | **Levels Assessed**  **Within primary AMR** | **Across primary AMR** | | **Across 120 spikes at**  **100 PPB in-sample** | |
| --- | --- | --- | --- | --- | --- |
|  |  | **Average**  **% Recovery** | **Average**  **%CV** | **Average**  **% Recovery** | **Average**  **%CV** |
| Acephate | 14 | 104 | 2.7 | 101.5 | 2.5 |
| Acequinocyl | 11 | 91.3 | 2.4 | 89.8 | 3.6 |
| Acetamiprid | 14 | 103.6 | 2.2 | 106.4 | 2.0 |
| Aldicarb | 13 | 99.4 | 3.6 | 99.3 | 2.9 |
| Avermectin B1a | 7 | 111.6 | 4.6 | 111.2 | 12.5 |
| Avermectin B1b | 5 | 104.7 | 4.5 | 98.6 | 31.8 |
| Azoxystrobin | 15 | 105.2 | 2.8 | 106.5 | 2.5 |
| Bifenazate | 10 | 116.8 | 3.4 | 105.4 | 7.7 |
| Bifenthrin | 10 | 101.7 | 3.8 | 92.2 | 5.2 |
| Boscalid | 13 | 112.5 | 2.1 | 104.9 | 3.9 |
| Captan | 5 | 110.6 | 6.8 | N/A | N/A |
| Carbaryl | 14 | 100 | 1.8 | 99.4 | 1.8 |
| Carbofuran | 12 | 104.6 | 1.8 | 97.0 | 1.9 |
| Chlorantraniliprole | 12 | 102.2 | 2.2 | 97.5 | 2.8 |
| Chlorfenapyr | 7 | 102.9 | 6.3 | 93.6 | 31.0 |
| Chlorpyrifos | 13 | 92.2 | 1.3 | 93.1 | 1.8 |
| Cinerin I | 10 | 91.5 | 2.9 | 88.1 | 3.8 |
| Cinerin II | 9 | 98.7 | 3.2 | 101.3 | 4.8 |
| Clofentezine | 14 | 99.9 | 1.9 | 97.5 | 1.8 |
| Coumaphos | 13 | 90 | 1.6 | 92.9 | 2.0 |
| Cyfluthrin | 7 | 109.5 | 3.1 | 96.7 | 8.5 |
| Cypermethrin | 8 | 109.6 | 1.6 | 95.4 | 4.1 |
| Daminozide | 12 | 89.7 | 2 | 88.1 | 8.2 |
| Diazinon | 15 | 96.9 | 2.1 | 98.2 | 2.7 |
| Dichlorvos | 11 | 103.2 | 1.7 | 104.7 | 2.8 |
| Dimethoate | 14 | 100.6 | 3.3 | 104.4 | 3.6 |
| (E)-Dimethomorph | 11 | 104.5 | 1.4 | 102.6 | 2.6 |
| (Z)-Dimethomorph | 12 | 106.7 | 1.5 | 110.0 | 2.9 |
| Ethoprophos | 15 | 103.2 | 1.9 | 99.9 | 1.8 |
| Etofenprox | 12 | 97.7 | 2.2 | 95.0 | 2.5 |
| Etoxazole | 11 | 90.4 | 1.3 | 94.1 | 1.7 |
| Fenhexamid | 9 | 102 | 2.1 | 108.0 | 3.1 |
| Fenoxycarb | 13 | 98.3 | 1.8 | 95.4 | 1.8 |
| Fenpyroximate | 11 | 111.2 | 1.6 | 92.9 | 2.4 |
| Fipronil | 11 | 103.3 | 2.8 | 102.9 | 3.0 |
| Flonicamid | 14 | 97.8 | 4.8 | 96.5 | 4.1 |
| Fludioxonil | 11 | 101.1 | 2.8 | 98.7 | 3.4 |
| Hexythiazox | 12 | 92.9 | 3 | 103.0 | 3.1 |
| Imazalil | 16 | 98.8 | 2.5 | 96.9 | 1.7 |
| Imidacloprid | 16 | 104.7 | 2.2 | 104.7 | 1.6 |
| Jasmolin I | 7 | 100.5 | 4.3 | 108.4 | 9.2 |
| Jasmolin II | 7 | 103.1 | 3.1 | 89.8 | 6.2 |
| Kresoxim Methyl | 12 | 105.4 | 4.4 | 97.8 | 3.7 |
| Malathion | 15 | 102.9 | 2.5 | 103.4 | 2.1 |
| Metalaxyl | 13 | 108.2 | 1.7 | 107.4 | 2.0 |
| Methiocarb | 11 | 100 | 1.9 | 99.7 | 2.1 |
| Methomyl | 13 | 102.3 | 3 | 100.7 | 2.9 |
| Methyl Parathion | 8 | 104.2 | 5.3 | 102.8 | 11.0 |
| (Z)-Mevinphos | 12 | 98.6 | 2.7 | 100.4 | 2.2 |
| (E)-Mevinphos | 16 | 102.7 | 3.2 | 104.8 | 1.9 |
| Myclobutanil | 11 | 101.9 | 1.8 | 99.4 | 2.5 |
| Naled | 10 | 88.1 | 1.6 | 86.8 | 4.5 |
| Oxamyl | 14 | 107.1 | 1.7 | 104.4 | 2.0 |
| Paclobutrazol | 11 | 103.3 | 2 | 99.5 | 2.2 |
| (E)-Permethrin | 11 | 92.3 | 2.2 | 96.8 | 3.7 |
| (Z)-Permethrin | 9 | 97 | 1.5 | 93.4 | 3.5 |
| Phosmet | 10 | 114.9 | 1.5 | 103.7 | 2.6 |
| Piperonyl Butoxide | 13 | 104.8 | 1.7 | 103.9 | 3.1 |
| Prallethrin | 10 | 112.2 | 1.8 | 99.8 | 2.2 |
| Propiconazole | 11 | 104.7 | 1.1 | 102.2 | 2.1 |
| Propoxur | 12 | 117.4 | 1.3 | 99.6 | 1.9 |
| Pyrethrin I | 10 | 92 | 4 | 88.2 | 4.5 |
| Pyrethrin II | 9 | 113.5 | 3.3 | 102.1 | 6.0 |
| Pyridaben | 7 | 89.2 | 1.5 | 88.8 | 3.6 |
| Spinetoram J | 13 | 102.4 | 3.8 | 86.4 | 5.9 |
| Spinetoram L | 11 | 98.1 | 3.3 | 81.1 | 5.5 |
| Spinosyn A | 11 | 93.1 | 2.9 | 85.8 | 5.1 |
| Spinosyn D | 9 | 106.4 | 3.7 | 105.2 | 6.5 |
| Spiromesifen | 10 | 100.7 | 5.8 | 101.1 | 5.2 |
| Spirotetramat | 15 | 101.4 | 3.4 | 105.5 | 2.1 |
| Spiroxamine | 14 | 88.6 | 1.9 | 92.4 | 1.9 |
| Tebuconazole | 11 | 103.5 | 1.4 | 99.7 | 2.0 |
| Thiacloprid | 15 | 103.7 | 2.5 | 104.1 | 2.2 |
| Thiamethoxam | 14 | 101.8 | 2.4 | 102.0 | 2.6 |
| Trifloxystrobin | 11 | 109.9 | 1.8 | 99.6 | 2.7 |

Supplementary Table 16. Carryover Assessment at LLOQ

| **Analyte** | **Carryover at LLOQ (%)** | **LLOQ Level** |
| --- | --- | --- |
| Imazalil | 3.9 | 1 |
| Imidacloprid | 0.8 | 1 |
| (E)-Mevinphos | 2.2 | 1 |
| Spirotetramat | 8.7 | 1 |
| Azoxystrobin | -9.7 | 2 |
| Diazinon | 2.8 | 2 |
| Ethoprophos | 9.3 | 2 |
| Malathion | -7.2 | 2 |
| Thiacloprid | 9.1 | 2 |
| Acephate | -3.2 | 3 |
| Acetamiprid | 8.3 | 3 |
| Carbaryl | -1.4 | 3 |
| Clofentezine | -5.2 | 3 |
| Dimethoate | 1.0 | 3 |
| Flonicamid | -8.9 | 3 |
| Oxamyl | -1.8 | 3 |
| Spiroxamine | 4.0 | 3 |
| Thiamethoxam | 4.6 | 3 |
| Aldicarb | -8.6 | 4 |
| Boscalid | 0.4 | 4 |
| Carbofuran | 7.1 | 4 |
| Chlorpyrifos | -0.1 | 4 |
| Coumaphos | -2.7 | 4 |
| Fenoxycarb | 7.5 | 4 |
| Hexythiazox | 8.1 | 4 |
| Metalaxyl | -6.7 | 4 |
| Methomyl | 2.5 | 4 |
| Piperonyl Butoxide | 2.6 | 4 |
| Spinetoram J | 2.0 | 4 |
| Chlorantraniliprole | 4.7 | 5 |
| Daminozide | 1.2 | 5 |
| (Z)-Dimethomorph | -5.9 | 5 |
| Etofenprox | -1.6 | 5 |
| Etoxazole | 7.6 | 5 |
| Fenpyroximate | -0.2 | 5 |
| Fipronil | 2.7 | 5 |
| Kresoxim Methyl | -4.5 | 5 |
| Methiocarb | -1.7 | 5 |
| (Z)-Mevinphos | 4.2 | 5 |
| Myclobutanil | 2.7 | 5 |
| Propoxur | 9.9 | 5 |
| Spinosyn A | -1.1 | 5 |
| Spiromesifen | -0.6 | 5 |
| Acequinocyl | -3.3 | 6 |
| Dichlorvos | -4.2 | 6 |
| (E)-Dimethomorph | 5.0 | 6 |
| Fludioxonil | 8.9 | 6 |
| Paclobutrazol | -1.3 | 6 |
| (E)-Permethrin | 1.8 | 6 |
| Propiconazole | -4.5 | 6 |
| Spinetoram L | -8.1 | 6 |
| Tebuconazole | 0.8 | 6 |
| Trifloxystrobin | 3.3 | 6 |
| Bifenazate | 3.8 | 7 |
| Bifenthrin | 2.6 | 7 |
| Cinerin I | -4.7 | 7 |
| Fenhexamid | 2.9 | 7 |
| Naled | 3.6 | 7 |
| Phosmet | -3.6 | 7 |
| Prallethrin | -0.2 | 7 |
| Pyrethrin I | 4.8 | 7 |
| Cinerin II | -0.2 | 8 |
| (Z)-Permethrin | -7.2 | 8 |
| Pyrethrin II | 8.3 | 8 |
| Pyridaben | -4.4 | 8 |
| Spinosyn D | 6.4 | 8 |
| Avermectin B1a | 9.8 | 9 |
| Cypermethrin | 3.9 | 9 |
| Jasmolin I | -5.9 | 9 |
| Methyl Parathion | -9.2 | 9 |
| Chlorfenapyr | -8.2 | 10 |
| Cyfluthrin | 3.7 | 10 |
| Jasmolin II | -7.3 | 10 |
| Avermectin B1b | 0.2 | 12 |
| Captan | -8.2 | 12 |

Supplementary Table 17. Ion Ratio (IR) Tolerances

| **Analyte** | **N** | **Levels Assessed** | **Average IR Across AMR** | **Average IR Precision Across AMR (%CV)** | **%CV *3** | **IR Tolerance +/-** |
| --- | --- | --- | --- | --- | --- | --- |
| Acephate | 84 | 14 | 13.3010 | 2.5 | 7.6 | 20% |
| Acequinocyl | 63 | 11 | 0.2089 | 1.3 | 4.0 | 20% |
| Acetamiprid | 84 | 14 | 1.0965 | 0.9 | 2.6 | 20% |
| Aldicarb | 77 | 13 | 2.1627 | 3.8 | 11.4 | 20% |
| Avermectin B1a | 42 | 7 | 0.1381 | 9.1 | 27.4 | 30% |
| Avermectin B1b | 21 | 5 | 0.0729 | 13.6 | 40.7 | 30% |
| Azoxystrobin | 91 | 15 | 3.0200 | 10.2 | 30.5 | 30% |
| Bifenazate | 56 | 10 | 0.6464 | 3.2 | 9.7 | 20% |
| Bifenthrin | 56 | 10 | 2.2247 | 11.8 | 35.3 | 30% |
| Boscalid | 77 | 13 | 0.1432 | 1.7 | 5.2 | 20% |
| Captan | 21 | 5 | 0.3513 | 4.2 | 12.5 | 20% |
| Carbaryl | 84 | 14 | 0.4408 | 1.5 | 4.5 | 20% |
| Carbofuran | 77 | 12 | 0.6481 | 1.1 | 3.4 | 20% |
| Chlorantraniliprole | 70 | 12 | 0.7310 | 2.9 | 8.7 | 20% |
| Chlorfenapyr | 35 | 7 | 2.0215 | 9.1 | 27.3 | 30% |
| Chlorpyrifos | 77 | 13 | 1.3307 | 0.7 | 2.2 | 20% |
| Cinerin I | 56 | 10 | 0.7514 | 1.9 | 5.8 | 20% |
| Cinerin II | 49 | 9 | 0.7189 | 2.0 | 5.9 | 20% |
| Clofentezine | 84 | 14 | 2.1237 | 0.7 | 2.2 | 20% |
| Coumaphos | 77 | 13 | 0.3011 | 3.0 | 9.1 | 20% |
| Cyfluthrin | 35 | 7 | 0.1666 | 3.6 | 10.9 | 20% |
| Cypermethrin | 42 | 8 | 0.0953 | 11.3 | 34.0 | 30% |
| Daminozide | 70 | 12 | 0.1786 | 4.8 | 14.4 | 20% |
| Diazinon | 91 | 15 | 0.8650 | 1.0 | 3.0 | 20% |
| Dichlorvos | 63 | 11 | 1.4735 | 1.0 | 3.1 | 20% |
| Dimethoate | 84 | 14 | 0.5480 | 5.9 | 17.7 | 20% |
| (E)-Dimethomorph | 63 | 11 | 0.3446 | 1.4 | 4.1 | 20% |
| (Z)-Dimethomorph | 70 | 12 | 0.3300 | 1.2 | 3.5 | 20% |
| Ethoprophos | 91 | 15 | 0.8714 | 5.8 | 17.4 | 20% |
| Etofenprox | 70 | 12 | 0.3404 | 1.4 | 4.3 | 20% |
| Etoxazole | 70 | 10 | 3.7063 | 2.5 | 7.5 | 20% |
| Fenhexamid | 56 | 8 | 0.0710 | 1.2 | 3.6 | 20% |
| Fenoxycarb | 77 | 13 | 1.0500 | 0.5 | 1.5 | 20% |
| Fenpyroximate | 70 | 11 | 7.0980 | 3.0 | 8.9 | 20% |
| Fipronil | 70 | 11 | 0.2317 | 2.5 | 7.5 | 20% |
| Flonicamid | 84 | 14 | 0.7751 | 4.3 | 13.0 | 20% |
| Fludioxonil | 63 | 11 | 0.5192 | 2.9 | 8.6 | 20% |
| Hexythiazox | 77 | 12 | 0.6202 | 0.8 | 2.3 | 20% |
| Imazalil | 98 | 16 | 1.7978 | 4.1 | 12.2 | 20% |
| Imidacloprid | 98 | 16 | 0.8503 | 3.0 | 9.0 | 20% |
| Jasmolin I | 42 | 7 | 0.9380 | 14.0 | 42.0 | 30% |
| Jasmolin II | 35 | 7 | 0.3841 | 2.8 | 8.3 | 20% |
| Kresoxim Methyl | 70 | 12 | 0.8613 | 3.8 | 11.5 | 20% |
| Malathion | 91 | 15 | 0.9932 | 2.7 | 8.0 | 20% |
| Metalaxyl | 77 | 13 | 0.7342 | 0.9 | 2.8 | 20% |
| Methiocarb | 70 | 11 | 0.1423 | 2.3 | 7.0 | 20% |
| Methomyl | 77 | 13 | 0.7840 | 1.5 | 4.6 | 20% |
| Methyl Parathion | 42 | 8 | 1.5437 | 3.6 | 10.8 | 20% |
| (Z)-Mevinphos | 70 | 12 | 1.2196 | 1.7 | 5.2 | 20% |
| (E)-Mevinphos | 98 | 16 | 1.0136 | 2.6 | 7.8 | 20% |
| Myclobutanil | 70 | 11 | 0.4924 | 3.4 | 10.2 | 20% |
| Naled | 56 | 10 | 0.8941 | 0.8 | 2.5 | 20% |
| Oxamyl | 84 | 14 | 0.4590 | 1.4 | 4.1 | 20% |
| Paclobutrazol | 63 | 11 | 0.2134 | 0.8 | 2.3 | 20% |
| (E)-Permethrin | 63 | 11 | 0.4751 | 2.7 | 8.1 | 20% |
| (Z)-Permethrin | 49 | 9 | 0.4807 | 0.8 | 2.3 | 20% |
| Phosmet | 56 | 10 | 0.1973 | 0.9 | 2.7 | 20% |
| Piperonyl Butoxide | 77 | 13 | 4.3207 | 3.5 | 10.4 | 20% |
| Prallethrin | 56 | 10 | 1.1361 | 1.5 | 4.5 | 20% |
| Propiconazole | 63 | 11 | 0.1927 | 0.6 | 1.8 | 20% |
| Propoxur | 70 | 12 | 0.8258 | 1.7 | 5.1 | 20% |
| Pyrethrin I | 56 | 10 | 0.2814 | 2.8 | 8.4 | 20% |
| Pyrethrin II | 49 | 9 | 0.2311 | 4.9 | 14.7 | 20% |
| Pyridaben | 49 | 7 | 1.3301 | 0.4 | 1.1 | 20% |
| Spinetoram J | 77 | 13 | 0.2931 | 3.2 | 9.5 | 20% |
| Spinetoram L | 63 | 11 | 0.2979 | 2.8 | 8.3 | 20% |
| Spinosyn A | 70 | 11 | 0.2306 | 0.8 | 2.4 | 20% |
| Spinosyn D | 49 | 9 | 0.0546 | 1.5 | 4.5 | 20% |
| Spiromesifen | 70 | 10 | 0.7979 | 4.1 | 12.3 | 20% |
| Spirotetramat | 98 | 15 | 0.8529 | 1.2 | 3.6 | 20% |
| Spiroxamine | 84 | 14 | 3.0370 | 2.0 | 6.0 | 20% |
| Tebuconazole | 63 | 11 | 0.1588 | 1.0 | 2.9 | 20% |
| Thiacloprid | 91 | 15 | 9.4588 | 2.0 | 6.1 | 20% |
| Thiamethoxam | 84 | 14 | 0.7034 | 2.2 | 6.7 | 20% |
| Trifloxystrobin | 63 | 11 | 6.0397 | 11.3 | 33.9 | 30% |

Supplementary Table 18. Matrix Effects

| **Compound** | **% Matrix effects** | | | |
| --- | --- | --- | --- | --- |
|  | **Calibrator concentration (ng/mL)** | | | |
|  | 12.5 (n = 2) | 25 (n = 4) | 50 (n = 12) | 100 (n = 4) |
| Acephate | 100.2 | 95.2 | 98.8 | 97.9 |
| Acequinocyl |  |  | 155.7* |  |
| Acetamiprid | 98.0 | 95.0 | 98.0 | 98.4 |
| Aldicarb | 95.0 | 98.9 | 97.1 | 95.7 |
| Avermectin B1a | 84.1 | 95.9 | 91.0 | 94.2 |
| Avermectin B1b | 84.7 | 84.6 | 85.5 | 86.3 |
| Azoxystrobin | 101.1 | 96.6 | 100.3 | 100.2 |
| Bifenazate |  |  | 86.3 |  |
| Bifenthrin | 209.9 | 206.9 | 190.7 | 186.6 |
| Boscalid | 95.9 | 99.6 | 98.3 | 101.2 |
| Captan |  |  | 134.8* |  |
| Carbaryl | 97.5 | 95.8 | 97.8 | 98.6 |
| Carbofuran | 96.6 | 97.0 | 96.5 | 98.7 |
| Chlorantraniliprole | 99.3 | 96.7 | 99.2 | 98.2 |
| Chlorfenapyr | 111.4 | 112.9 | 112.4 | 111.1 |
| Chlorpyrifos | 103.1 | 98.2 | 107.8 | 106.4 |
| Cinerin I | 108.7 | 105.2 | 113.6 | 113.6 |
| Cinerin II | 97.5 | 99.5 | 106.3 | 107.3 |
| Clofentezine | 104.6 | 97.2 | 105.5 | 103.1 |
| Coumaphos | 102.3 | 103.8 | 109.8 | 109.2 |
| Cyfluthrin | 135.4 | 163.5 | 164.1 | 178.7 |
| Cypermethrin | 147.1 | 157.9 | 173.5 | 179.1 |
| Daminozide | 95.9 | 92.9 | 97.0 | 97.1 |
| Diazinon | 98.8 | 96.0 | 100.1 | 100.6 |
| Dichlorvos | 97.7 | 94.6 | 96.1 | 96.2 |
| Dimethoate | 97.1 | 96.5 | 97.3 | 98.0 |
| (E)-Dimethomorph | 98.1 | 96.0 | 100.4 | 100.4 |
| (Z)-Dimethomorph | 95.4 | 95.9 | 98.6 | 99.0 |
| Ethoprophos | 98.0 | 95.1 | 99.0 | 99.4 |
| Etofenprox | 113.0 | 121.9 | 140.7 | 138.7 |
| Etoxazole | 99.1 | 101.2 | 105.7 | 105.4 |
| Fenhexamid | 98.1 | 94.8 | 98.8 | 100.5 |
| Fenoxycarb | 99.5 | 97.1 | 102.4 | 102.8 |
| Fenpyroximate | 101.6 | 105.0 | 114.7 | 113.5 |
| Fipronil | 98.6 | 94.3 | 98.9 | 98.9 |
| Flonicamid | 97.9 | 95.3 | 97.3 | 97.2 |
| Fludioxonil | 99.4 | 95.9 | 99.0 | 97.3 |
| Hexythiazox | 93.6 | 94.9 | 100.4 | 100.4 |
| Imazalil | 95.1 | 91.7 | 97.1 | 94.9 |
| Imidacloprid | 98.5 | 96.5 | 99.3 | 98.4 |
| Jasmolin I | 117.9 | 123.9 | 130.3 | 127.7 |
| Jasmolin II | 105.9 | 111.8 | 110.6 | 110.6 |
| Kresoxim Methyl | 92.0 | 99.1 | 99.5 | 95.4 |
| Malathion | 98.5 | 96.3 | 99.2 | 99.2 |
| Metalaxyl | 96.2 | 100.4 | 98.9 | 101.4 |
| Methiocarb | 100.0 | 95.2 | 98.9 | 97.2 |
| Methomyl | 97.1 | 95.1 | 97.8 | 97.2 |
| Methyl Parathion | 99.6 | 94.4 | 98.2 | 98.5 |
| (Z)-Mevinphos | 96.7 | 94.7 | 98.4 | 98.3 |
| (E)-Mevinphos | 99.4 | 94.8 | 97.8 | 98.7 |
| Myclobutanil | 99.2 | 97.3 | 98.3 | 98.6 |
| Naled | 98.9 | 95.3 | 99.3 | 98.8 |
| Oxamyl | 98.4 | 95.3 | 98.7 | 97.9 |
| Paclobutrazol | 96.4 | 95.2 | 98.0 | 98.1 |
| (E)-Permethrin | 135.5 | 145.5 | 157.3 | 165.7 |
| (Z)-Permethrin | 130.9 | 142.1 | 147.9 | 152.1 |
| Phosmet |  |  | 115.3* |  |
| Piperonyl Butoxide | 98.3 | 96.5 | 100.2 | 100.1 |
| Prallethrin | 103.8 | 102.1 | 105.7 | 105.1 |
| Propiconazole | 100.0 | 95.8 | 95.4 | 98.8 |
| Propoxur | 97.3 | 94.8 | 97.8 | 97.7 |
| Pyrethrin I | 109.7 | 119.9 | 119.3 | 124.8 |
| Pyrethrin II | 103.8 | 111.3 | 112.0 | 114.1 |
| Pyridaben | 96.1 | 99.5 | 109.2 | 111.8 |
| Spinetoram J | 104.3 | 109.2 | 111.3 | 110.7 |
| Spinetoram L | 104.3 | 108.6 | 115.6 | 111.7 |
| Spinosyn A | 103.9 | 103.7 | 109.9 | 108.5 |
| Spinosyn D | 107.2 | 110.3 | 114.1 | 114.9 |
| Spiromesifen | 93.2 | 92.5 | 114.0 | 99.9 |
| Spirotetramat | 95.8 | 96.6 | 108.7 | 101.0 |
| Spiroxamine | 98.6 | 94.8 | 99.2 | 97.7 |
| Tebuconazole | 99.3 | 96.4 | 98.2 | 98.6 |
| Thiacloprid | 98.4 | 95.9 | 97.6 | 98.0 |
| Thiamethoxam | 98.4 | 94.0 | 98.0 | 98.3 |
| Trifloxystrobin | 99.9 | 100.4 | 105.2 | 104.4 |

* n = 2

Supplementary Table 19. Matrix Effects - SIL-IS Compensation

| **Analyte** | **Average % Matrix effects** | **SIL-IS Compensated Average % ME** |
| --- | --- | --- |
| Acequinocyl (n = 2) | 155.7 | 88.9 |
| Bifenthrin (n = 22) | 198.5 | 110.1 |
| Captan (n = 2) | 134.8 | 108.1 |
| Cyfluthrin (n = 22) | 160.4 | 102.7 |
| Cypermethrin (n = 22) | 164.4 | 102.6 |
| Etofenprox (n = 22) | 128.6 | 100.1 |
| Jasmolin I (n = 22) | 124.9 | 98.0 |
| (E)-Permethrin (n = 22) | 151.0 | 107.7 |
| (Z)-Permethrin (n = 22) | 143.2 | 103.6 |

Supplementary Table 20. % Measurement Uncertainty across AMR (Major Component/Components) and at AL Spikes (100 ppb in-sample)

| **Analyte** | **Spike Level** | | | | | | | | | | | | | | | | | **120 AL** |
| --- | --- | --- | --- | --- | --- | --- | --- | --- | --- | --- | --- | --- | --- | --- | --- | --- | --- | --- |
|  | **1** | **2** | **3** | **4** | **5** | **6** | **7** | **8** | **9** | **10** | **11** | **12** | **13** | **14** | **15** | **16** | **17** |  |
| Imazalil | 18.94 | 17.02 | 11.92 | 10.98 | 2.81 | 2.71 | 4.76 | 2.28 | 2.32 | 1.36 | 1.35 | 2.3 | 1.36 | 2.17 | 2.38 | 1.57 | 8.08 | 3.45 |
| Spirotetramat | 19.07 | 18.3 | 16.61 | 13.86 | 7.13 | 3.71 | 4.12 | 2.88 | 4.71 | 2.33 | 3.22 | 3.57 | 3.25 | 2.42 | 3.21 |  | 9.33 | 4.14 |
| Azoxystrobin |  | 18.87 | 12.7 | 8.7 | 10.4 | 5.75 | 3.34 | 4.93 | 4.94 | 4.54 | 2.57 | 2.89 | 3.29 | 2.66 | 2.7 | 2.33 | 7.22 | 5.03 |
| Diazinon |  | 9.74 | 11.42 | 10.11 | 4.18 | 4.37 | 5.01 | 2.04 | 4.04 | 2.07 | 0.82 | 3.7 | 1.98 | 2.25 | 2.86 | 2.94 | 9.33 | 5.39 |
| Ethoprophos |  | 10.47 | 13.43 | 3.14 | 6.41 | 4.41 | 2.53 | 3.08 | 2.27 | 1.52 | 2.33 | 4.55 | 1.68 | 1.68 | 1.9 | 2.25 | 6.87 | 3.56 |
| Thiacloprid |  | 16.26 | 13.04 | 6.38 | 3.57 | 5.54 | 4.52 | 5.31 | 4.45 | 2.58 | 3.81 | 2.03 | 3.45 | 1.55 | 2.53 | 3.77 | 6.49 | 4.38 |
| Acephate |  |  | 9.69 | 11.4 | 10.52 | 9.46 | 7.27 | 4.41 | 3.68 | 5.63 | 2.86 | 2.58 | 2.87 | 2.35 | 3.49 | 6.29 | 7.65 | 4.93 |
| Acetamiprid |  |  | 11.61 | 10.12 | 6.6 | 6.28 | 5.7 | 3.34 | 3.08 | 3.26 | 2.53 | 2.43 | 3.23 | 2.96 | 4.37 | 1.41 |  | 4.12 |
| Clofentezine |  |  | 15.99 | 6.44 | 4.02 | 4.57 | 4.67 | 3.24 | 3.31 | 1.6 | 1.29 | 2.78 | 1.56 | 2.93 | 2.22 | 3.78 | 8.76 | 3.53 |
| Dimethoate |  |  | 20.55 | 18.83 | 6.83 | 4.68 | 6.03 | 3.25 | 3.97 | 2.61 | 4.45 | 6.92 | 2.47 | 4.55 | 9.44 | 3 | 7.22 | 7.3 |
| Flonicamid |  |  | 18.04 | 16.22 | 15.33 | 13.69 | 13.77 | 11.06 | 6.99 | 5.3 | 7.64 | 9.3 | 8.93 | 7.63 | 4.8 | 4.84 | 12.61 | 8.16 |
| Spiroxamine |  |  | 15.92 | 3.9 | 5.99 | 3.67 | 5.06 | 1.93 | 3.75 | 2.77 | 2.3 | 3.94 | 2.21 | 1.87 | 1.96 | 2.51 | 7.71 | 3.89 |
| Aldicarb |  |  |  | 17.52 | 7.55 | 11.16 | 5.71 | 6.56 | 10.42 | 3.86 | 7.59 | 3 | 7.76 | 3.81 | 7.21 | 6.81 | 10.51 | 5.87 |
| Boscalid |  |  |  | 12 | 3.48 | 5.65 | 6.23 | 3.85 | 2.94 | 4.51 | 2.22 | 2.93 | 3.63 | 4 | 5.69 | 2.62 | 4.45 | 7.91 |
| Carbofuran |  |  |  | 8.9 | 6.87 | 2.48 | 6.87 | 0.56 | 3.36 | 3.4 | 3.09 | 2.96 | 1.49 | 1.96 | 4.13 |  | 4.51 | 3.91 |
| Chlorpyrifos |  |  |  | 7.73 | 2.33 | 5.81 | 3.87 | 2.21 | 2.15 | 0.9 | 2.15 | 2.43 | 1.4 | 1.78 | 2.92 | 1.94 | 7.95 | 3.65 |
| Coumaphos |  |  |  | 10.52 | 3.81 | 4.76 | 3.72 | 2.1 | 2.37 | 2.31 | 3.01 | 2.31 | 1.69 | 1.59 | 4.42 | 3.1 | 6.95 | 3.96 |
| Fenoxycarb |  |  |  | 15.78 | 4.33 | 5.29 | 5.07 | 3.48 | 3.12 | 1.74 | 1.8 | 1.85 | 1.71 | 1.97 | 2.86 | 2.21 | 7.1 | 3.61 |
| Hexythiazox |  |  |  | 16.91 | 13.82 | 2.15 | 11.07 | 1.82 | 7.07 | 2.07 | 4.43 | 4.12 | 3.45 | 2.79 | 5.76 |  | 11.13 | 6.28 |
| Daminozide |  |  |  |  | 7.35 | 2.78 | 2.37 | 1.76 | 4.94 | 8.98 | 3.44 | 4.59 | 1.95 | 5.95 | 3.15 | 4.34 | 6.37 | 16.46 |
| Etofenprox |  |  |  |  | 17.14 | 7.78 | 5.05 | 3.3 | 3.03 | 2.58 | 3.08 | 3.21 | 2.98 | 2.3 | 3.08 | 2.62 | 10.61 | 4.96 |
| Etoxazole |  |  |  |  | 6.93 | 2.94 | 3.05 | 2.55 | 3.05 | 1.96 | 1.48 | 1.82 | 2.05 | 1.85 |  |  | 6.67 | 3.32 |
| Fenpyroximate |  |  |  |  | 5.42 | 2.62 | 4.57 | 3.73 | 3.94 | 2.2 | 3.22 | 2.91 | 1.75 | 5 | 2.04 |  | 7.97 | 4.83 |
| Fipronil |  |  |  |  | 16.46 | 10.68 | 6.69 | 4.55 | 5.77 | 3.36 | 4.19 | 3.48 | 2.83 | 2.29 | 4.9 |  |  | 6.03 |
| Kresoxim Methyl |  |  |  |  | 14.68 | 13.74 | 15.04 | 10.32 | 5.87 | 8.14 | 8.44 | 10.45 | 7.78 | 5.2 | 8.4 | 5.5 | 9.34 | 7.39 |
| Methiocarb |  |  |  |  | 8.87 | 10.02 | 6.33 | 3.35 | 2.35 | 2.52 | 2.19 | 1.68 | 1.32 | 3.63 | 2.04 |  | 9.01 | 4.27 |
| Myclobutanil |  |  |  |  | 5.4 | 7.31 | 4.43 | 3.19 | 3.39 | 5.95 | 2.42 | 2.55 | 2.5 | 2.33 | 3.79 |  | 9.79 | 5.03 |
| Propoxur |  |  |  |  | 3.49 | 1.42 | 3.82 | 3.7 | 3.15 | 0.55 | 2.3 | 3.21 | 2.7 | 2.47 | 3.78 | 3.31 | 4.1 | 3.8 |
| Spiromesifen |  |  |  |  | 19.42 | 16.75 | 13.22 | 9.37 | 12.85 | 12.66 | 8.25 | 10.88 | 8.87 | 11.64 |  |  | 14.86 | 10.47 |
| Acequinocyl |  |  |  |  |  | 16.48 | 6.07 | 4.65 | 6.14 | 3.91 | 2.99 | 5.61 | 2.97 | 1.29 | 3.44 | 2.77 | 14.05 | 7.14 |
| Dichlorvos |  |  |  |  |  | 8.83 | 4.9 | 5.18 | 1.92 | 1.44 | 5.03 | 6.37 | 1.78 | 2.06 | 1.73 | 1.3 | 6.97 | 5.68 |
| Fludioxonil |  |  |  |  |  | 11.06 | 7.76 | 5.99 | 6.27 | 7.82 | 5.12 | 6.9 | 4.5 | 3.37 | 3.04 | 5.19 | 13.08 | 6.84 |
| Paclobutrazol |  |  |  |  |  | 10.76 | 4.97 | 5.62 | 3.51 | 4.36 | 3.24 | 3.48 | 1.61 | 2.57 | 2.96 | 3.38 | 4.55 | 4.38 |
| Propiconazole |  |  |  |  |  | 3.14 | 4.04 | 1.64 | 2.44 | 1.77 | 1.45 | 1.24 | 1.77 | 2.6 | 2.77 | 2.41 | 8.48 | 4.16 |
| Tebuconazole |  |  |  |  |  | 3.89 | 5.52 | 3.41 | 2.98 | 1.63 | 1.69 | 1.53 | 2.45 | 2.63 | 2.94 | 4.05 | 6.98 | 3.98 |
| Trifloxystrobin |  |  |  |  |  | 5.98 | 7.13 | 5.5 | 2.77 | 2.25 | 3.56 | 4.28 | 3.57 | 2.47 | 2.19 | 1.86 | 6.96 | 5.35 |
| Bifenazate |  |  |  |  |  |  | 11.99 | 7.91 | 5.79 | 7.42 | 7.97 | 4.81 | 5.61 | 4.41 | 8.45 | 7.44 | 14.56 | 15.49 |
| Fenhexamid |  |  |  |  |  |  | 7.9 | 4.74 | 4.85 | 3.55 | 4.57 | 2.49 | 3.84 | 3.19 |  |  |  | 6.19 |
| Naled |  |  |  |  |  |  | 4.65 | 3.43 | 3.88 | 2.53 | 3 | 6.17 | 1.93 | 3.9 | 1.57 | 3.03 | 6.77 | 9.05 |
| Phosmet |  |  |  |  |  |  | 3.35 | 5.06 | 5.57 | 3.09 | 1.94 | 1.94 | 2.34 | 3.24 | 2.84 | 3.28 | 9.06 | 5.24 |
| Prallethrin |  |  |  |  |  |  | 4.55 | 3.85 | 4.75 | 2.36 | 4.58 | 4.08 | 3.38 | 3.63 | 3.14 | 3.35 |  | 4.49 |
| Pyridaben |  |  |  |  |  |  |  | 4.6 | 3.32 | 2.03 | 3.28 | 2.49 | 1.58 | 5.32 |  |  | 7.67 | 7.24 |
| Methyl Parathion |  |  |  |  |  |  |  |  | 22.73 | 11.85 | 14.28 | 15.9 | 8.03 | 8.89 | 4.42 | 4.36 | 13.81 | 22.04 |
| Chlorfenapyr |  |  |  |  |  |  |  |  |  | 15 | 14.39 | 16.15 | 15.56 | 9.49 | 12.42 | 10.96 | 23.94 | 62.18 |
| Malathion |  | 18.57 | 15.1 | 5.19 | 6.63 | 5.01 | 4 | 3.64 | 3.4 | 3.7 | 2.07 | 1.76 | 2.74 | 2.23 | 3.91 | 2.57 | 9.49 | 4.12 |
| Carbaryl |  |  | 5.29 | 11.03 | 3.74 | 5.35 | 2.4 | 3.08 | 3.69 | 2.64 | 1.14 | 3.33 | 2.57 | 1.6 | 3.36 | 3.16 | 8.66 | 3.68 |
| Oxamyl |  |  | 5.21 | 5.02 | 4.3 | 4.04 | 3.94 | 5.13 | 2.34 | 3.91 | 2.24 | 2.09 | 4.04 | 2.71 | 3.57 | 2.27 | 7.84 | 3.93 |
| Captan |  |  |  |  |  |  |  |  |  |  |  | 15.22 | 13.86 | 12.2 | 16.35 | 15.22 | 24.18 |  |
| Methomyl |  |  |  | 18.65 | 14.31 | 7.67 | 8.26 | 4.73 | 5.45 | 1.81 | 3.37 | 3.89 | 3.42 | 3.9 | 3.74 | 5.42 | 5.66 | 5.83 |
| Cypermethrin |  |  |  |  |  |  |  |  | 11.54 | 2.42 | 1.53 | 2.19 | 3.32 | 2.26 | 2.79 | 1.43 | 6.58 | 8.33 |
| Metalaxyl |  |  |  | 8.08 | 5.06 | 2.53 | 4.45 | 2.8 | 3.49 | 3.05 | 2.6 | 3.85 | 4.5 | 2.63 | 3.83 | 1.01 | 6.11 | 4.07 |
| Cyfluthrin |  |  |  |  |  |  |  |  |  | 9.95 | 7.57 | 8.25 | 5.05 | 4.16 | 3.78 | 7.98 | 12.22 | 17.07 |
| Piperonyl Butoxide |  |  |  | 6.5 | 5.36 | 5.92 | 4.85 | 2.68 | 3.2 | 1.22 | 2.93 | 3.24 | 3.1 | 3.21 | 3.66 | 2.42 | 9.59 | 6.24 |
| Bifenthrin |  |  |  |  |  |  | 15.44 | 10.41 | 8.61 | 4.12 | 2.88 | 11.3 | 3.29 | 2.77 | 12.65 | 9.01 |  | 10.41 |
| Imidacloprid | 21.22 | 12.85 | 4.26 | 4.75 | 4.88 | 4.64 | 1.76 | 3.27 | 2.88 | 1.83 | 1.46 | 1.1 | 2.39 | 2.02 | 3.29 | 4.25 | 6.33 | 3.28 |
| Thiamethoxam |  |  | 12.18 | 4.84 | 7.95 | 5.82 | 5.87 | 4.5 | 5.43 | 3.79 | 1.61 | 4.42 | 3.97 | 4.22 | 3.45 | 3.48 | 6.29 | 5.18 |
| Chlorantraniliprole |  |  |  |  | 5 | 7.93 | 7.7 | 4.67 | 4.53 | 4.36 | 4.08 | 3.58 | 2.32 | 3.12 | 4.64 | 4.44 | 6.91 | 5.6 |
| (E)-Mevinphos | 20.75 | 17.08 | 10.48 | 11.24 | 5.3 | 4.32 | 4.54 | 3.55 | 4.54 | 2.69 | 3.51 | 7.37 | 2.74 | 3.77 | 4.46 | 2.8 | 7.6 | 3.69 |
| (Z)-Mevinphos |  |  |  |  | 18.11 | 6.17 | 6.5 | 4.27 | 4.87 | 2.88 | 5.42 | 5.25 | 2.87 | 5.64 | 3.32 | 4.61 | 6.05 | 3.69 |
| Spinetoram J |  |  |  | 13.66 | 8.01 | 10.5 | 13.72 | 9.39 | 5.19 | 4.49 | 6.44 | 6.28 | 4.7 | 14.2 | 4.15 | 6.44 | 6.81 | 11.14 |
| Spinetoram L |  |  |  |  |  | 11 | 11.75 | 7.5 | 4.2 | 5.5 | 4.24 | 7.45 | 3.5 | 12.62 | 5.06 | 4.47 | 11.05 | 11.14 |
| Spinosyn A |  |  |  |  | 4.07 | 5.09 | 14.72 | 4.36 | 3.75 | 4.4 | 4.87 | 7.49 | 3.42 | 11.65 | 4.3 |  | 8.05 | 10.02 |
| Spinosyn D |  |  |  |  |  |  |  | 7.48 | 9.06 | 3.94 | 9.64 | 9.44 | 6.59 | 16.45 | 5.82 | 3.38 |  | 10.02 |
| Avermectin B1a |  |  |  |  |  |  |  |  | 13.52 | 12.19 | 9.37 | 10.38 | 7.94 | 5.2 | 10.09 |  | 23.3 | 24.38 |
| Avermectin B1b |  |  |  |  |  |  |  |  |  |  |  | 13.19 | 11.78 | 5.74 | 10.75 | 6.24 |  | 24.38 |
| Cinerin I |  |  |  |  |  |  | 10.71 | 8.96 | 7.51 | 4.61 | 7.36 | 8.19 | 3.4 | 4.3 | 3 | 3.44 |  | 7.92 |
| Cinerin II |  |  |  |  |  |  |  | 15.38 | 13.85 | 6.1 | 6.76 | 4.11 | 4.29 | 3.4 | 3.37 | 3.6 |  | 7.92 |
| Jasmolin I |  |  |  |  |  |  |  |  | 14.35 | 12.13 | 10.66 | 6.06 | 8.56 | 7.77 | 4.88 |  |  | 7.92 |
| Jasmolin II |  |  |  |  |  |  |  |  |  | 2.61 | 8.25 | 9 | 7.1 | 4.61 | 8.01 | 6.28 |  | 7.92 |
| Pyrethrin I |  |  |  |  |  |  | 18.09 | 10.15 | 8.87 | 7.51 | 5.2 | 8.35 | 7.87 | 3.41 | 6.81 | 8.64 |  | 7.92 |
| Pyrethrin II |  |  |  |  |  |  |  | 6.86 | 5.67 | 6.57 | 10.14 | 7.01 | 9.8 | 3.27 | 6.14 | 6.97 |  | 7.92 |
| (E)-Permethrin |  |  |  |  |  | 13.21 | 5.36 | 4.71 | 3.77 | 3.29 | 4.7 | 2.98 | 2.93 | 3.48 | 3.56 | 4.24 | 10.43 | 6.05 |
| (Z)-Permethrin |  |  |  |  |  |  |  | 4.66 | 3.94 | 2.42 | 1.75 | 3.09 | 1.95 | 2.52 | 4.14 | 3.97 | 12.58 | 6.05 |
| (E)-Dimethomorph |  |  |  |  |  | 2.1 | 6.11 | 2.82 | 3.97 | 1.79 | 3.16 | 3.71 | 1.85 | 1.46 | 4.18 | 2.14 | 8.65 | 5.1 |
| (Z)-Dimethomorph |  |  |  |  | 3.89 | 4.15 | 7.39 | 2.39 | 3.75 | 2.59 | 1.95 | 3.36 | 1.89 | 2.23 | 0.96 | 3.38 | 7.41 | 5.1 |

Supplementary Table 21. Surrogate IS Contiguous Linear Regression Fittings

| See Supplementary Table 21 | (Excel File) |
| --- | --- |

Supplementary Table 22. Surrogate ISs ≤ 20% Max Absolute Bias for Individual Components of the Pyrethrins

| **Surrogate IS** | **Max Absolute Bias** |
| --- | --- |
| ***Cinerin I*** | |
| Chlorpyrifos-d_10_ | 16.643 |
| Imazalil-d_5_ | 19.358 |
| Pyridaben-d_13_ | 18.813 |
|  |  |
| ***Cinerin II*** | |
| Allethrin | 17.951 |
| Cyfluthrin-d_6_ | 18.242 |
| Cypermethrin-d_9_ | 18.577 |
| Danitol | 18.754 |
| Etoxazole-d_5_ | 17.674 |
|  |  |
| ***Jasmolin I*** | |
| N/A |  |
|  |  |
| ***Jasmolin II*** | |
| Allethrin | 17.720 |
| Chlorpyrifos-d_10_ | 18.071 |
| Clofentazine-d_8_ | 15.952 |
| Cyfluthrin-d_6_ | 17.404 |
| Cypermethrin-d_9_ | 16.880 |
| Danitol | 14.718 |
| Etoxazole-d_5_ | 19.001 |
| Piperonyl Butoxide-d_9_ | 17.285 |
|  |  |
| ***Pyrethrin I*** | |
| Chlorpyrifos-d_10_ | 16.692 |
| Danitol | 19.860 |
| Hexyziathox-d_11_ | 17.482 |
| Pyridaben-d_13_ | 19.029 |
|  |  |
| ***Pyrethrin II*** | |
| Coumaphos-d_10_ | 15.914 |
| Fenoxycarb-d_3_ | 15.430 |
| Fludioxonil-^13^C_3_ | 19.768 |
| Kresoxim Methyl-d_7_ | 19.710 |
| Trifloxystrobin-d_6_ | 18.032 |

Supplementary Table 23. R^2^ and Root Mean Square Error (%) for precision-based MU model. The fitness of the 2*k*SEM regression model was evaluated against pseudo-random samplings of the 120 AL-spikes (at 100 ppb in-sample). Sampling at n=1-120 was used to simulate the empirical behavior of MUs with increasing replicate measurements.

| **Analyte** | **%CV** | **R^2^** | **%RMSE** |
| --- | --- | --- | --- |
| Trifloxystrobin | 2.67 | 0.7302 | 0.3132 |
| Acetamiprid | 2.05 | 0.7706 | 0.4156 |
| Imazalil | 1.72 | 0.7779 | 0.2165 |
| Daminozide | 8.19 | 0.7937 | 0.8974 |
| Tebuconazole | 1.98 | 0.7972 | 0.2104 |
| Spinosad | 4.99 | 0.8046 | 0.5434 |
| Clofentezine | 1.76 | 0.8091 | 0.2139 |
| Naled | 4.51 | 0.8258 | 0.4679 |
| Propiconazole | 2.07 | 0.8300 | 0.2370 |
| Thiacloprid | 2.18 | 0.8306 | 0.2369 |
| Fludioxonil | 3.41 | 0.8370 | 0.3428 |
| Dichlorvos | 2.83 | 0.8383 | 0.2836 |
| Imidacloprid | 1.63 | 0.8386 | 0.1682 |
| Coumaphos | 1.97 | 0.8397 | 0.2425 |
| Mevinphos | 1.84 | 0.8402 | 0.1911 |
| Chlorpyrifos | 1.82 | 0.8405 | 0.1843 |
| Fenpyroximate | 2.41 | 0.8425 | 0.2398 |
| Propoxur | 1.89 | 0.8432 | 0.1950 |
| Carbaryl | 1.83 | 0.8435 | 0.1849 |
| Diazinon | 2.68 | 0.8463 | 0.2695 |
| Bifenazate | 7.72 | 0.8464 | 0.7689 |
| Dimethoate | 3.64 | 0.8486 | 0.3553 |
| Dimethomorph | 2.54 | 0.8512 | 0.2449 |
| Prallethrin | 2.24 | 0.8545 | 0.2193 |
| Cyfluthrin | 8.50 | 0.8553 | 0.8409 |
| Azoxystrobin | 2.51 | 0.8560 | 0.2555 |
| Chlorfenapyr | 30.96 | 0.8561 | 3.1056 |
| Ethoprophos | 1.77 | 0.8569 | 0.1782 |
| Piperonyl Butoxide | 3.10 | 0.8586 | 0.3167 |
| Fenoxycarb | 1.80 | 0.8606 | 0.1773 |
| Fenhexamid | 3.08 | 0.8608 | 0.3019 |
| Spiroxamine | 1.94 | 0.8615 | 0.1972 |
| Etofenprox | 2.47 | 0.8623 | 0.2373 |
| Fipronil | 3.00 | 0.8650 | 0.3013 |
| Kresoxim methyl | 3.68 | 0.8650 | 0.4184 |
| Cypermethrin | 4.15 | 0.8651 | 0.4076 |
| Methyl Parathion | 10.98 | 0.8684 | 1.0286 |
| Paclobutrazol | 2.18 | 0.8715 | 0.2095 |
| Acephate | 2.46 | 0.8717 | 0.2387 |
| Spinetoram | 5.55 | 0.8717 | 0.5382 |
| Hexythiazox | 3.13 | 0.8758 | 0.2900 |
| Permethrin | 3.01 | 0.8758 | 0.2856 |
| Pyridaben | 3.61 | 0.8761 | 0.3404 |
| Spiromesifen | 5.21 | 0.8778 | 0.4701 |
| Abamectin | 12.14 | 0.8795 | 1.1533 |
| Chlorantraniliprole | 2.79 | 0.8807 | 0.2568 |
| Pyrethrins | 3.94 | 0.8825 | 0.3648 |
| Metalaxyl | 2.03 | 0.8835 | 0.1858 |
| Etoxazole | 1.65 | 0.8836 | 0.1835 |
| Boscalid | 3.94 | 0.8846 | 0.3709 |
| Thiamethoxam | 2.58 | 0.8871 | 0.2416 |
| Carbofuran | 1.95 | 0.8873 | 0.1890 |
| Oxamyl | 1.95 | 0.8889 | 0.1778 |
| Malathion | 2.05 | 0.8890 | 0.1916 |
| Aldicarb | 2.92 | 0.8914 | 0.2839 |
| Methomyl | 2.90 | 0.8917 | 0.2703 |
| Acequinocyl | 3.55 | 0.8957 | 0.3167 |
| Methiocarb | 2.12 | 0.8975 | 0.1983 |
| Bifenthrin | 5.18 | 0.8976 | 0.4872 |
| Phosmet | 2.61 | 0.8979 | 0.2401 |
| Flonicamid | 4.06 | 0.9009 | 0.3550 |
| Spirotetramat | 2.06 | 0.9016 | 0.1843 |
| Myclobutanil | 2.50 | 0.9086 | 0.2266 |
